# Inhibition of lagging strand replication by G-rich telomeric DNA and the shelterin subunit POT1

**DOI:** 10.1038/s44318-026-00844-7

**Published:** 2026-07-09

**Authors:** Ciara Leonard-Booker, Giulia Mazzucco, Charlotte E L Fisher, Karim Hussain, Tom D Deegan, Ylli Doksani, Max E Douglas

**Affiliations:** 1 Telomere Biology Laboratory, The Institute of Cancer Research, London, UK; 2 IFOM ETS-The AIRC Institute of Molecular Oncology, Milan, Italy; 3 MRC Human Genetics Unit, University of Edinburgh, Edinburgh, UK; 4 Present Address: 4bases Italia S.r.l.. Viale A. Brambilla 60, Pavia, Italy

**Keywords:** DNA Replication, Recombination & Repair

## Abstract

Telomeres preserve stable eukaryotic chromosomes by protecting the natural chromosome ends from DNA repair but pose a persistent challenge to the replication machinery and an endogenous source of replication stress. Different features have been implicated in causing this effect but how the canonical replication process is altered at telomeres remains poorly understood. To address this question, we have reconstituted telomere replication with purified human proteins. Our system reveals that G-rich telomeric DNA can directly and specifically block lagging strand replication in a manner counteracted by BLM helicase. Unexpectedly, we also identify shelterin as inhibitory for the lagging strand. Biochemical experiments and electron microscopy imaging show that POT1-containing shelterin complexes induce Okazaki fragment skipping by binding the lagging strand template, generating large single-stranded gaps that are left behind on otherwise fully replicated molecules. Our study defines how the core components of telomeres interfere with the canonical replication process, identifying multiple potential sources of replication stress.

## Synopsis

Telomeres are known to act as an endogenous source of replication stress. This study examines how the core components of telomeres influence the replication process by combining a reconstituted human replisome with telomeric templates, uncovering several inhibitory effects.

- G- and C-rich telomeric DNA are each efficiently copied as the leading strand by the human replisome.
- G-rich telomeric can directly inhibit lagging strand replication in a manner that is enhanced by G4-stabilising agents and prevented by BLM helicase.
- Shelterin can act as a replication roadblock but also induces lagging-strand gaps through binding of POT1 to unwound, G-rich telomeric DNA.

## Abstract (web summary)

Reconstituted human replisomes with telomeric templates reveal how telomeres may act as endogenous sources of replication stress.

## Graphical abstract

**Figure:**
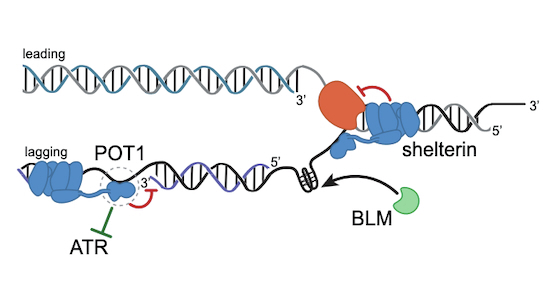

**Figure:**
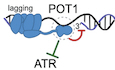

## Introduction

In human cells, stable linear chromosomes are maintained by telomeres composed of 10-15 kilobases of the repetitive sequence TTAGGG and shelterin, a six-subunit protein complex comprising TRF1, TRF2, RAP1, TIN2, TPP1 and POT1 (de Lange, 2026; Lim and Cech, 2021). Shelterin binds tightly to double-stranded telomeric DNA through TRF1 and TRF2 and single-stranded G-rich repeats via POT1, forming a nucleoprotein assembly that blocks inappropriate DNA repair processes including homologous recombination (HR), non-homologous end joining (NHEJ) and checkpoint activation (de Lange, 2026). Loss of these functions at even a single chromosome end can disrupt chromosomal structure, driving cell cycle arrest or cell death (Muraki et al, 2012).

In dividing cells, most telomeric DNA is maintained by semiconservative DNA replication. Recent advances have established a canonical view of the replication process in which the replicative Cdc45, Mcm2-7 and GINS (CMG) helicase unwinds parental DNA by translocating 3’–5’ along the leading strand template in conjunction with its associated proteins Claspin, Tipin-Timeless and AND-1 (Burgers and Kunkel, 2017; Jones et al, 2021; Moyer et al, 2006; Rzechorzek et al, 2020; Simon et al, 2014). Continuous leading strand synthesis is mediated by Polymerase ε (Pol ε), while discontinuous lagging strand synthesis by Polymerase α/primase (Pol α/prim) and Polymerase δ (Pol δ) is followed by maturation of Okazaki fragments (OFs) into a continuous product through a set of factors including Flap Endonuclease 1 (FEN1) and DNA Ligase 1 (LIG1) (Burgers and Kunkel, 2017; Deegan et al, 2019; Georgescu et al, 2015; Jones et al, 2023; Yeeles et al, 2017).

Within the nuclear context, this process must contend with complex templates including those bound to proteins, assembled into secondary structures or containing features such as R-loops, which can cause fork stalling events and replication defects collectively termed replication stress (Zeman and Cimprich, 2014). In mammalian cells, genomic regions prone to replication stress have been identified as ‘fragile sites’ that are sensitive to low concentrations of replication inhibitors (Durkin and Glover, 2007). Telomeres act as fragile sites in mouse and human cells (Glousker and Lingner, 2021; Huda et al, 2023; Sfeir et al, 2009) and can induce replication defects in budding and fission yeasts (Ivessa et al, 2002; Miller et al, 2006), suggesting they pose conserved challenges to DNA replication across eukaryotes, with the potential for telomere loss and chromosome deprotection unless these challenges are handled effectively.

An extensive number of studies have implicated a range of telomeric properties in this effect (reviewed in (Maestroni et al, 2017) and (Douglas, 2024)), paradoxically including the components that endow telomeres with their protective functions: TTAGGG repeats can fold into G-quadruplex (G4) structures that hinder replicative polymerases and the CMG helicase in vitro (Batra et al, 2025; Kumar et al, 2021; Tran et al, 2011; Williams et al, 2023) and mutations in G4-unwinding helicases or treatment with G4-stabilising ligands causes fragile telomeres in mouse and human cells, consistent with sequence playing an inhibitory role (Crabbe et al, 2004; Drosopoulos et al, 2015; PREPRINT: Matmati et al, 2025; Sun et al, 1998; Vannier et al, 2013; Wu et al, 2015; Zimmermann et al, 2014). The shelterin complex, which is involved in essentially all protective telomeric functions, can act as a roadblock to viral replisomes (Leman et al, 2012; Ohki and Ishikawa, 2004), suggesting that in addition to recruiting factors that promote telomere replication (Sarek et al, 2019; Sarek et al, 2015; Sfeir et al, 2009; Yang et al, 2022; Zimmermann et al, 2014), shelterin may also prove inhibitory. While these findings hint at a complex interplay between the replication machinery, shelterin and telomeric DNA, how different components of telomeres affect the replisome in mechanistic terms remains poorly understood. Here, we seek to address this question by reconstituting telomere replication with purified human proteins. Our results reveal distinct impacts of the core components of telomeres on the canonical replication process, uncovering a potent inhibitory effect of the shelterin subunit POT1 on the lagging strand.

## Results

### Telomeric DNA does not appreciably inhibit leading strand replication

**Figure 1:**
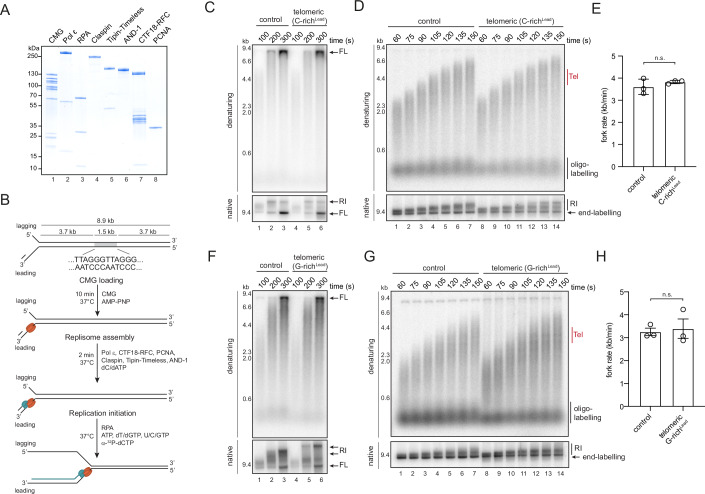
Human telomeric DNA does not inhibit leading strand synthesis or replisome progression. (**A**) 4–15% Tris-Glycine SDS-PAGE analysis of purified human replisome factors. (**B**) Schematic for in vitro replication reactions on a forked DNA template. The position and orientation of telomeric DNA is highlighted. (**C**) Replication reactions performed with a template containing 240 TTAGGG repeats or a non-telomeric control sequence for the times indicated were analysed by native and denaturing alkaline agarose electrophoresis. Full-length (FL) and replication intermediates (RI) are indicated. (**D**) Pulse chase analysis with a template containing 240 TTAGGG repeats or a non-telomeric control sequence as indicated. Chase containing an excess of non-radiolabelled dNTPs was added 50 s after the initiation mix and samples taken at the times indicated after initiation and analysed by native and denaturing alkaline agarose electrophoresis. Site of telomeric insert indicated (Tel). Oligo-labelling refers to CMG-independent labelling of the oligonucleotide used to initiate leading strand synthesis. (**E**) Quantification of maximal fork rates from pulse chase reactions as in (**D**). Error bars show S.E.M. *N* = 3 (three independent replication assays). Unpaired *t* test used to determine statistical significance (*P* = 0.089). (**F**–**H**) As for (**C**–**E**), except the orientation of the telomeric insert was reversed such that the G-rich sequence is the template for leading strand replication. (*P* = 0.76). Source data are available online for this figure.

**Figure EV1:**
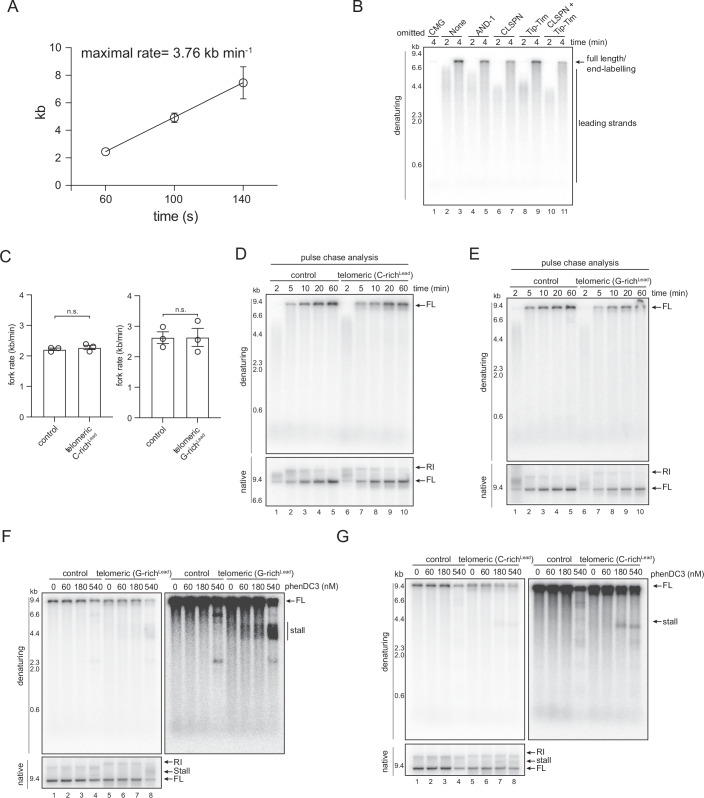
Supplementary data for Fig. 1. (**A**) Quantification of the maximal leading strand synthesis rates from denaturing alkaline agarose electrophoresis. Linear regression is fit to the average of three independent experiments and error bars show S.E.M. (**B**) Replication reactions performed with non-telomeric control template for the indicated times with proteins omitted as indicated. Products analysed by denaturing alkaline agarose electrophoresis. (**C**) Quantification of bulk fork rates from pulse chase reactions as in Fig. 1D,G. Error bars show S.E.M. *N* = 3 (three independent replication assays). Unpaired *t* test used to determine statistical significance. Corresponding *P* values: control vs telomeric C-rich Lead (0.51), control vs telomeric G-rich Lead (0.99). (**D**) Pulse chase analysis with a template containing 240 TTAGGG repeats or a non-telomeric control sequence. Chase of non-radiolabelled dNTPs was added after 50 s. Samples taken at the indicated times after initiation were analysed by native and denaturing alkaline agarose electrophoresis (**E**) As for (**D**), except comparing a template containing a non-telomeric control or 240 TTAGGG repeats oriented with G-rich telomeric DNA as the sequence for leading strand replication. (**F**) Products from replication reactions performed for 15 min with templates containing a non-telomeric control sequence or 240 TTAGGG repeats where G-rich telomeric DNA is the sequence for leading strand replication and with the indicated concentrations of phenDC3 were analysed as in (**B**). (**G**) As for (**E**), except comparing a template containing a non-telomeric control sequence or 240 TTAGGG repeats where C-rich telomeric DNA is the sequence for leading strand replication.

To examine how different aspects of DNA replication are affected by telomeres, we set out to combine a previously reconstituted assay for human DNA replication with templates containing different telomeric properties. In this system (Baris et al, 2022), a set of eight purified factors (Fig. 1A) is used to build a human replisome on forked DNA templates that support CMG- and Pol ε-dependent leading strand synthesis monitored by incorporation of α-^32^P-labelled dCTP. Following staged addition of replication factors and incubation at 37° C (Fig. 1B), reaction products were deproteinated and resolved by denaturing and native agarose electrophoresis, revealing synthesis of fully replicated (~9 kb) leading strands, and resolution of replication intermediates into linear molecules within 5 min (Fig. 1C, lanes 1–3). Pulse-chase assays, in which we added an excess of unlabelled dNTPs 50 s after initiation, revealed a maximal replication rate of 3.8 kb/min in vitro (Fig. EV1A), which slowed without AND-1, Claspin or Tipin-Timeless (Fig. EV1B), consistent with previous work (Baris et al, 2022).

The repetitive, GC-rich nature of telomeres is widely proposed to inhibit DNA replication, yet whether telomeric sequence affects specific aspects of the replication process and how remains poorly understood. To address these questions, we inserted a 1.5 kb tract of TTAGGG repeats into our replication molecule oriented with the C-rich strand as the leading strand template, reflecting the configuration of most telomere replication events in human cells (Drosopoulos et al, 2012; Sfeir et al, 2009) (Fig. 1B). Replication across this telomeric template was indistinguishable from a non-telomeric control sequence (Fig. 1C, compare lanes 1–3 and 4–6) and pulse chase assays showed no change in the maximum or bulk replication rates or the profile of products (Figs. 1D,E and EV1C,D). C-rich telomeric DNA can therefore be efficiently copied as the leading strand template.

In mammalian cells, DNA replication occasionally initiates within the telomeric repeats, generating forks on which the G-rich strand supports leading strand synthesis (Drosopoulos et al, 2015; Drosopoulos et al, 2012). Other G-rich sequences in this scenario have been shown to stall the leading strand through the formation of G4s (Kumar et al, 2021; Williams et al, 2023). However, reversing the orientation of telomeric repeats in our system had no appreciable effect on the rate of replication or profile of products (Figs. 1F–H and  EV1C,E). Moreover, adding the G4-stabilising agent phenDC3 induced only a modest accumulation of telomere-stalled products unless high concentrations that also inhibit C-strand replication were used (Fig. EV1F,G). Therefore, G-rich telomeric DNA can also be efficiently copied as the template for the leading strand.

### G-rich telomeric DNA prevents complete replication of the lagging strand

While leading strand synthesis is continuous and coupled to the CMG helicase (Goswami et al, 2018; Xu et al, 2023), lagging strand replication is discontinuous and uncoupled from CMG, transiently exposing stretches of single-stranded DNA that are prone to form secondary structures (Sethi et al, 2025).

**Figure 2:**
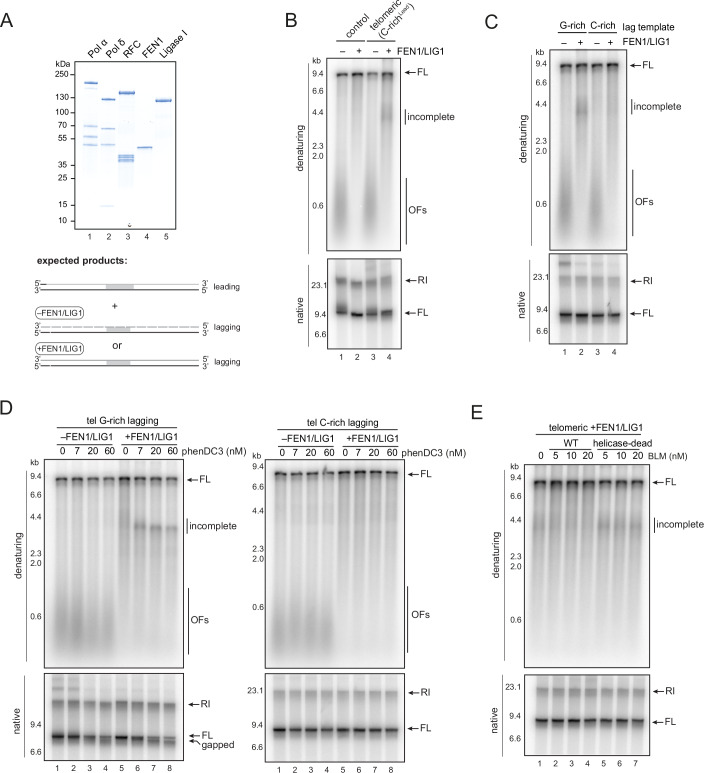
G-rich telomeric DNA can directly inhibit lagging strand replication. (**A**) 4–15% Tris-Glycine SDS-PAGE analysis of purified human lagging strand replication factors and schematic showing expected leading and lagging strand products with and without FEN1/LIG1. (**B**) Replication reactions performed for 30 min in the presence or absence of FEN1/LIG1 with a template containing 240 TTAGGG repeats or a non-telomeric control sequence as indicated were analysed by native or denaturing alkaline agarose electrophoresis. Okazaki fragments (OFs) are indicated. (**C**) Products from replication reactions performed for 30 min in the presence or absence of FEN1/LIG1 with templates containing 240 TTAGGG repeats oriented with the G- or C-rich strands as the lagging strand template as indicated were analysed as in (**B**). (**D**) Products from replication reactions performed for 30 min in the presence or absence of FEN1/LIG1 and the indicated concentrations of phenDC3 with templates containing 240 TTAGGG repeats oriented with the G-rich (left) or C-rich (right) strands as the lagging strand template were analysed as in (**B**). (**E**) Products from replication reactions performed for 30 min with a template containing 240 TTAGGG repeats in the presence of FEN1/LIG1 and the indicated concentrations of wild-type (‘WT’) or helicase-inactive (‘helicase-dead’) BLM were analysed as in (**B**). Lane scans for this figure are available in Appendix Fig. S1. Source data are available online for this figure.

**Figure EV2:**
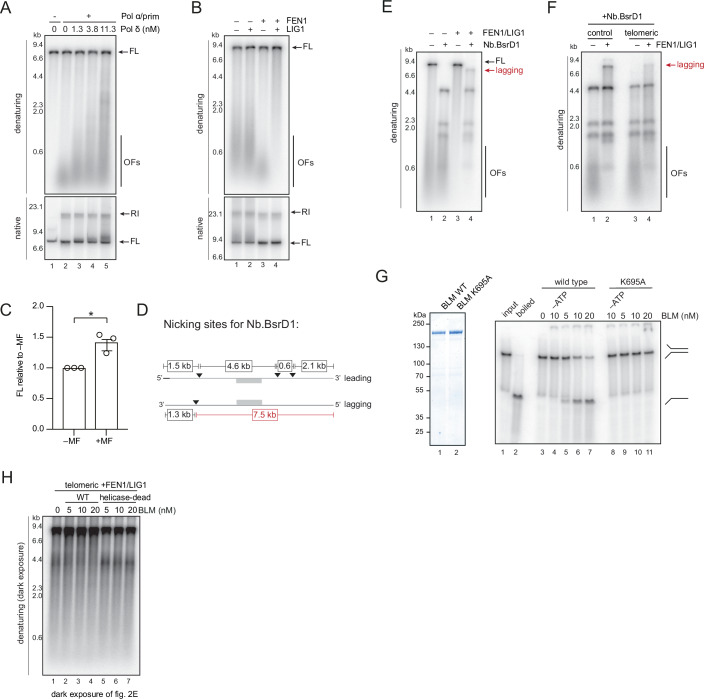
Supplementary data for Fig. 2. (**A**) Replication reactions performed for 20 min with a non-telomeric control template in the presence or absence of the indicated proteins. Products were analysed by native and denaturing alkaline agarose electrophoresis as indicated. (**B**) Products from replication reactions performed for 30 min with a non-telomeric control template in the presence or absence of FEN1 or LIG1 as indicated were analysed as in A. (**C**) Quantification showing full length signal in the presence of maturation factors (MF) relative to their absence. Error bars show the S.E.M. *N* = 3 (three independent replication assays). Unpaired *t* test used to determine statistical significance (*P* = 0.0101). (**D**) Schematic shows position of Nb.BsrDI sites and products expected from nicking of leading and lagging strand products. (**E**) Products from replication reactions performed for 30 min with a non-telomeric control template in the presence or absence of FEN1/LIG1 as indicated were treated with Nb.BsrDI for 2 h and analysed by denaturing alkaline agarose electrophoresis. Position of product indicating fully-matured lagging strand is shown. (**F**) Products from replication reactions performed for 30 min with templates containing 240 TTAGGG repeats or a non-telomeric control sequence in the presence or absence of FEN1/LIG1 as indicated were treated with Nb.BsrDI for 2 h and analysed by denaturing alkaline agarose electrophoresis. (**G**) 4–15% Tris-Glycine SDS-PAGE analysis of purified wild-type (‘wt’) and helicase-dead (K695A) BLM. Products from helicase assays performed for 10 min on 1.5 nM duplexed substrate with the indicated concentrations of BLM wt or helicase dead were analysed by native agarose electrophoresis. (**H**) Dark exposure of Fig. 2E.

With this property in mind, we set out to test whether the lagging strand was more sensitive to telomeric DNA by incorporating lagging strand replication factors into our reactions (Fig. 2A). On a control template, addition of purified RFC1-5 and Pol α/prim generated OFs 200–500 nt long that were extended in the presence of Pol δ, as previously described (Baris et al, 2022) (Fig. EV2A). Inclusion of FEN1 and LIG1 converted these fragments into unit length products, suggesting they had been successfully processed and ligated together (Figs. 2B, lanes 1 and 2 and  EV2B,C). We confirmed this was the case by cutting replication products with a strand-biased nickase, revealing full length lagging strands only when FEN1 and LIG1 were added (Fig. EV2D,E).

Replicating the telomeric template in this system revealed a collection of products 3.5–4 kb in length, suggesting lagging strand replication over the telomere was defective (Fig. 2B, ‘incomplete’ products, compare lanes 2 and 4. Figure 1B for template organisation). The nickase assay above confirmed a reduced level of fully replicated lagging strands on the telomeric compared with the control template, consistent with this idea (Fig. EV2F). Strikingly, equivalent products were not observed when the orientation of the telomeric repeats was reversed (Fig. 2C, compare lanes 2 and 4), suggesting a specific lagging strand defect on G-rich telomeric DNA. To test whether this reflects an inhibitory effect of G4s, we added phenDC3. While defective products on G-rich telomeric DNA alone were somewhat broad and variable (see experiments across Fig. 2), even low concentrations of phenDC3 induced a sharp band of defective lagging strand products on G- but not C-rich telomeric DNA (Fig. 2D). Native agarose electrophoresis, where nascent and template strands remain annealed, revealed a population of products migrating faster than the full length under these conditions, consistent with the formation of ‘gapped’ molecules and a penetrant defect in DNA synthesis when G4s were stabilised (Fig. 2D, bottom panel, lane 8 ‘gapped’ product).

Genetic studies have shown that efficient lagging strand replication at telomeres depends on the RecQ helicase BLM (Sfeir et al, 2009; Zimmermann et al, 2014). As BLM can unwind G4s (Huber et al, 2002; Sun et al, 1998; Wu et al, 2015) we tested whether it could alleviate the inhibitory effect above. Addition of purified BLM but not a helicase inactive mutant decreased the level of replication defects over the telomere (Figs. 2E and  EV2G,H), consistent with a previous model that the DNA unwinding activity of BLM prevents the inhibitory effect of telomeric DNA on the G-rich lagging strand (Zimmermann et al, 2014).

From this analysis, we conclude that while both strands of telomeric DNA can be efficiently copied as the leading strand, the G-rich strand can directly impair lagging strand replication in a manner that is strongly enhanced by stabilising G4s and prevented by BLM.

### Shelterin can stall the human replisome

We next turned our attention to the main protein factor bound to telomeres in human cells: the shelterin complex. Estimates of shelterin density suggest the replisome encounters tens to hundreds of shelterin molecules at each human telomere (Janovič et al, 2025; Takai et al, 2010). While previous work has shown that DNA-bound shelterin acts as a roadblock for a viral replisome (Leman et al, 2012; Ohki and Ishikawa, 2004), telomere replication is impaired rather than promoted in mouse or human cells lacking individual shelterin subunits (Martínez et al, 2009; Sfeir et al, 2009). Whether shelterin has an equivalent effect on the human replisome is therefore unclear.

**Figure 3:**
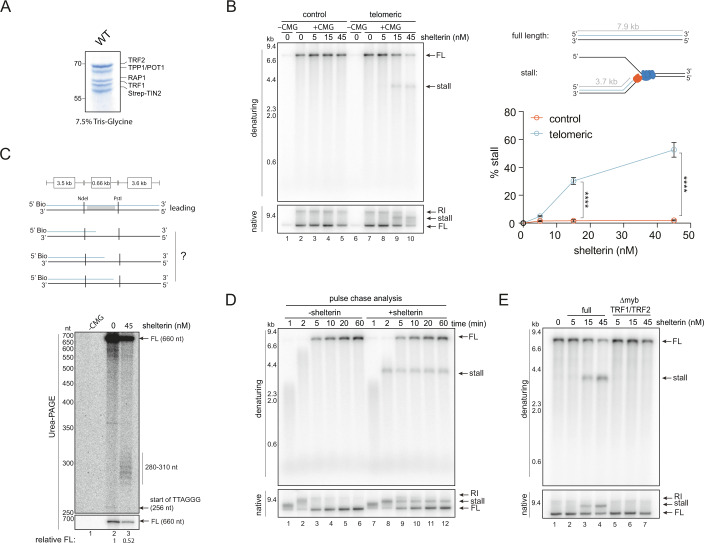
Shelterin can stall a human replisome on telomeric DNA. (**A**) 7.5% Tris-Glycine SDS-PAGE analysis of purified shelterin. See Fig. EV3 for characterisation of the complex and ‘methods’ for the isoforms and purification protocol used. (**B**) Replication reactions performed for 20 min on a template containing 60 TTAGGG repeats or a non-telomeric control in the presence of the indicated concentrations of shelterin were analysed by native and denaturing alkaline agarose electrophoresis. Position of the stall is indicated. Schematic showing expected leading strand products with and without stalling at the telomere. Graph shows quantification of the percentage forks stalled with and without shelterin. Signal intensities were normalised to product size to account for reduced labelling of stall products due their shorter length. Two-way ANOVA was used to determine statistical significance. Error bars show the S.E.M. *N* = 3 (three independent replication assays). Corresponding *P* values: 5 nM shelterin (0.72), 15 nM shelterin (1.95 × 10^7^), 45 nM shelterin (4.42 × 10^11^). (**C**) Replication reactions were performed for 20 min on a template containing 60 TTAGGG repeats in the presence or absence of 45 nM shelterin. Replication products isolated via a biotin moiety on the initiating oligonucleotide were digested with NdeI and PstI and analysed by denaturing urea polyacrylamide gel electrophoresis. See methods for details. Schematic shows fully replicated leading strand and theoretical stalled leading strand products. (**D**) Pulse chase analysis with a template containing 60 TTAGGG repeats in the presence or absence of shelterin. Chase containing an excess of non-radiolabelled dNTPs was added 50 s after initiation mix. Samples taken at the times indicated after initiation were analysed as in (**B**). See also the quantification in Fig. EV4A. (**E**) Products from replication reactions performed for 20 min on a template containing 60 TTAGGG repeats with the shelterin variants indicated were analysed as in B. ∆myb TRF1/TRF2 indicates shelterin in which the myb domains from both TRF1 and TRF2 have been deleted. See also, quantification in Figure EV5C. Source data are available online for this figure.

**Figure EV3:**
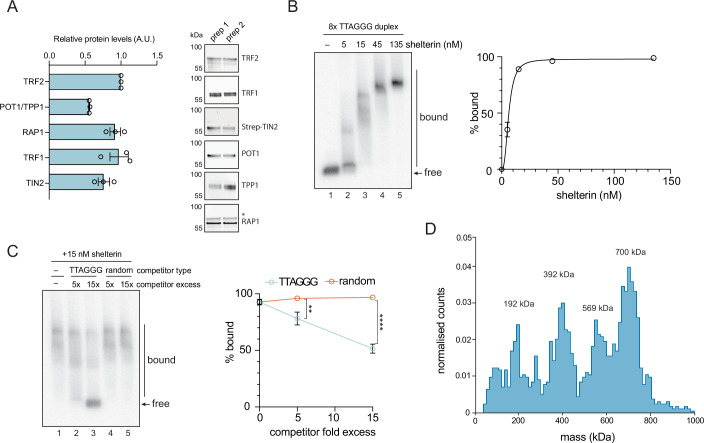
Supplementary data for Fig. 3. (**A**) Left side: quantification of the relative levels of each protein band of shelterin following SDS-PAGE as in Fig. 3A. Signal adjusted for protein molecular weight and presented relative to TRF2. Error bars show the S.E.M. *N* = 3 (three independent shelterin preparations). Right side: immunoblot of two independent shelterin preparations separated by 4–15% Tris-Glycine SDS-PAGE using the indicated antibodies. Asterisk marks a band deriving from additional detection of TRF2. (**B**) EMSA analysis of purified 6-membered shelterin binding to a 100 bp duplex containing 8x TTAGGG repeats at the indicated concentrations. Products separated by native agarose electrophoresis. Nonlinear regression (Hill equation) was used to fit the data. Kd = 6.24 nM. Error bars show the S.E.M. *N* = 3 (three independent EMSA). (**C**) EMSA analysis as in B was performed in the presence of 15 nM shelterin and a 5- or 15-times molar excess of unlabelled telomeric or control sequence. Products separated by native agarose electrophoresis. Two-way ANOVA was used to determine statistical significance. Error bars show the S.E.M. *N* = 3 (three independent EMSA). Corresponding *P* values: control vs telomeric 5× (0.0064), control vs telomeric 15× (1.19 × 106). (**D**) Mass photometry of shelterin with peak maxima indicated.

To examine the impact of shelterin in our system, we co-expressed TRF1, TRF2, TIN2, RAP1, TPP1 and POT1 in insect cells and purified a six-subunit complex via a strep tag on TIN2 (Figs. 3A and  EV3A). Electrophoretic mobility shift assays (EMSAs) confirmed purified shelterin bound tightly and specifically to telomeric DNA (Fig. EV3B,C) and mass photometry revealed a large complex of around 700 kDa in our preparations alongside several smaller species, in line with previous work (Zinder et al, 2022) (Fig. EV3D).

**Figure EV4:**
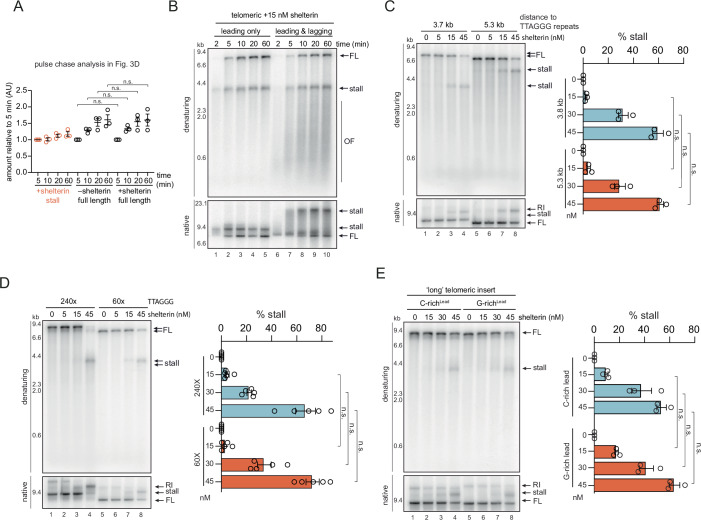
Supplementary data for Fig. 3. (**A**) Quantification of the relative intensities of telomere-stalled or full-length products over time after denaturing alkaline agarose as in Fig. 3B. Stall and FL signal was calculated relative to 5 min time point. Two-way ANOVA was used to determine statistical significance. Error bars show the S.E.M. *N* = 3 (three independent replication assays). (*P* = 0.99). (**B**) Replication reactions with leading (left) or leading and lagging strand synthesis (right) were performed with a template containing 60 TTAGGG repeats in presence of 15 nM shelterin for indicated times. Products were analysed by native and denaturing alkaline agarose electrophoresis as indicated. (**C**) Replication reactions were performed for 20 min with templates containing 60 TTAGGG repeats positioned 3.8 or 5.3 kb from the site of replication initiation with the indicated concentrations of shelterin. Products were analysed as in (**B**). Quantification of stall signal after denaturing alkaline agarose. Stall signal was calculated relative to no shelterin. Two-way ANOVA was used to determine statistical significance. Error bars show the S.E.M. *N* = 3 (three independent replication assays). Corresponding *P* values: 5 nM (0.9851), 15 nM (0.9324), 45 nM (0.9850). (**D**) Products from replication reactions performed for 20 min with telomeric template containing 240× or 60× repeats with the indicated concentrations of shelterin were analysed as in (**B**). Stall signal was calculated relative to no shelterin. Two-way ANOVA was used to determine statistical significance. Error bars show the S.E.M. *N* = 5 (five independent replication assays). Corresponding *P* values: 5 nM (0.9987), 15 nM (0.1803), 45 nM (0.9888). (**E**) Products from replication reactions performed for 20 min with templates containing 240 TTAGGG repeats oriented with C- or G-rich telomeric DNA as the leading strand template with the shelterin concentrations indicated were analysed as in B. Stall signal was calculated relative to no shelterin. Two-way ANOVA was used to determine statistical significance. Error bars show the S.E.M. *N* = 3 (three independent replication assays). Corresponding *P* values: 15 nM (0.5240), 30 nM (0.9501), 45 nM (0.3150).

Adding shelterin into reactions containing a template with 60 TTAGGG repeats resulted in pronounced stalling of leading strand synthesis at the position of telomeric DNA accompanied by a distinct band of replication intermediates after native agarose electrophoresis, suggesting replication forks had arrested at the telomere (Fig. 3B). To precisely map the position of this arrest, we digested replication products with restriction enzymes flanking the telomeric region and subjected them to urea polyacrylamide gel electrophoresis (urea-PAGE), revealing leading strands stalled within the first 30–50 bp of telomeric repeats (Fig. 3C). While these products could in principle derive from slowly migrating forks, we favour they are associated with terminally stalled replisomes because pulse-chase experiments following the fate of shelterin-arrested forks did not show evidence of shelterin bypass over 55 min (Figs. 3D and  EV4A).

**Figure EV5:**
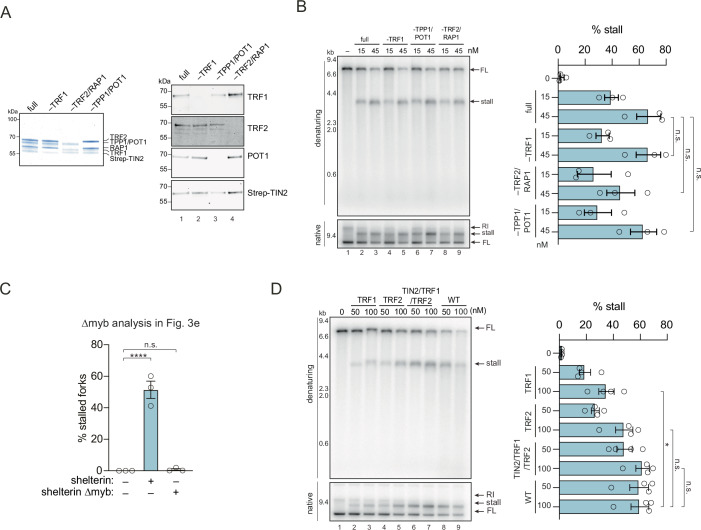
Supplementary data for Fig. 3. (**A**) Left side: 4–15% Tris-Glycine SDS-PAGE analysis of purified shelterin variants. Right side: Immunoblot of each purified shelterin variant with the antibodies indicated verifying loss of specific subunits expected. (**B**) Products from replication reactions performed for 20 min on a template containing 60 TTAGGG repeats in the presence or absence of shelterin or the shelterin variants indicated were analysed by native and denaturing alkaline agarose electrophoresis. One-way ANOVA was used to determine statistical significance. Error bars show the S.E.M. *N* = 3 (three independent replication assays). Corresponding *P* values: 45 nM full vs -TRF1 (0.9999), 45 nM full vs -TRF2/RAP1 (0.5519), 45 nM full vs -TPP1/POT1 (0.9997). (**C**) Quantification of Fig. 3E. Stall signal was calculated relative to no shelterin. One-way ANOVA was used to determine statistical significance. Error bars show the S.E.M. *N* = 3 (three independent replication assays). Corresponding *P* values: no shelterin vs full shelterin (5.07 × 10^5^), no shelterin vs shelterin ΔMyb (0.9806). (**D**) Products from replication reactions performed for 20 min on a template containing 60 TTAGGG repeats in the presence or absence of the indicated proteins were analysed as in (**B**). One-way ANOVA was used to determine statistical significance. Error bars show the S.E.M. *N* = *4* (four independent replication assays). Corresponding *P* values: 100 nM WT vs TRF1 (0.0155), 100 nM WT vs TRF2 (0.5131), 100 nM WT vs TRF1/TRF2/TIN2 (0.9999).

Examining the requirements for this effect showed that stalling was not affected by the presence or absence of lagging strand synthesis, the position of telomeric repeats on the template, increasing their length or changing their orientation (Fig. EV4B–E). Probing the contribution of different protein components revealed stalling was also not dependent on a particular shelterin subunit (Fig. EV5A, B) but required the myb domains of TRF1 or 2 (Fig. 3E and EV5C). As TRF1 and TRF2 alone or in complex with TIN2 induced a similar stalling effect (Fig. EV5D), these data support a model in which shelterin bound to telomeric DNA through either TRF1 or TRF2 has the potential to act as a roadblock for the human replisome.

### Shelterin induces large lagging strand gaps

**Figure 4:**
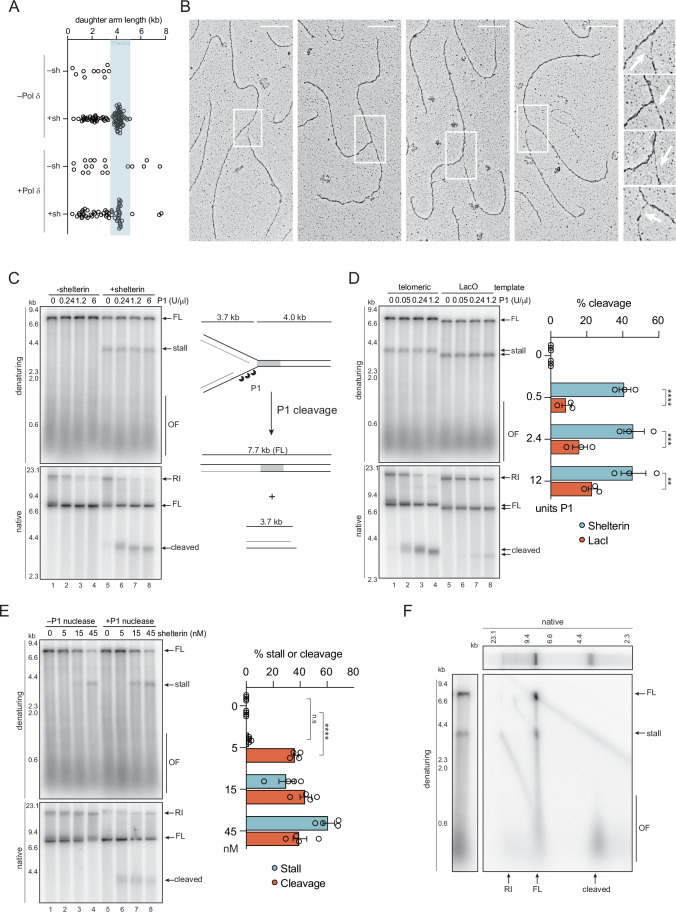
Shelterin induces large single-stranded gaps on the telomeric lagging strand. (**A**) Quantification of daughter arm lengths in the presence or absence of shelterin and Pol δ from Y-shaped DNA structures visualised by EM. Replication reactions were performed for 15 min on a template containing 60 TTAGGG repeats in the presence or absence of 15 nM shelterin before crosslinking and EM analysis. Y-shaped molecules from top to bottom: *n* = 10, *n *= 86, *n* = 18, *n* = 57. (**B**) Examples of Y-shaped molecules identified in the presence of shelterin. Inserts show enlargement of the fork junction revealing the presence of single-stranded DNA (white arrows). Scale bar represents 1 kb. (**C**) Replication reactions performed for 15 min on a template containing 60 TTAGGG repeats in the presence or absence of 15 nM shelterin. The amounts of P1 nuclease indicated were added 5 min before quenching. Products analysed by native and denaturing alkaline agarose electrophoresis as indicated. Schematic shows expected products after cleavage of gapped replication forks. (**D**) Products from replication reactions performed for 15 min on templates containing 60 TTAGGG repeats or an equivalent LacO array in the presence of 15 nM shelterin (lanes 1–4) or LacI (lanes 5–8) were separated as in (**C**). P1 nuclease was added as in (**C**). Percent P1 cleavage was calculated relative to reactions containing no P1. Two-way ANOVA was used to determine statistical significance. Error bars show the S.E.M. *N* = 3 (three independent replication assays). Corresponding *P* values: 0.5 U P1 (9.33 × 10^5^), 2.4 U P1 (0.00021), 12 U P1 (0.0033). (**E**) Products from replication reactions performed for 15 min on a template containing 60 TTAGGG repeats in the presence or absence of indicated shelterin concentrations were analysed as in (**C**). 0.24 U/μl P1 nuclease was added as in (**C**). Percent P1 cleavage was calculated relative to reactions containing no shelterin. Two-way ANOVA was used to determine statistical significance. Error bars show the S.E.M. *N* = 4 (four independent replication assays). Corresponding *P* values 0 nM vs 5 nM stall (0.99), 0 nM vs 5 nM P1 cleavage (9.30 × 10^7^). (**F**) Replication products from a 15 min reaction with a template containing 60 TTAGGG repeats and 15 nM shelterin were analysed by native agarose electrophoresis (top panel). In all, 1 U/μl P1 nuclease was added as in (**C**). The lane was excised and analysed by denaturing alkaline agarose electrophoresis (left hand panel). Source data are available online for this figure.

To examine the organisation of replication forks stalled by shelterin, products from replication reactions competent for leading and lagging strand synthesis were crosslinked with psoralen and UV and imaged by transmission electron microscopy (EM). We performed this analysis in the presence and absence of Pol δ to control for CMG-independent strand displacement synthesis events. Most visualised molecules were linear, likely because only a fraction was replicated. However, in the presence of shelterin we observed an increase in Y-shaped fork structures, many of which were enriched on telomeric DNA (Fig. 4A, Y-shaped: 7% vs 2% –Pol δ, 7% vs 3% Y-shaped + Pol δ).

**Figure EV6:**
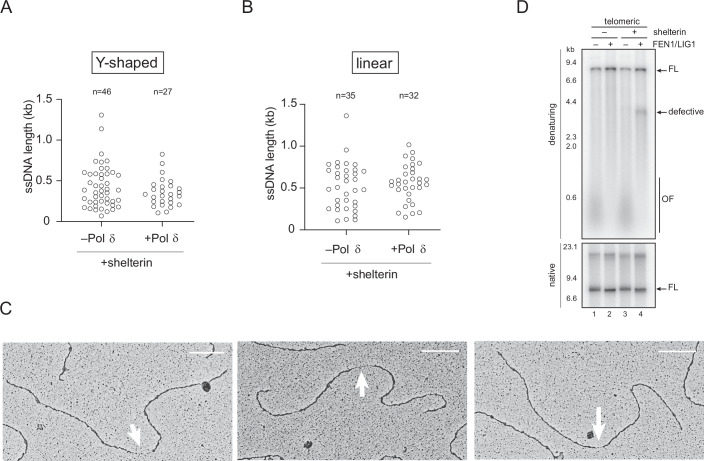
Supplementary data for Fig. 4. (**A**) Graph showing the length of single stranded gaps on Y-shaped fork structures measures by EM in the presence of shelterin and presence or absence of Pol δ as in Fig. 4B. (**B**) Graph showing the length of single stranded gaps measured on linear molecules in the presence of shelterin and presence or absence of Pol δ as shown in (**C**). (**C**) Electron micrographs showing linear DNA molecules with an internal tract of single stranded DNA at the position of telomeric DNA (white arrows) from reactions performed on a template containing 60 TTAGGG repeats in the presence of shelterin and absence of Pol δ. Scale bar represents 1 kb. (**D**) Replication reactions performed for 30 min on a template containing 60 TTAGGG repeats with shelterin and FEN1/LIG1 as indicated were analysed by native and denaturing agarose electrophoresis.

Closely examining these structures revealed most contained a tract of single stranded DNA (ssDNA) hundreds of nucleotides long on one arm of the fork junction (Figs. 4B and  EV6A, 66% –Pol δ, 73% +Pol δ). The presence of such large gaps was surprising because reconstituted budding yeast replisomes stalled by a heterologous protein barrier contained single-stranded regions around 30 nt (Westhorpe et al, 2024). To confirm the presence of these gaps, we digested replication products with the single-strand-specific nuclease, P1. Replication products from reactions containing shelterin yielded a distinct band 3.7 kb in length on native gels upon cleavage with P1, which matches the size expected if one arm had been cleaved off the stalled fork junction (Fig. 4C, bottom panel ‘cleaved’ product). Strikingly, at least 5–10 times more P1 was required to yield an equivalent product from forks stalled by the lac repressor, suggesting that such large gaps are specific to replication in the presence of shelterin and telomeric DNA (Fig. 4D, bottom panel, compare lanes 1–4 and 5–8).

To determine whether fork stalling was required for these gaps to form, we titrated down the concentration of shelterin. When reduced to a concentration of 5 nM, shelterin did not arrest DNA synthesis but still induced P1 cleavage (Fig. 4E, compare lanes 6 and 7) suggesting persistent stalling was not in fact required. Consistent with this idea, EM analysis revealed single-stranded telomeric gaps on molecules that were linear and likely to have been otherwise fully replicated (Fig. EV6B,C). Thus, shelterin-induced gaps can apparently persist, even when the replication fork proceeds past the telomere.

To determine whether these gaps arise on the leading or lagging strand, we excised a lane of P1-cleaved products from a native agarose gel and ran it in a second, denaturing dimension to reveal the types of nascent strands it contained. The P1-cleaved arm contained a prominent population of OFs but no corresponding leading strands, revealing shelterin induces gaps specifically on the lagging strand (Fig. 4F). Consistently, using the lagging strand maturation system characterised in Fig. 2, 60 telomeric repeats were not sufficient to inhibit lagging strand replication in the presence of FEN1 and LIG1 but we observed a prominent defect when 5 nM shelterin was also added (Fig. EV6D).

### Lagging strand gaps are induced by POT1 and can be partially suppressed by CST

**Figure 5:**
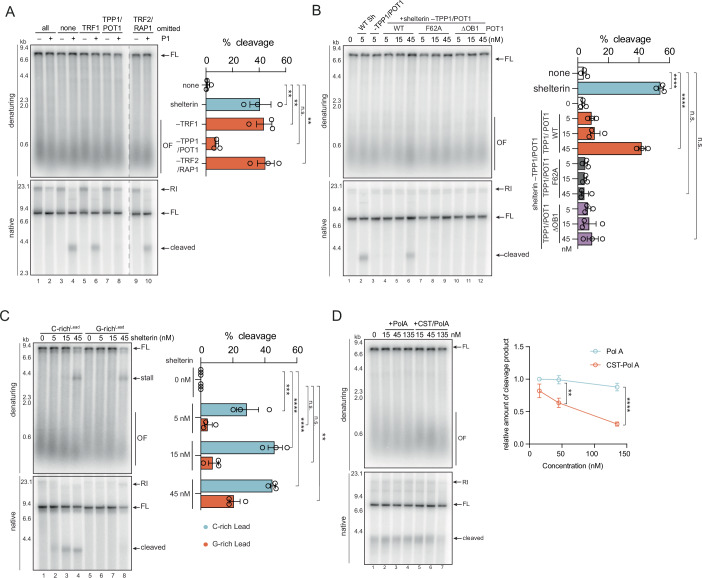
Lagging strand gaps are induced by POT1 binding to single-stranded G-rich DNA. (**A**) Replication reactions performed for 15 min on a template containing 60 TTAGGG repeats and with the shelterin variants indicated. 0.24 U/μl P1 nuclease was added 5 min prior to quenching and products analysed by native and denaturing alkaline agarose electrophoresis as indicated. Dotted line marks removal of an intervening lane. One-way ANOVA used to determine statistical significance. Error bars show the S.E.M. *N* = 3 (three independent replication assays). Corresponding *P* values: none vs shelterin (0.0012), none vs -TRF1 (0.0007), none vs -TPP1/POT1 (0.7968), none vs -TRF2/RAP1 (0.0006). (**B**) Products from replication reactions performed for 15 min on a template containing 60 TTAGGG repeats and with wild-type shelterin or 5 nM shelterin -TPP1/POT1 preincubated with the TPP1/POT1 variants indicated were analysed as in (**A**). In total, 0.24 U/μl P1 nuclease added as in (**A**). One-way ANOVA was used to determine statistical significance. Error bars show the S.E.M. *N* = 3 (three independent replication assays). Corresponding *P* values: none vs shelterin (6.52 × 10^13^), none vs 45 nM WT (2.51 × 10^10^), none vs 45 nM F62A (0.99), none vs 45 nM ΔOB1 (0.49). (**C**) Products from replication reactions performed for 15 min in the presence of the indicated shelterin concentrations and with templates containing 240 TTAGGG repeats oriented with the C- or G-rich sequence as the leading strand template were analysed as in (**A**). 0.24 U/μl P1 nuclease was added 5 min before quenching. Percent P1 cleavage was calculated relative to reactions containing no shelterin. Two-way ANOVA was used to determine statistical significance. Error bars show the S.E.M. *N* = 3 (three independent replication assays). Corresponding *P* values: C-rich Lead 0 vs 5 nM (6.37 × 10^5^), 0 vs 15 nM (1.88 × 10^7^), 0 vs 45 nM (2.79 × 10^7^), G-rich Lead 0 vs 5 nM (0.71), 0 vs 15 nM (0.34), 0 vs 45 nM (0.0017). (**D**) Products from replication reactions performed for 30 min on a template containing 60 TTAGGG repeats in the presence of 5 nM shelterin and the indicated concentrations of a complex of CST-pol α/prim or pol α/prim alone were analysed as in (**A**). 0.035 U/μl P1 nuclease added as in (**A**). Note in these reactions the concentration of RPA was reduced to 30 nM. See also Fig. EV7B. Level of P1 cleavage was relative to reactions containing 15 nM Pol α/prim. Two-way ANOVA was used to determine statistical significance. Error bars show the S.E.M. *N* = 4 (four independent replication assays. Corresponding *P* values: 45 nM (0.0031), 135 nM (0.000021). Source data are available online for this figure.

The data above reveal that shelterin can act as a potent inhibitor of DNA synthesis on the telomeric lagging strand. To define the mechanism underlying this effect, we repeated the P1 cleavage assay with shelterin complexes lacking specific subunits. Strikingly, TRF1 and TRF2/RAP1 were dispensable for P1 cleavage but TPP1/POT1 was not (Fig. 5A).

**Figure EV7:**
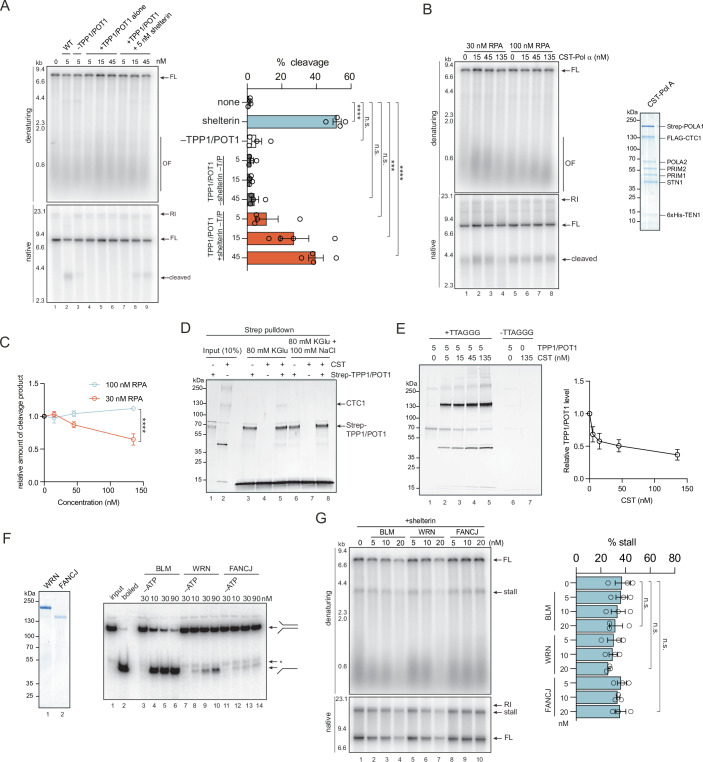
Supplementary data for Fig. 5. (**A**) Replication reactions performed for 15 min on a template containing 60 TTAGGG repeats with either wild-type shelterin, shelterin -TPP1/POT1, TPP1/POT1 alone or TPP1/POT1 with 5 nM shelterin -TPP1/POT1 added. 0.24 U/μl P1 nuclease was added 5 min before quenching and products were analysed by native and denaturing alkaline agarose electrophoresis. One-way ANOVA was used to determine statistical significance. Error bars show the S.E.M. *N* = *4* (four independent replication assays). Corresponding *P* values: none vs shelterin (1.45 × 10^8^), none vs -TPP1/POT1 (0.9791), none vs TPP1/POT1 alone 5 nM (0.9999), 15 nM (0.9999), 45 nM (0.9958), none vs TPP1/POT1 with shelterin -TPP1/POT1 5 nM (0.3891), 15 nM (0.0008), 45 nM (2.59 × 10^6^). (**B**) Right side: 4–15% Tris-Glycine SDS-PAGE analysis of purified CST-Pol α/prim complex. Left side: Replication reactions were performed for 15 min on a template containing 60 TTAGGG repeats with 5 nM shelterin and the indicated concentrations of CST-Pol α/prim complex and either 30 or 100 nM RPA. 0.035 U/μl P1 nuclease was added and products analysed as in (**A**). (**C**) Quantification of the level of P1 cleavage product from reactions as in (**B**). Level of P1 cleavage was relative to reactions containing no CST-Pol α/prim. Two-way ANOVA was used to determine statistical significance. Error bars show the S.E.M. *N* = 3 (three independent replication assays). *P* = 2.02 × 10^5^. (**D**) Pulldown assay with TPP1/POT1 precipitated via a strep tag on TPP1 in the presence of CST as indicated. Bound complexes were washed in buffer containing the indicated salts. Final complexes were separated by 4–15% Tris-Glycine SDS-PAGE before analysis by silver staining. (**E**) Pulldown assay with immobilised single stranded 3× TTAGGG repeats and the indicated concentrations of TPP1/POT1 and CST. Final complexes were separated by 4–15% Tris-Glycine SDS-PAGE before analysis by silver staining. The relative level of TPP1/POT1 is shown. *N* = 4 (four independent pulldown assays). Error bars show S.E.M. (**F**) Left side: 4–15% Tris-Glycine SDS-PAGE analysis of purified wild-type WRN and FANCJ. Right side: products from a helicase assay performed for 15 min on 1.5 nM duplexed substrate with the indicated concentrations of BLM, WRN or FANCJ in the presence of 10 nM RPA were analysed by native agarose electrophoresis. Asterisk indicates a non-specific band also in the input. (**G**) Products from replication reactions performed for 20 min on 60 TTAGGG telomeric templates presence of 15 nM shelterin with the indicated concentrations of helicases were analysed as in A. One-way ANOVA was used to determine statistical significance. Error bars show the S.E.M. *N* = 3 (three independent replication assays). Corresponding *P* values: 0 vs 20 nM BLM (0.9536), 0 vs 20 nM WRN (0.4048), 0 vs 20 nM FANCJ (0.9999).

A key function of POT1 is to prevent ATR activation at telomeres by binding tightly to single-stranded G-rich telomeric DNA (reviewed in (de Lange, 2026)). As the G-rich strand was the lagging strand template in our reactions, this led us to a simple model in which the inhibitory effect of shelterin resulted from the canonical ability of POT1 to bind ssDNA. To test this idea, we introduced the well-characterised F62A and ∆OB1 ssDNA binding mutations into POT1 (Lei et al, 2004; Wu et al, 2006). Both mutations prevented P1 cleavage (Fig. 5B). We also reversed the orientation of telomeric DNA so that the C-rich strand that cannot bind POT1 was the lagging strand template, upon which cleavage was also strongly reduced (Fig. 5C). Thus, binding of POT1 to G-rich ssDNA is responsible for the inhibitory effect on the lagging strand. Notably, TPP1/POT1 alone did not induce P1 cleavage unless the other subunits of shelterin were added (Fig. EV7A), suggesting the induction of replicative gaps required POT1 recruitment as part of shelterin - as is the case for its ATR-blocking role (Frescas and de Lange, 2014; Hockemeyer et al, 2007; Kibe et al, 2010; Takai et al, 2011).

While internal lagging strand gaps are not generally observed at telomeres in unperturbed human cells, they are reported without the Pol α/prim cofactor CST (Chen et al, 2013; Huang et al, 2012; Kasbek et al, 2013), which is recruited to telomeres through interactions with POT1 and G-rich single-stranded DNA (Cai et al, 2024; Carvalho Borges et al, 2023; Chen et al, 2012; Miyake et al, 2009; Wan et al, 2009). Adding a pre-formed complex of CST and Pol α/prim had no effect on the level of P1 cleavage under our standard reaction conditions (Fig. EV7B, lanes 5–8). However, when we also lowered the concentration of RPA to reduce competition for ssDNA, P1 cleavage was reduced (Figs. 5D and  EV7B, C). The relatively high CST concentrations required for this effect may indicate that the extent of P1 cleavage does not scale linearly with ssDNA length. However, consistent with recent work revealing phosphoregulation of the POT1:CST interaction (Cai et al, 2024), purified CST did not efficiently bind TPP1/POT1 in vitro and in fact competed it from single-stranded telomeric DNA (Fig. EV7D,E), suggesting additional regulatory factors may also be required.

## Discussion

**Figure 6:**
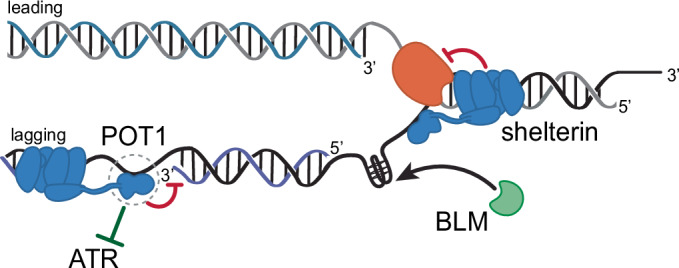
A model for the impact of shelterin and telomeric DNA on semiconservative DNA replication. See main text for details.

The cause of telomeric replication stress has been the focus of many studies that have revealed a range of properties that can perturb the replication process (Douglas, 2024; Maestroni et al, 2017). Yet, the pleiotropic effect of mutating many telomeric components, as well as the large number of factors bound to telomeres has meant that the direct impact of different components on the replisome has been challenging to examine using genetic approaches alone. Here, we have overcome this hurdle by using a reconstituted system for human DNA replication to reveal how telomeric DNA and the shelterin complex directly impact on different aspects of the replication process. Our study provides a molecular description of the challenges encountered by the replication fork as it navigates a core telomeric template (Fig. 6).

We show that G-rich telomeric DNA directly inhibits lagging strand replication by the human replisome. As lagging strand telomere replication is exquisitely sensitive to G4-stabilising agents and promoted by the G4-unwinding helicase BLM in vitro, we favour that this effect is primarily caused by G-quadruplexes assembled on the unwound lagging strand template, in line with cell-based studies implicating G4s in telomeric replication stress (PREPRINT: Matmati et al, 2025; Rizzo et al, 2009; Vannier et al, 2012). However, as G-rich telomeric DNA is inhibitory for Pol δ independently of G4s in primer extension assays (Lormand et al, 2013), other properties may feed into this effect. Leading strand replication is apparently much less sensitive to the same G-rich sequence even in the presence of G4 stabilising agents, consistent with the relatively low thermal stability of telomeric G4s (Tran et al, 2011; Williams et al, 2023) and a model in which coupled DNA unwinding and synthesis restrict interference from DNA secondary structures on the leading strand. Our data is consistent with a primary role for BLM at telomeres being to remove sequence-based structures on the lagging strand template, as suggested by previous genetic studies (Sfeir et al, 2009; Zimmermann et al, 2014). Notably, binding of BLM to TRF1 is required for this function in cells (Zimmermann et al, 2014) but is apparently dispensable in vitro, perhaps indicating that shelterin recruits BLM to telomeres and away from non-telomeric sites within the nucleus.

While telomeric sequence primarily inhibits the lagging strand, shelterin can stall synthesis entirely in vitro by acting as a replication roadblock. In a similar manner, TRF1 and TRF2 can stall the SV40 replicative helicase large T-antigen in cell extracts (Leman et al, 2012; Ohki and Ishikawa, 2004) and Rap1, the primary telomere binding protein in budding yeast, is a potent replication barrier in vitro and in vivo (Douglas and Diffley, 2021; Goto et al, 2015; Makovets et al, 2004). EM analysis indicates that most shelterin-stalled forks exhibit a classic Y-shape structure in vitro. However, as protein-stalled forks can act as a substrate for fork reversal (Kavlashvili et al, 2023) this may not be the case in vivo. Indeed, reversed forks have been detected at telomeres in yeast and mammalian cells (Huda et al, 2023; Margalef et al, 2018; Matmati et al, 2020), and a recent study suggests that slowing or stalling of the replisome by TRF1 leads to reversed forks that prevent telomere fragility in human cells (Vaurs et al, 2025). Whether shelterin-stalled forks are a direct substrate for reversal enzymes is an interesting question for the future.

We consider it likely that mechanisms are in place to prevent stalling at shelterin: in eukaryotic cells, protein-based replication blocks are overcome by helicase proteins working alongside the replisome, including budding yeast Rrm3 (Ivessa et al, 2003) and RTEL1 in mammals (Sparks et al, 2019). Rrm3 and RTEL1 are required for telomere replication in yeasts and humans respectively, and it is interesting to consider that in addition to its role unwinding G4s and t-loops (Vannier et al, 2012; Vannier et al, 2013), RTEL1 may restrict the extent to which replication forks stall at DNA-bound shelterin molecules. Challenges purifying active RTEL1 have prevented us from directly testing this idea, but we have found that other candidate helicases including BLM, WRN and FANCJ are unable to prevent the shelterin stall (Fig. EV7F,G). The second feature that may attenuate stalling is chromatin. TRF1 makes direct contact with histones and can remodel nucleosomes (Galati et al, 2006; Hu et al, 2023; Pisano et al, 2010), while binding of TRF2 to telomeric DNA is also affected by nucleosomes (Galati et al, 2015). How these effects modulate the impact of shelterin on replisome progression is an important topic for further study.

Crucially, shelterin is also a potent and specific inhibitor on the lagging strand, where binding of POT1 to single stranded G-rich DNA induces large unreplicated gaps. We consider this a secondary but highly penetrant consequence of the essential role of POT1 in binding ssDNA to prevent ATR activation at telomeres (Denchi and de Lange, 2007; Gong and de Lange, 2010). Based on the concentration of components in our assays, only a few POT1 molecules are required on each template molecule for this inhibition to take place. Which stage of lagging strand synthesis is blocked? With low shelterin concentrations, internal lagging strand gaps are left behind as the fork continues along the template, indicating that POT1 or a structure induced by POT1 prevents Pol δ from extending the next Okazaki fragment into the gap. We favour that POT1-containing shelterin complexes are left behind on the defective lagging strand as a persistent barrier, which may explain why POT1 is depleted from replication fork structures at telomeres (Lin et al, 2021). We do not rule out that POT1 also interferes with the priming process: OF priming is undertaken by Pol α/prim, which accesses the newly unwound template via an interaction with CMG (Jones et al, 2023). It is interesting to consider that POT1, and perhaps other sequence-specific ssDNA binding proteins at different regions of the genome, could compete with Pol α/prim to interfere with this process.

Considering how such POT1-induced gaps might be prevented in cells, the Pol α/prim cofactor and TPP1/POT1 binding partner CST is known to prevent replication defects and internal telomeric gaps on the G-rich strand (Carvalho Borges et al, 2023; Chen et al, 2013; Huang et al, 2012; Kasbek et al, 2013) and reduced the formation of replicative gaps in our system. Binding to CST could allow POT1 to fulfil its function preventing ATR activation while avoiding its inhibitory effect on the lagging strand. Given the relatively low efficiency with which CST executes this function in vitro, it will be interesting to examine how additional factors, for example, those regulating the CST-TPP1/POT1 interaction through phosphorylation (Cai et al, 2024) - influence this effect. We also consider that POT1 could be locally displaced by a helicase working in conjunction with pol δ.

## Methods

**Table:** Reagents and tools table

Reagent/resource	Reference or source	Identifier or catalog number
**Experimental models**		
*E. coli* 5-alpha competent (high efficiency)	New England Biolabs	C2987H
*E. coli* 10-beta	New England Biolabs	C3019H
E. coli BL21-CodonPlus (DE3)-RIL	Agilent Technology	230245
*E. coli* SURE2	Agilent Technology	200152
*Spodoptera frugiperda* (Sf9)	ThermoFisher Scientific	11496015
*Trichoplusia ni* (Hi5)	ThermoFisher Scientific	B85502
ySP53		
ySP54		
**Recombinant DNA**		
pBig1A_*MCM5_MCM6_MCM7_StrepII-PSF2_CDC45*	This study	N/A
pBig1B_*MCM2_MCM4_SLD5_PSF1_PSF3*	This study	N/A
pBig1C_*3xFLAG-MCM3*	This study	N/A
pACEBac1_*POLE1_3xFLAG-POLE2_POLE3_POLE4*	This study	N/A
pACEBac1_*3xFLAG-TIMELESS_TIPIN*	This study	N/A
pBig1a_*CLSPN-StrepII*	This study	N/A
pACEBac1_*StrepII-WDHD1*	This study	N/A
pACEBac1_*StrepII-RFC1*	This study	N/A
pACEBac1_*RFC2_RFC3*	This study	N/A
pACEBac1_*RFC4_RFC5*	This study	N/A
pACEBac1_*StrepII-CHTF18*	This study	N/A
pACEBac1_*CHTF8_DSCC1*	This study	N/A
pACEBac1_*POLA2*	This study	N/A
pACEBac1_*PRIM1*	This study	N/A
pACEBac1_*3xFLAG-CTC1_STN1_6xHis-TEN1*	This study	N/A
FEN1	This study	N/A
LIG1	This study	N/A
pACEBac1_pIDS_pIDC_*POLD1_POLD2_POLD3_6xHis-POLD4*	Lancey et al, 2020	N/A
pACEBac1_*StrepII-POLA1*	Baris et al, 2022	N/A
pACEBac1_*PRIM2*	Baris et al, 2022	N/A
p11d_*tRPA*	Gift from Marc Wold	Addgene 102613
pET16b_*PCNA*	Gift from Andrew Deans	Addgene 134898
pET28a_*Avitag-6xHis-PCNA*	This study	N/A
pFastBac1_*MBP-BLM-6xHis*	Pinto et al, 2016	N/A
pFastBac1_*MBP-BLM(K695A)-6xHis*	Pinto et al, 2016	N/A
pFastBac1_*MBP-WRN-6xHis*	Pinto et al, 2016	N/A
pBig1c_*FANCJ-StrepII*	This study	N/A
pD861_*lacI-6xHis*	Gift from John Diffley	N/A
pACEBac1_TERF1_StrepII-TINF2_TERF2_ACD_POT1_TERF2IP (full WT shelterin)	Gift from Sebastian Guettler	N/A
pACEBac1_ *StrepII-TINF2_TERF2_ACD_POT1_TERF2IP* (shelterin -TRF1)	Gift from Sebastian Guettler	N/A
pACEBac1_*TERF1_StrepII-TINF2_ACD_POT1* (shelterin -TRF2/RAP1)	Gift from Sebastian Guettler	N/A
pACEBac1_*TERF1_StrepII-TINF2_TERF2_TERF2IP* (shelterin -TPP1/POT1)	Gift from Sebastian Guettler	N/A
pACEBac1_*StrepII-TERF1*	Gift from Sebastian Guettler	N/A
pACEBac1_*StrepII-TERF2*	Gift from Sebastian Guettler	N/A
pACEBac1_*TERF1*	Gift from Sebastian Guettler	N/A
pACEBac1_*TERF2*	Gift from Sebastian Guettler	N/A
pACEBac1_StrepII-TINF2	Gift from Sebastian Guettler	N/A
pACEBac1_*TERF1(D375-431)_StrepII-TINF2_TERF2(D484-534)_ACD_POT1_TERF2IP* (shelterin TRF1 and TRF2 Myb deletion)	This study	N/A
pACEBac1_*POT1*	Gift from Sebastian Guettler	N/A
pACEBac1_*POT1(F62A)*	This study	N/A
pACEBac1_*POT1(*Δ*OB1)*	This study	N/A
pACEBac1_*StrepII-ACD* (long isoform of TPP1)	Gift from Sebastian Guettler	N/A
pACEBac1_*StrepII-ACD(*Δ*1-86)* (short isoform of TPP1)	This study	N/A
TBL95 (‘long’ telomeric)	This study	N/A
TBL105 (‘long’ control)	This study	N/A
TBL111 (‘short’ telomeric)	This study	N/A
TBL110 (‘short’ control)	This study	N/A
TBL130 (‘short’ telomeric for Fig. 3C)	This study	N/A
TBL122 (*lacO*)	This study	N/A
TBL93 (‘long’ control for Fig. EV1A,B)	This study	N/A
**Antibodies**		
Anti-TRF1		Custom made (gift from Sebastian Guettler)
Anti-TRF2	Cell Signalling	13136
Anti-RAP1	Bethyl Laboratories	A300-306A
Anti-POT1	Abcam	Ab124784
Anti-TPP1	Sigma	WH0065057M2
Anti-Strep	IBA Lifesciences	21507001
Anti-rabbit IgG	LI-COR	926-68071
Anti-mouse IgG	LI-COR	926-68070
Anti-rabbit IgG	LI-COR	926-32211
Anti-mouse IgG	LI-COR	926-32210
**Oligonucleotides and other sequence-based reagents**		
TBL237: Annealed fork, leading strand oligo, PstI site Phos-GATGTGGTAGGAAGTGAGAATTGGAGAGTGTGTTTTTTTTTTTTTTTTTTTTTTTTTTTTTTTTTTTTTTTTGAGGAAAGAATGTTGGTGAGGGTTGGGAAGTGGAAGGATGGGCTCGAGAGGTTTTTTTTTTTTTTTTTTTTTTTTTTTTT*T*T*T*T*T	Integrated DNA Technologies (PAGE purified)	N/A
TBL238: Annealed fork, lagging strand oligo, PstI site TTTTTTTTTTTTTTTTTTTTTTTTTTTTTTTTTTTTTTTTTTTTTTTTTTTTTTTTTTTTCACACTCTCCAATTCTCACTTCCTACCACATCTGCA	Integrated DNA Technologies (PAGE purified)	N/A
TBL272: Annealed fork, leading strand oligo, HindIII site Phos-AGCTTATGTGGTAGGAAGTGAGAATTGGAGAGTGTGTTTTTTTTTTTTTTTTTTTTTTTTTTTTTTTTTTTTTTTTGAGGAAAGAATGTTGGTGAGGGTTGGGAAGTGGAAGGATGGGCTCGAGAGGTTTTTTTTTTTTTTTTTTTTTTTTTTTTT*T*T*T*T*T	Integrated DNA Technologies (PAGE purified)	N/A
TBL273: Annealed fork, lagging strand oligo, HindIII site TTTTTTTTTTTTTTTTTTTTTTTTTTTTTTTTTTTTTTTTTTTTTTTTTTTTTTTTTTTTCACACTCTCCAATTCTCACTTCCTACCACATA	Integrated DNA Technologies (PAGE purified)	N/A
TBL363: Annealed fork, leading strand oligo, NotI site 5’PhosGGCCGCATGTGGTAGGAAGTGAGAATTGGAGAGTGTGTTTTTTTTTTTTTTTTTTTTTTTTTTTTTTTTTTTTTTTTGAGGAAAGAATGTTGGTGAGGGTTGGGAAGTGGAAGGATGGGCTCGAGAGGTTTTTTTTTTTTTTTTTTTTTTTTTTTTT*T*T*T*T*T	Integrated DNA Technologies (PAGE purified)	N/A
TBL363: Annealed fork, lagging strand oligo, NotI site TTTTTTTTTTTTTTTTTTTTTTTTTTTTTTTTTTTTTTTTTTTTTTTTTTTTTTTTTTTTCACACTCTCCAATTCTCACTTCCTACCACATGC	Integrated DNA Technologies (PAGE purified)	N/A
CLB391: Annealed fork, leading strand oligo, BsaI site 5’PhosACCGATGTGGTAGGAAGTGAGAATTGGAGAGTGTGTTTTTTTTTTTTTTTTTTTTTTTTTTTTTTTTTTTTTTTTGAGGAAAGAATGTTGGTGAGGGTTGGGAAGTGGAAGGATGGGCTCGAGAGGTTTTTTTTTTTTTTTTTTTTTTTTTTTTT*T*T*T*T*T	Integrated DNA Technologies (PAGE purified)	N/A
CLB392: Annealed fork, lagging strand oligo, BsaI site TTTTTTTTTTTTTTTTTTTTTTTTTTTTTTTTTTTTTTTTTTTTTTTTTTTTTTTTTTTTCACACTCTCCAATTCTCACTTCCTACCACAT	Integrated DNA Technologies (PAGE purified)	N/A
TBL253: Replication assay initiating oligo Bio-CCTCTCGAGCCCATCCTTCCACTTCCCAACCCTCACC	Integrated DNA Technologies	N/A
TBL296: Shelterin EMSA telomeric Bio-CGTCTATATTCTATTGTCTCTTAGGGTTAGGGTTAGGGTTAGGGTTAGGGTTAGGGTTAGGGTTAGGGTTAACATCAGTCTCACATAGATTAGCTCACGC	Integrated DNA Technologies (PAGE purified)	N/A
TBL297: Shelterin EMSA telomeric GCGTGAGCTAATCTATGTGAGACTGATGTTAACCCTAACCCTAACCCTAACCCTAACCCTAACCCTAACCCTAACCCTAAGAGACAATAGAATATAGACG	Integrated DNA Technologies (PAGE purified)	N/A
TBL337: Shelterin EMSA non-telomeric Bio-CGTCTATATTCTATTGTCTCATGCTAGCTTAGCGATACTAGCTAGCGTAGATCGATGCTAGATCGTAGTTAACATCAGTCTCACATAGATTAGCTCACGC	Integrated DNA Technologies (PAGE purified)	N/A
TBL338: Shelterin EMSA non-telomeric GCGTGAGCTAATCTATGTGAGACTGATGTTAACTACGATCTAGCATCGATCTACGCTAGCTAGTATCGCTAAGCTAGCATGAGACAATAGAATATAGACG	Integrated DNA Technologies (PAGE purified)	N/A
CLB353: BLM helicase assay top strand TTTTTTTTTTTTTTTTTTTTTTTTTTTTTTTTTTTTTTTTCGACCGTGCCAGCCTAAACCAGACTGCTACACACAGGATCGTTCGGTCTC	Williams et al, 2023. Integrated DNA Technologies (PAGE purified)	N/A
CLB354: BLM helicase assay bottom strand GAGACCGAACGATCCTGTGTGTAGCAGTCTGGTTTAGGCTGGCACGGTCGTTTTTTTTTTTTTTTTTTTTTTTTTTTTTTTTTTTTTTTT	Williams et al, 2023. Integrated DNA Technologies (PAGE purified)	N/A
CLB401: CST and TPP1/POT1 pulldown Bio-TTAGGGTTAGGGTTAGGGCGTCTATATTCTATTGTCTC	Integrated DNA Technologies	N/A
**Chemicals, enzymes and other reagents**		
EcoRI-HF	New England Biolabs	R3101
BamHI-HF	New England Biolabs	R3136
PstI-HF	New England Biolabs	R3140
EcoRV-HF	New England Biolabs	R3195
NheI-HF	New England Biolabs	R3131
SspI-HF	New England Biolabs	R3132
HindIII-HF	New England Biolabs	R3104
NotI-HF	New England Biolabs	R3189
BsaI-HF v2	New England Biolabs	R3733
Nb.BsrDI	New England Biolabs	R0648
NdeI	New England Biolabs	R0111
Quick CiP	New England Biolabs	M0525
T4 DNA Ligase	New England Biolabs	M0202
T4 PNK	New England Biolabs	M0201
Q5 Hot Start High-Fidelity DNA polymerase	New England Biolabs	M0493
Quick Ligase	New England Biolabs	M2200
SP Sepharose Fast flow	GE Healthcare	17-0729-10
Proteinase K	Millipore	10167980
phenol:chloroform:isoamyl alcohol (25:24:1)	Invitrogen	15593031
cOmplete EDTA-free protease inhibitor cocktail	Roche	11836170001
Leupeptin	Discovery Fine Chemicals	103476-89-7
AEBSF	Melford Labs	A20010
Pepstatin	Discovery Fine Chemicals	26305-03-3
Biotin	Discovery Fine Chemicals	58-85-5
3x FLAG peptide	Synthesised by BioServUK	N/A
Benzonase	Millipore	E1014
Prescission protease	GE Healthcare	27084301
[α-32 -P]-dCTP	Hartmann Analytic	SRP-205
[g-32 -P]-dATP	Hartmann Analytic	SRP-201
AMP-PNP	Bio-techne TOCRIS	6086
NTPs	ThermoFisher Scientific	R0481
dNTPs	ThermoFisher Scientific	10-297-018
Recombinant albumin	New England Biolabs	B9200
P1 nuclease	New England Biolabs	M0660
HindIII-digested phage lambda DNA	New England Biolabs	N3012
50 bp DNA ladder	New England Biolabs	N3236
SeaKem LE agarose	Lonza	50004
Trioxsalen	Sigma	T6137
Formamide	ThermoFisher Scientific	17899
Benzalkonium chloride	Sigma	B6285
Express Five SFM	ThermoFisher Scientific	10486025
Sf-900 II SFM	ThermoFisher Scientific	10902088
CMG	This study	N/A
Polymerase ε	This study	N/A
RPA	This study	N/A
Claspin	This study	N/A
Tipin-Timeless	This study	N/A
AND-1	This study	N/A
CTF18-RFC	This study	N/A
PCNA	This study	N/A
Polymerase α/primase	This study	N/A
Biotin-PCNA	This study	N/A
Polymerase δ	This study	N/A
RFC1-5	This study	N/A
FEN1	This study	N/A
LIG1	This study	N/A
BLM WT	This study	N/A
BLM K695A	This study	N/A
WRN	This study	N/A
FANCJ	This study	N/A
Shelterin WT	This study	N/A
Shelterin -TRF1	This study	N/A
Shelterin -TRF2/RAP1	This study	N/A
Shelterin -TPP1/POT1	This study	N/A
Shelterin TRF1 and TRF2 ΔMyb	This study	N/A
TRF1	This study	N/A
TRF2	This study	N/A
TIN2-TRF1-TRF2	This study	N/A
TPP1(long)/POT1 WT	This study	N/A
TPP1(long)/POT1 F62A	This study	N/A
TPP1(long)/POT1 ΔOB1	This study	N/A
TPP1(short)/POT1 WT	This study	N/A
CST	This study	N/A
CST-Pol α/prim complex	This study	N/A
**Software**		
Epson Scan 2 v6.7.64	Epson	https://www.epson.co.uk/en_GB/support/sc/epson-perfection-v800-photo/s/s1318?suggestionClicked=true
ImageJ(Fiji) v.2.14.0/1.54 f	National Institute of Health	https://imagej.net/ij/
Amersham Typhoon v3.0.0.2	Cytiva	https://www.cytivalifesciences.com/en/us/products/items/amersham-typhoon-gel-and-blot-imaging-systems-p-00192?psmenu=2
Image Studio v6.0	LI-COR	https://www.licorbio.com/image-studio
Adobe Photoshop 27.5.0	Adobe	https://www.adobe.com/uk/products/photoshop.html
Adobe Illustrator	Adobe	https://www.adobe.com/uk/products/illustrator.html
Prism10 v10.2.0	GraphPad	https://www.graphpad.com/updates
**Other**		
FLAG M2 affinity gel	Sigma	A2220
Streptactin XT superflow resin	IBA Lifesciences	2-5010-025
Amylose resin	New England Biolabs	E8021
Ni-NTA resin	QIAGEN	30210
Affi-Gel Blue resin	Bio-Rad	1537301
ssDNA cellulose	Cell Systems	LS01132
Heparin HiTrap column (1 and 5 mL)	Cytiva	17040601 and 17040701
HiTrap Q HP column	Cytiva	17115401
HisTrap HP column	Cytiva	17524701
Mono Q 5/50 GL column	Cytiva	17516601
Mono S 5/50 GL column	Cytiva	17516801
Superdex 200 Increase 10/300 GL column	Cytiva	17517501
Superose 6 Increase 10/300 GL column	Cytiva	29091596
HiLoad 16/600 Sephacryl S-200 HR column	Cytiva	28989335
Pierce High capacity NeutrAvidin agarose	ThermoFisher Scientific	29204
Microspin G-50 column	Cytiva	27533002
6X NEB Purple Loading Dye	New England Biolabs	B7024
Storage Phosphor Screen	GE Healthcare	BAS-IP MS 2025, GE28-9564-75
Whatman 3 mm chromatography paper	Cytiva	3030-917
Dynabeads M-280 Streptavidin	Invitrogen	11205D
MagStrep Streptactin XT beads	IBA Lifesciences	2-5090-002
Novex10% TBE	ThermoFisher Scientific	LC6678
4–15% Criterion TGX Precast Midi Gel	Bio-Rad	5671084
7.5% Mini-PROTEAN TGX Precase Gel	Bio-Rad	4561024
Nitrocellulose membrane	Amersham	10600003
Hi-Grade mica Grade V2	Ted Pella Inc	52-6
SilverQuest silver staining kit	Invitrogen	LC6070
Amersham Typhoon IPbiomolecular imagers	Cytiva	29187194
Owl Aluminium-Backed sequencers	ThermoFisher Scientific	S4S

### DNA construct assembly

For in vitro replication reactions, an 8.8 or 7.8 kb plasmid containing an insert of 1440 or 360 bp of telomeric DNA, respectively, was constructed from a pUC19 vector backbone. A corresponding control construct was also generated containing a fragment of non-telomeric control sequence. To generate the *lacO* template, a fragment containing four lac operator sequences interspersed with 11 bp of random sequence was excised from a plasmid (a kind gift from John Diffley) by restriction endonuclease digestion with EcoRI-HF (New England Biolabs, NEB) and BamHI-HF (NEB). This LacO fragment was inserted into the 7.8 kb control template digested with EcoRI-HF and BamHI-HF. The sequences of telomeric and non-telomeric replication templates used in the study are provided in the Appendix file.

For expression plasmid constructs, cDNAs encoding all subunits of CMG (MCM2, MCM3, MCM4, MCM5, MCM6, MCM7, CDC45, PSF2, PSF3, PSF5, SLD5), Polymerase ε (POLE1, POLE2, POLE3, POLE4), Tipin-Timeless, Claspin, AND-1, RFC1, RFC2-3, RFC4-5, CTF18, CTF8/DSCC1, POLA2, PRIM1, FANCJ, and CST were codon-optimised for expression in insect cells and synthesised by Twist Biosciences. The details of the affinity tagging strategy and expression system used for each factor are detailed in Reagents and Tools Table. Plasmids for the following constructs were acquired from external sources: Polymerase δ (four subunits, a kind gift from Dr Samir Hamdan)(Lancey et al, 2020), POLA1 and PRIM2 (a kind gift from Dr Joe Yeeles) (Baris et al, 2022), RPA (a kind gift from Dr Marc Wold)(Kelly et al, 1987), PCNA (a kind gift from Dr Andrew Deans) (Tan et al, 2020), BLM and WRN (a kind gift from Dr Petr Cejka) (Pinto et al, 2016) shelterin and variants (a kind gift from Prof. Sebastian Guettler) and Lac repressor (a kind gift from Dr John Diffley). Shelterin construct contains all six members in one plasmid and uses the long isoforms of TRF2 (542 amino acids), TPP1 (544 amino acids), and TIN2 (451 amino acids). The short TPP1 isoform and mutants for POT1, TRF1, and TRF2, were generated by PCR-based mutagenesis using starting plasmids that were a kind gift from Dr Oviya Inian and Prof. Sebastian Guettler. A construct for the generation of biotinylated PCNA was generated as previously described (Tehseen et al, 2019).

### Preparation of DNA templates for in vitro replication

Preparation of the telomeric plasmids was based on a protocol developed in the laboratory of Lars Nordenskiöld (Soman et al, 2022). Briefly, plasmids were transformed into SURE2 *Escherichia coli* cells grown at 30 °C. Colonies were inoculated onto LB-agar and an 8 ml small-scale culture supplemented with Ampicillin (50 μg/ml) and grown for 14–18 h. The presence of the telomeric insert was verified by restriction endonuclease digestion with EcoRI-HF and PstI-HF (NEB) and new small-scale cultures were inoculated from the streaked colonies and grown for 6–8 h before inoculating 10 L of bacteria with a verified clone. The presence of telomeric DNA in the final large-scale cultures was verified by restriction digestion. For all other DNA constructs used for in vitro replication reactions, plasmids were amplified using SURE2 *E.coli* cells grown at 30 °C. Plasmids were isolated by Plasmid Maxi or Giga kits (Qiagen).

Forked replication templates were generated as previously described (Georgescu et al, 2014), with minor modifications. Plasmid DNA (200 μg) was digested with 600 U of restriction enzyme (EcoRV-HF for telomeric templates containing 240 TTAGGG repeats, the corresponding control and the telomeric construct containing 60 TTAGGG repeats used in Fig. 3C. NheI-HF was used for telomeric templates containing 60 TTAGGG repeats, the corresponding control and *lacO* constructs, and SspI for the telomeric construct containing 60 TTAGGG repeats in Fig. EV4C) in a final volume of 800 μl for 2 h at 37 °C. QuickCIP (NEB) was added to the sample (150 U) and incubated for 1 h at 37 °C. Reactions were quenched by addition of 0.2% SDS, 25 mM EDTA pH 8.0, and 0.2 mg/ml Proteinase K and incubated for 20 min at 37 °C. DNA was extracted by adding an equal volume of phenol:chloroform:isoamyl alcohol (25:24:1) (Invitrogen, 15593031) and centrifuged (20,000× *g*, 5 min). The aqueous phase was collected, and the DNA precipitated by addition of NaCl to a final concentration of 300 mM and 2.5 volumes of −20 °C ethanol. Precipitated DNA was pelleted by centrifugation (20,000× *g*, 20 min, 4 °C). The pellet was washed with room temperature 70% ethanol (v/v), centrifuged (20,000× *g*, 5 min) and air-dried prior to resuspension in 200 μl of TE buffer (10 mM Tris-HCl pH 8.0, 1 mM EDTA). The resulting linearised DNA was subsequently digested with 600 U of restriction enzyme (NEB, HindIII-HF for 240 TTAGGG control and telomeric C-rich^Lead^ and 60 TTAGGG telomeric construct used in Fig. 3C, NotI-HF for 240 TTAGGG control and telomeric G-rich^Lead^, PstI-HF for 60 TTAGGG control, telomeric, and *lacO*, and BsaI for 60 TTAGGG telomeric construct used in Fig. EV4C) in 1× rCutSmart buffer (NEB, B6004) in a final volume of 800 μl for 3 h at 37 °C. The reaction was quenched, and the DNA extracted by phenol-chloroform followed by ethanol precipitation. The resulting pellet was resuspended in 100 μl TE buffer.

Oligonucleotides for generating the forked DNA end were adapted from a previously published system (Georgescu et al, 2014) (see Reagents and Tools Table for sequences). Oligonucleotides were heated to 95 °C for 5 min in TE buffer supplemented with 100 mM NaCl at a ratio of 3:1 and cooled to room temperature at a rate of −1 °C/min to facilitate annealing. A tenfold molar excess of the annealed fork was ligated to 60 μg of linearised template with 15,000 U T4 DNA Ligase (NEB) in 1× T4 DNA Ligase buffer (NEB) in a final volume of 600 μl and incubated for 2 h at 37 °C. The reaction was quenched and the DNA purified by phenol-chloroform extraction and ethanol precipitation, as above. The resulting pellet was resuspended in 100 μl of TE buffer. Template was digested with 150 U of restriction enzyme (EcoRV-HF for 240 TTAGGG control and telomeric constructs and 60 TTAGGG telomeric construct used for Fig. 3C, NheI-HF for 60 TTAGGG control, telomeric and *lacO* constructs, and SspI for 60 TTAGGG telomeric construct used in Fig. EV4C) in a final volume of 200 μl for 3 h at 37 °C. The reaction was quenched and the DNA purified by phenol-chloroform extraction and ethanol precipitation, as above. The resulting pellet was resuspended in 100 μl of TE buffer. Excess unligated fork was removed by size exclusion chromatography over a 10 ml bed volume of SP Sepharose Fast flow (GE Healthcare, 17-0729-10) equilibrated with TE + 100 mM NaCl with fractions analysed by agarose electrophoresis with SYBRGold. Peak fractions were pooled, supplemented with 500 mM NaCl and an equal volume of isopropanol and the DNA precipitated by centrifugation (20,000× *g*, 20 min), washed twice with 70% ethanol (v/v), air-dried and resuspended in TE to ~400–800 ng/μl.

### Protein expression

For expression in insect cells, plasmids were transposed into DH10MultiBac competent *E. coli* cells and grown in LB medium overnight at 37 °C. Bacmid DNA was extracted and used to transfect *Spodoptera frugiperda* (Sf9) insect cells. The virus was amplified in Sf9 cells grown at 27 °C, shaking at 130 rpm until passage 3 (P3), which was used to infect Hi5 suspension cultures at a density of 1–2 × 10^6^ cells/ml, grown at 27 °C with shaking at 130 rpm. The following proteins were expressed by co-infection with multiple viruses: CMG (MCM5-6-7-Psf2, MCM2-4-Sld5-Psf1-3, MCM3), RFC1-5 (RFC1, RFC2-3, RFC4-5), CTF18-RFC (CTF18, RFC2-3, RFC4-5, CTF8-DSCC1), Pol α/prim (POLA1, POLA2, PRIM1, PRIM2), POT1/Strep-TPP1, TRF1/TRF2/TIN2, CST- Pol α/prim co-complex (CST, POLA1, POLA2, PRIM1, PRIM2). Shelterin and shelterin variants were co-expressed from one plasmid. For polymerase δ expression, Sf9 cells were used. Cells were harvested 72 h post-infection by centrifugation (760× *g*, 10 min). Cell pellets were washed in PBS, transferred to 50 ml Falcon tubes, and pelleted again by centrifugation (1500× *g*, 10 min). Pellets were flash frozen in liquid nitrogen and stored at −80 °C.

For bacterial expression, plasmids transformed into *E. coli* strain BL21 (DE3) were grown at 37 °C with shaking at 180 rpm and induced with isopropyl β-D-thiogalactopyranoside (IPTG) as previously described for PCNA (Tan et al, 2020), RPA (Binz et al, 2006), Biotin-PCNA (Tehseen et al, 2019). Cells were harvested by centrifugation (3500× *g*, 20 min). Cell pellets were washed with PBS, transferred to 50 ml Falcon tubes, pelleted again by centrifugation (3500× *g*, 20 min). Supernatants were discarded, the pellets flash frozen in liquid nitrogen to be stored at −80 °C.

For expression in budding yeast, strains ySP53 or ySP54 were grown in YP + 2% raffinose at 30 °C, to a density of 2–3 × 10^7^ cells/ml. Galactose (2%) was added for 3 h to induce protein expression. Cells were collected by centrifugation, washed once in that protein’s lysis buffer without protease inhibitors and resuspended in 0.3–0.4 volumes of lysis buffer + protease inhibitors (Roche, 11836170001, PMSF, AEBSF and pepstatin A). The cell suspension was frozen dropwise in liquid nitrogen and the resulting yeast popcorn was crushed in a SPEX CertiPrep 6850 Freezer/Mill (3x 2 min cycles at a crushing rate 15) and the powder stored at −80 °C until required.

### Protein purification

#### CMG

CMG was purified as described (Jones et al, 2021).

#### Polymerase ε

Cells from 2 L of expression were resuspended in a 2× hypotonic lysis buffer (50 mM HEPES-KOH pH 7.6, 2 mM EDTA, 2 mM DTT) supplemented with protease inhibitors (cOmplete, EDTA-free). Cold glycerol was added to a final concentration of 16.67% from a 50% stock followed by cold KCl to a final concentration of 300 mM. The lysate was incubated for 30 min, 4 °C, stirring and insoluble material removed by centrifugation (35,000× *g*, 30 min, 4 °C). Solid ammonium sulphate (0.16 g/ml) was added to the lysate and incubated for 30 min, at 4 °C, stirring. Insoluble material was removed by centrifugation (35,000× *g*, 30 min, 4 °C). Additional solid ammonium sulphate (0.3 g/ml final) was added to the supernatant and incubated for 30 min, at 4 °C, stirring. Precipitated protein was collected by centrifugation (35,000× *g*, 30 min, 4 °C) and resuspended in buffer A (25 mM HEPES-KOH pH 7.6, 10% glycerol, 1 mM EDTA, 1 mM DTT, 0.005% NP-40). FLAG M2 affinity gel (2 ml) was added to the lysate and incubated for 2 h, 4 °C, rotating. Resin was collected in a 20 ml column and washed with 10 CV of buffer A + 300 mM KCl. Polymerase ε was eluted with 1 CV buffer A + 300 mM KCl + 0.2 mg/ml 3× FLAG peptide after incubating for 10 min, 2 CV buffer A + 300 mM KCl + 0.1 mg/ml 3× FLAG peptide after incubating for 5 min, and 3 CV of buffer A + 300 mM KCl. Eluates were pooled, diluted to 150 mM KCl, and applied to a 1 ml HiTrap Heparin HP column (GE Healthcare) equilibrated in buffer B (25 mM HEPES-KOH pH 7.6, 10% glycerol, 1 mM EDTA, 1 mM DTT, 0.01% NP-40) + 150 mM NaCl. The column was washed with 10 CV of buffer B + 150 mM NaCl and polymerase ε eluted over a 20 CV gradient from 150 mM to 1000 mM NaCl. Peak fractions were pooled, diluted to 150 mM NaCl, and applied to a 1 ml MonoQ 5/50 GL column (GE Healthcare) equilibrated in buffer B + 150 mM NaCl. The column was washed with 10 CV of buffer B + 150 mM NaCl and polymerase ε eluted over a 20 CV gradient from 150 mM to 1000 mM NaCl. Peak fractions were pooled and concentrated with an Amicon Ultra-15 30 kDa MWCO concentrator and applied onto a Superdex 200 Increase 10/300 GL column (GE Healthcare) equilibrated with buffer C (25 mM HEPES-KOH pH 7.6, 400 mM potassium L-glutamate, 10% glycerol, 1 mM EDTA, 1 mM DTT, 0.01% NP-40). Peak fractions were pooled and concentrated with an Amicon Ultra-4 30 kDa MWCO concentrator, aliquoted, frozen in liquid nitrogen, and stored at −80 °C.

#### RPA

RPA was purified as described with some minor alterations (Devbhandari et al, 2017). Cells from 4 L of expression were resuspended in lysis buffer (25 mM Tris-HCl pH 7.5, 1 M NaCl, 10% glycerol, 1 mM EDTA, 1 mM DTT) at 5 ml per 1 g of pellet supplemented with protease inhibitors (Leupeptin 10 μg/ml (Discovery fine chemicals), AEBSF 0.5 mM (Melford labs), Pepstatin 10 μg/ml (Discovery fine chemicals)). Lysozyme was added to 0.5 mg/ml and incubated on ice for 15 min. Cells were lysed via sonication (38%, 2 s on/4 s off, total 2 min) and insoluble material removed by centrifugation (14,000× *g*, 30 min, 4 °C). The lysate was diluted to 500 mM NaCl and incubated with Affi-Gel Blue (Bio-Rad, 1537301) resin (4 ml) for 1 h, 4 °C, rotating. Resin was collected, washed with 10 CV of buffer A (25 mM Tris-HCl pH 7.5, 10% glycerol, 1 mM EDTA, 1 mM DTT) + 500 mM NaCl and 5 CV of buffer A + 800 mM NaCl. RPA was eluted with 8 CV of buffer A + 2.5 M NaCl + 30% ethylene glycol and diluted immediately to 500 mM NaCl. Pooled eluates were incubated with hydrated and cleared ssDNA-cellulose (Cell Systems, LS01132) resin (1.5 ml) for 1 h, 4 °C, rotating. Resin was collected in a 20 ml column and washed with 10 CV of buffer A + 500 mM NaCl. RPA was eluted in 6 CV of buffer A + 1.5 M NaCl and 50% ethylene glycol and immediately diluted to 150 mM NaCl. Eluates were pooled and applied to a 1 ml MonoQ 5/50 GL column equilibrated in buffer A + 150 mM NaCl. The column was washed with 10 CV of buffer A + 150 mM NaCl and RPA eluted over a 30 CV gradient from 150 mM to 1000 mM NaCl. Peak fractions were pooled and concentrated by dialysis for 2–4 h against 1 L of dialysis buffer (25 mM Tris-HCl pH 7.5, 150 mM NaCl, 40% glycerol, 1 mM EDTA, 1 mM DTT). RPA was aliquoted, frozen in liquid nitrogen, and stored at −80 °C.

#### Claspin

Claspin was purified as described with some minor alterations (Jones et al, 2021). Cells from 1 L of expression were resuspended in a 2× hypotonic lysis buffer (100 mM Tris-HCl pH 8.0, 2 mM DTT) supplemented with protease inhibitors (cOmplete, EDTA-free). Cold glycerol was added to a final concentration of 16.67% from a 50% stock followed by cold NaCl to a final concentration of 400 mM. The lysate was incubated for 30 min, 4 °C, stirring and insoluble material removed by centrifugation (35,000× *g*, 30 min, 4 °C). The lysate was applied to 1 ml Streptactin XT superflow high-capacity resin and washed with 10 CV of buffer A (50 mM Tris-HCl pH 8.0, 10% glycerol, 1 mM DTT, 0.005% Tween-20) + 400 mM NaCl. The column was further washed with 10 CV of Buffer A + 400 mM NaCl and 5 mM Mg(OAc)_2_ + 0.5 mM ATP, and a further 10 CV wash with Buffer A + 400 mM NaCl. Claspin was eluted with 0.5 CV of buffer A + 400 mM NaCl and 30 mM biotin, followed by 1 CV after incubating for 10 min, and then 6 CV. Eluates were pooled, diluted to 100 mM NaCl and applied to a 1 ml MonoQ 5/50 GL column equilibrated in buffer A + 100 mM NaCl. The column was washed with 10 CV of buffer A + 100 mM NaCl and Claspin was eluted over a 20 CV gradient from 100 mM to 750 mM NaCl. Peak fractions were pooled and concentrated with an Amicon Ultra-15 30 kDa MWCO concentrator and applied onto a Superose 6 Increase 10/300 GL column (GE Healthcare) equilibrated with Buffer B (40 mM Tris-HCl pH 8.0, 150 mM NaCl, 10% glycerol, 1 mM DTT, 0.005% Tween-20). Peak fractions were pooled, concentrated in an Amicon Ultra-4 30 kDa MWCO concentrator, aliquoted, frozen in liquid nitrogen, and stored at −80 °C.

#### Tipin-timeless

Cells from 1 L of expression were resuspended in a 2× hypotonic lysis buffer (50 mM HEPES-KOH pH 7.6, 4 mM EDTA, 2 mM DTT) supplemented with protease inhibitors (cOmplete, EDTA-free). Cold glycerol was added to a final concentration of 16.67% from a 50% stock followed by cold NaCl to a final concentration of 300 mM. The lysate was incubated for 30 min, 4 °C, stirring and insoluble material removed by centrifugation (35,000× *g*, 30 min, 4 °C). FLAG M2 affinity gel (2 ml) was added to the lysate and incubated for 2 h, 4 °C, rotating. Resin was collected in a 20 ml column and washed with 15 CV of buffer A (25 mM HEPES-KOH pH 7.6, 10% glycerol, 1 mM EDTA, 1 mM DTT, 0.01% NP-40) + 300 mM NaCl. Tipin-Timeless was eluted with 1 CV buffer A + 300 mM NaCl + 0.2 mg/ml 3× FLAG peptide after incubating for 10 min, 2 CV buffer A + 300 mM NaCl + 0.1 mg/ml 3× FLAG peptide after incubating for 5 min, and 3 CV of buffer A + 300 mM NaCl. Eluates were pooled, diluted to 100 mM NaCl and applied to a 1 ml MonoQ 5/50 GL column equilibrated in buffer A + 100 mM NaCl. The column was washed with 10 CV of buffer A + 100 mM NaCl and Tipin-Timeless was eluted over a 20 CV gradient from 100 mM to 1000 mM NaCl. Peak fractions were pooled, diluted to 100 mM NaCl and applied to a 1 ml MonoS 5/50 GL column (GE Healthcare) equilibrated in buffer A + 100 mM NaCl. The column was washed with 10 CV of buffer A + 100 mM NaCl and Tipin-Timeless was eluted over a 20 CV gradient from 100 mM to 1000 mM NaCl. Peak fractions were pooled, concentrated, and buffer exchanged in buffer A + 100 mM NaCl in an Amicon Ultra-4 30 kDa MWCO concentrator. The concentrated protein was aliquoted, frozen in liquid nitrogen, and stored at −80 °C.

#### AND-1

AND-1 was purified as described with some minor alterations (Jones et al, 2021). Cells from 1 L of expression were resuspended in a 2× hypotonic lysis buffer (50 mM Tris-HCl pH 7.2, 2 mM DTT) supplemented with protease inhibitors (cOmplete, EDTA-free). Cold glycerol was added to a final concentration of 16.67% from a 50% stock followed by cold NaCl to a final concentration of 300 mM. The lysate was incubated for 30 min, 4 °C, stirring and insoluble material removed by centrifugation (35,000× *g*, 30 min, 4 °C). The lysate was applied to Streptactin XT superflow high-capacity resin (1 ml) in a 20 ml column and washed with 10 CV of buffer A (25 mM Tris-HCl pH 7.2, 10% glycerol, 1 mM DTT, 0.02% NP-40) + 300 mM NaCl. The column was further washed with 10 CV of Buffer A + 300 mM NaCl and 5 mM Mg(OAc)_2_ and 0.5 mM ATP, and a further 10 CV wash with Buffer A + 300 mM NaCl. AND-1 was eluted with 0.5 CV of buffer A + 300 mM NaCl + 30 mM biotin, followed by 1 CV after incubating for 10 min, and then 6 CV. Eluates were pooled, diluted to 150 mM NaCl and applied to a 1 ml MonoQ 5/50 GL column equilibrated in buffer A + 150 mM NaCl. The column was washed with 10 CV of buffer A + 150 mM NaCl and AND-1 was eluted over a 20 CV gradient from 150 mM to 1000 mM NaCl. Peak fractions were pooled and concentrated with an Amicon Ultra-15 30 kDa MWCO concentrator and applied onto a Superose 6 Increase 10/300 GL column equilibrated with Buffer B (25 mM Tris-HCl pH 7.2, 150 mM NaCl, 10% glycerol, 1 mM DTT, 0.02% NP-40). Peak fractions were pooled, concentrated in an Amicon Ultra-4 30 kDa MWCO concentrator, aliquoted, frozen in liquid nitrogen, and stored at −80 °C.

#### CTF18-RFC

Cells from 1 L of expression were resuspended in a 2× hypotonic lysis buffer (50 mM HEPES-KOH pH 7.6, 2 mM DTT) supplemented with protease inhibitors (cOmplete, EDTA-free). Cold glycerol was added to a final concentration of 16.67% from a 50% stock followed by cold NaCl to a final concentration of 300 mM. The lysate was incubated for 30 min, 4 °C, stirring and insoluble material removed by centrifugation (35,000× *g*, 30 min, 4 °C). The lysate was applied to Streptactin XT superflow high-capacity resin (1 ml) in a 20 ml column and washed with 20 CV of buffer A (25 mM HEPES-KOH pH 7.6, 10% glycerol, 1 mM EDTA, 1 mM DTT, 0.02% NP-40) + 300 mM NaCl. CTF18-RFC was eluted with 0.5 CV of buffer A + 300 mM NaCl + 30 mM biotin, followed by 1 CV after incubating for 10 min, and then 6 CV. Pooled eluates were diluted to 100 mM NaCl and incubated with hydrated and cleared ssDNA-cellulose resin (0.5 ml) for 1 h, 4 °C, rotating. Resin was collected in a 10 ml column and washed with 20 CV buffer A + 100 mM NaCl and CTF18-RFC was eluted with 10 CV buffer A + 300 mM NaCl. Eluates were pooled, diluted to 150 mM NaCl and applied to a 1 ml MonoQ 5/50 GL column equilibrated in buffer A + 150 mM NaCl. The column was washed with 10 CV of buffer A + 150 mM NaCl and CTF18-RFC was eluted over a 20 CV gradient from 150 mM to 1000 mM NaCl. Peak fractions were pooled, concentrated, and buffer exchanged in buffer B (25 mM HEPES-KOH pH 7.6, 300 mM KOAc, 10% glycerol, 1 mM DTT, 0.005% Tween-20) in an Amicon Ultra-4 30 kDa MWCO concentrator. The concentrated protein was aliquoted, frozen in liquid nitrogen, and stored at −80 °C.

#### PCNA

PCNA was purified as described with some minor alterations (Wilson et al, 2017). Cells from 1 L of expression were resuspended in lysis buffer (50 mM Tris-HCl pH 8.0, 100 mM NaCl, 10% glycerol, 1 mM EDTA) at 5 ml per 1 g of pellet supplemented with protease inhibitors (Leupeptin 10 μg/ml, AEBSF 0.5 mM, Pepstatin 10 μg/ml). Lysozyme was added to 0.5 mg/ml and incubated on ice for 15 min. Cells were lysed via sonication (38%, 2 s on/4 s off, total 2 min) and insoluble material removed by centrifugation (14,000× *g*, 30 min, 4 °C). Filtered lysate was applied to a 5 ml HiTrap Q HP column (GE Healthcare) equilibrated with Buffer A (20 mM Tris-HCl pH 8.0, 10% glycerol) + 100 mM NaCl. The column was washed with 15 CV of buffer A + 100 mM NaCl. PCNA was eluted over a 5 CV gradient from 100 mM to 300 mM NaCl, followed by 3 CV at 300 mM NaCl, then 3 CV from 300 mM to 600 mM NaCl, then 5 CV from 600 to 700 mM NaCl, and 5 CV from 700 mM to 1000 mM NaCl. Peak fractions were pooled and dialysed for 2 h against 2 L of dialysis buffer (30 mM NaOAc pH 5.0, 5 mM NaCl, 10% glycerol). PCNA was applied to a 5 ml HiTrap Heparin HP column (GE Healthcare) equilibrated with Buffer B (30 mM NaOAc pH 5.0, 10% glycerol) + 5 mM NaCl. The column was washed with 5 CV of buffer B + 5 mM NaCl. PCNA was eluted over a 10 CV gradient from 5 mM to 1000 mM NaCl. Peak fractions were pooled and concentrated with an Amicon Ultra-15 10 kDa MWCO concentrator and applied onto a HiLoad 16/600 Sephacryl S-200 HR column (GE Healthcare) equilibrated with buffer C (25 mM Tris-HCl pH 7.5, 25 mM NaCl, 10% glycerol, 0.5 mM EDTA). Peak fractions were pooled and concentrated with an Amicon Ultra-4 10 kDa MWCO concentrator aliquoted into 500 μl fractions, frozen in liquid nitrogen, and stored at −80 °C. A 500 μl aliquot was then applied to a 1 ml MonoQ 5/50 GL column (GE Healthcare) equilibrated in buffer A + 100 mM NaCl. The column was washed with 10 CV of buffer A + 100 mM NaCl and PCNA eluted over a 30 CV gradient from 100 mM to 1000 mM NaCl. Peak fractions were pooled, concentrated, and buffer exchanged in buffer C in an Amicon Ultra-4 10 kDa MWCO concentrator. The concentrated protein was aliquoted, frozen in liquid nitrogen, and stored at −80 °C.

#### Polymerase α/prim

Pol α/prim was purified described with some minor adjustments (Baris et al, 2022). Cells from 1 L of expression were resuspended in a 2× hypotonic lysis buffer (50 mM Tris-HCl pH 7.2, 2 mM DTT) supplemented with protease inhibitors (cOmplete, EDTA-free). Cold glycerol was added to a final concentration of 16.67% from a 50% stock followed by cold NaCl to a final concentration of 300 mM. The lysate was incubated for 30 min, 4 °C, stirring and insoluble material removed by centrifugation (35,000× *g*, 30 min, 4 °C). The lysate was applied to Streptactin XT superflow high-capacity resin (3 ml) in a 20 ml column and washed with 10 CV of buffer A (25 mM Tris-HCl pH 7.2, 10% glycerol, 1 mM DTT, 0.02% NP-40) + 300 mM NaCl and 10 CV of buffer A + 100 mM NaCl. Pol α/prim was eluted with 0.5 CV of buffer A + 100 mM NaCl + 30 mM biotin, followed by 1 CV after incubating for 10 min, and then 6 CV. Pooled eluates were applied to a 1 ml MonoQ 5/50 GL column equilibrated in buffer A + 100 mM NaCl. The column was washed with 10 CV of buffer A + 100 mM NaCl and polymerase α was eluted over a 20 CV gradient from 100 mM to 1000 mM NaCl. Peak fractions were pooled and concentrated with an Amicon Ultra-15 30 kDa MWCO concentrator and applied onto a Superdex 200 Increase 10/300 GL column equilibrated with Buffer B (25 mM Tris-HCl pH 7.2, 150 mM NaCl, 10% glycerol, 1 mM DTT, 0.005% Tween-20). Peak fractions were pooled, concentrated in an Amicon Ultra-4 30 kDa MWCO concentrator, aliquoted, frozen in liquid nitrogen, and stored at −80 °C.

#### Biotin-PCNA agarose

Biotin-PCNA was purified as described with minor alterations (Raducanu et al, 2020). Cells from 1 L of expression were resuspended in lysis buffer (50 mM Tris-HCl pH 7.5, 80 mM NaCl, 5% glycerol, 1 mM EDTA, 10 mM 2-mercaptoethanol, 1% NP-40, 20 mM imidazole pH 7.5) at 5 ml per 1 g of pellet supplemented with protease inhibitors (Leupeptin 10 μg/ml, AEBSF 0.5 mM, Pepstatin 10μg/ml). Lysozyme was added to 2 mg/ml and incubated on ice for 15 min. Cells were lysed via sonication (38%, 2 s on/4 s off, total 2 min) and insoluble material removed by centrifugation (14,000× *g*, 30 min, 4 °C). The lysate was applied onto a 5 ml HisTrap HP column (GE Healthcare) equilibrated with Buffer A (50 mM Tris-HCl pH 7.5, 500 mM NaCl, 5% glycerol, 10 mM β-mercaptoethanol) + 20 mM imidazole pH 7.5. The column was washed with 15 CV of buffer A + 20 mM imidazole pH 7.5 and Biotin-PCNA was eluted over a 15 CV gradient from 20 mM to 500 mM imidazole pH 7.5. Peak fractions were pooled and aliquoted in 4 ml tubes, frozen in liquid nitrogen, and stored at −80 °C. To make the biotin-PCNA Agarose column, 8 mg of protein was incubated with 2 ml of Neutravidin beads (Fisher Scientific, 11805845) for 2 h, 4 °C, rotating. Resin was collected in a 20 ml column and washed with 15 CV of buffer A. Resin was resuspended in 1 CV of buffer A to generate the PCNA-agarose resin for use in polymerase δ purification.

#### Polymerase δ

Polymerase δ was purified as described (Tehseen et al, 2019) with minor adjustments. Cells from 2 L of expression were resuspended in lysis buffer (50 mM Tris-HCl pH 7.5, 500 mM NaCl, 10% glycerol, 5 mM β-mercaptoethanol, 0.2% NP-40) supplemented with protease inhibitors (cOmplete, EDTA-free) and 10 U/ml Benzonase (Millipore, E1014) at 3-5 ml per 1 g of pellet. Cells were lysed via sonication (30%, 2 s on/6 s off, total 2 × 3 min) and insoluble material removed by ultracentrifugation (150,000× *g*, 1 h, 4 °C). The lysate was adjusted to 40 mM imidazole pH 7.5. The lysate was applied onto a 5 ml HisTrap HP column equilibrated with Buffer A (50 mM Tris-HCl pH 7.5, 80 mM NaCl, 10% glycerol, 5 mM β-mercaptoethanol) + 40 mM imidazole pH 7.5. The column was washed with 10 CV of buffer A + 40 mM imidazole pH 7.5 and polymerase δ eluted over a 10 CV gradient from 40 mM to 500 mM imidazole pH 7.5. Peak fractions were pooled and incubated with PCNA-Agarose resin (1 ml of resin per 5 ml of eluate) pre-equilibrated with buffer B (50 mM Tris-HCl pH 7.5, 5% glycerol, 10 mM β-mercaptoethanol) + 100 mM NaCl overnight at 4 °C, rotating. Resin was collected in a 20 ml column and washed with 10 CV of buffer B + 100 mM NaCl and 10 CV of buffer B + 200 mM NaCl. Proteins were eluted with 10 CV of buffer B + 1.2 M NaCl. Pooled eluates were concentrated with a Vivaspin6 10 kDa MWCO concentrator and applied onto a Superdex 200 Increase 10/300 GL column equilibrated with Buffer C (50 mM Tris-HCl pH 7.5, 150 mM NaCl, 5% glycerol, 1 mM DTT). Peak fractions were pooled, concentrated Vivaspin6 10 kDa MWCO concentrator aliquoted, frozen in liquid nitrogen, and stored at −80 °C.

#### RFC1-5

Cells from 1 L of expression were resuspended in a 2× hypotonic lysis buffer (50 mM HEPES-KOH pH 7.6, 2 mM DTT) supplemented with protease inhibitors (cOmplete, EDTA-free). Cold glycerol was added to a final concentration of 16.67% from a 50% stock followed by cold NaCl to a final concentration of 300 mM. The lysate was incubated for 30 min, 4 °C, stirring and insoluble material removed by centrifugation (35,000× *g*, 30 min, 4 °C). The lysate was applied to Streptactin XT superflow high-capacity resin (1 ml) in a 20 ml column and washed with 20 CV of buffer A (25 mM HEPES-KOH pH 7.6, 10% glycerol, 1 mM EDTA, 1 mM DTT, 0.02% NP-40) + 300 mM NaCl. RFC1-5 was eluted with 0.5 CV of buffer A + 300 mM NaCl + 30 mM biotin, followed by 1 CV after incubating for 10 min, and then 6 CV. Pooled eluates were incubated with hydrated and cleared ssDNA-cellulose resin (0.5 ml) for 1 h, 4 °C, rotating. Resin was collected in a 10 ml column and washed with 20 CV buffer A + 300 mM NaCl and RFC1-5 was eluted with 10 CV buffer A + 500 mM NaCl. Eluates were pooled, diluted to 150 mM NaCl and applied to a 1 ml MonoQ 5/50 GL column equilibrated in buffer A + 150 mM NaCl. The column was washed with 10 CV of buffer A + 150 mM NaCl and RFC1-5 was eluted over a 20 CV gradient from 150 mM to 1000 mM NaCl. Peak fractions were pooled, concentrated, and buffer exchanged in buffer B (25 mM HEPES-KOH pH 7.6, 300 mM KOAc, 10% glycerol, 1 mM DTT, 0.005% Tween-20) in an Amicon Ultra-4 30 kDa MWCO concentrator. The concentrated protein was aliquoted, frozen in liquid nitrogen, and stored at −80 °C.

#### FEN1 and LIG1

Frozen cell powder (from 12 l ySP53 culture) was thawed and resuspended in lysis buffer (25 mM Tris-Cl pH 7.2, 500 mM NaCl, 10% glycerol, 1 mM DTT, 0.02% NP40) supplemented with protease inhibitors (cOmplete, EDTA-free). Insoluble material was removed by centrifugation (235,000× *g*, 4 °C, 1 h). FLAG M2 affinity gel (2 ml) was added to the lysate and incubated for 1.5 h, 4 °C, rotating. The resin was collected in a disposable column and washed with 50 CV lysis buffer followed by 10 CV lysis buffer + 5 mM MgOAc + 1 mM ATP and then another 10 CV lysis buffer. FEN1 was eluted with 1 CV lysis buffer + 0.5 mg/ml 3× FLAG peptide after incubating for 30 min, 1 CV lysis buffer + 0.25 mg/ml 3× FLAG peptide. Pooled eluates were concentrated with a Amicon Ultra-15 10 KDa MWCO concentrator and then applied onto a Superdex 200 Increase 10/300 GL column (GE Healthcare) equilibrated in buffer A (25 mM Tris-Cl pH 7.2, 300 mM NaOAc, 10% glycerol, 1 mM DTT, 0.02% NP40). Peak fractions were pooled, concentrated in a Amicon Ultra-15 10 KDa MWCO concentrator, aliquoted, frozen in liquid nitrogen, and stored at −80 °C. LIG1 was purified from strain ySP54 in the same way except that HEPES-KOH pH 7.6 was used in place of Tris-Cl pH 7.2 in the lysis and gel filtration buffers.

#### Wild-type and helicase inactive BLM and wild-type WRN

BLM/WRN was purified as described with some minor alterations (Pinto et al, 2016). Cells from 1 L of expression were resuspended in a 2× hypotonic lysis buffer (100 mM Tris-HCl pH 7.5, 2 mM EDTA, 2 mM DTT) supplemented with protease inhibitors (cOmplete, EDTA-free). Cold glycerol was added to a final concentration of 16.67% from a 50% stock followed by cold NaCl to a final concentration of 300 mM. The lysate was incubated for 30 min, 4 °C, stirring and insoluble material removed by centrifugation (35,000× *g*, 30 min, 4 °C). Amylose resin (NEB, E8021) (3 ml) was added to the lysate and incubated for 1 h, 4 °C, rotating. Resin was collected in a 20 ml column and washed with 15 CV of buffer A (50 mM Tris-HCl pH 7.5, 10% glycerol, 1 mM DTT) + 1 M NaCl. BLM/WRN was eluted with 10 CV of buffer A + 300 mM NaCl + 10 mM maltose. Eluates were pooled and incubated with 250 nM PreScission protease (GE Healthcare, 27084301) for 1 h, 4 °C, rotating. Eluates were then supplemented with 10 mM imidazole pH 7.5 before incubating with Ni-NTA resin (QIAGEN, 30210) (1 ml) for 1 h, 4 °C, rotating. Resin was collected in a 20 ml column and washed with 20 CV of buffer A + 1 M NaCl + 58 mM imidazole pH 7.5 followed by 5 CV of buffer A + 150 mM NaCl + 58 mM imidazole pH 7.5. BLM/WRN was eluted with 10 CV of buffer A + 100 mM NaCl + 300 mM imidazole pH 7.5. Eluates were pooled and concentrated by dialysis for 2 h against 1 L of buffer B (50 mM Tris-HCl pH 7.5, 100 mM NaCl 40% glycerol, 1 mM DTT). BLM/WRN was aliquoted, frozen in liquid nitrogen, and stored at −80 °C.

#### FANCJ

FANCJ was purified as described (Yaneva et al, 2023) with minor adjustments. Cells from 2 L of expression were resuspended in lysis buffer (50 mM Tris-HCl pH 8, 150 mM KCl, 10% glycerol, 1 mM DTT, 0.01% Triton X-100, 10 mM MgCl_2_) supplemented with protease inhibitors (cOmplete, EDTA-free) and 25 U/ml Benzonase (Millipore, E1014) at 3–5 ml per 1 g of pellet and incubated on ice for 30 min. Cells were lysed by Dounce homogenisation and insoluble material removed by centrifugation (41,000× *g*, 30 min, 4 °C). The lysate was applied to Streptactin XT superflow high-capacity resin (2 ml) in a 20 ml column and washed with 30 CV of buffer A (50 mM Tris-HCl pH 8.0, 500 mM KCl, 10% glycerol, 1 mM DTT, 0.01% Triton X-100, 10 mM MgCl_2_). FANCJ was eluted with 0.5 CV of buffer A + 20 mM biotin, followed by 1 CV after incubating for 10 min, and then 6 CV. Eluates were pooled, diluted to 150 mM KCl, and applied to a 1 ml HiTrap Heparin HP column (GE Healthcare) equilibrated in buffer B (50 mM Tris-HCl pH 8.0, 10% glycerol, 1 mM DTT) + 150 mM KCl. The column was washed with 10 CV of buffer B + 150 mM KCl. FANCJ was eluted with 5 CV at 350 mM KCl, followed by 5 CV at 450 mM KCl and finally 10 CV gradient from 450 mM KCl to 1150 mM KCl. Pooled eluates were concentrated with a Vivaspin6 30 kDa MWCO concentrator and applied onto a Superdex 200 Increase 10/300 GL column equilibrated with Buffer C (50 mM HEPES-KOH pH 7.5, 300 mM KCl, 10% glycerol, 1 mM DTT). Peak fractions were pooled, concentrated Vivaspin6 30 kDa MWCO concentrator aliquoted, frozen in liquid nitrogen, and stored at −80 °C.

#### Shelterin wild-type and variants

Shelterin and shelterin variants, shelterin ∆Myb, and TRF1-TRF2-TIN2 were purified as described for the full complex (Eickhoff et al, 2025) via a Strep-tag on TIN2, with some minor modifications. Cells from 1 L of expression were resuspended in a 2× hypotonic lysis buffer (100 mM HEPES-KOH pH 7.6, 2 mM DTT) supplemented with protease inhibitors (cOmplete, EDTA-free). Cold glycerol was added to a final concentration of 16.67% from a 50% stock followed by cold NaCl to a final concentration of 300 mM. The lysate was incubated for 30 min, 4 °C, stirring and insoluble material removed by centrifugation (35,000× *g*, 30 min, 4 °C). The lysate was applied to Streptactin XT superflow high-capacity resin (1 ml) in a 20 ml column and washed with 30 CV of buffer A (50 mM HEPES-KOH pH 7.6, 300 mM NaCl, 10% glycerol, 1 mM DTT). Shelterin was eluted with 0.5 CV of buffer A + 30 mM biotin, followed by 1 CV after incubating for 10 min, and then 6 CV. The most concentrated eluate was applied onto a Superose 6 Increase 10/300 GL column equilibrated with Buffer A except for TRF1, TRF2, and TPP1/POT1 complexes, which were run over a Superdex 200 Increase 10/300 GL column (GE healthcare) equilibrated with Buffer A. Peak fractions were pooled, concentrated in a Vivaspin6 10 kDa MWCO concentrator, aliquoted, frozen in liquid nitrogen, and stored at −80 °C.

#### CST

CST was purified as described (Lim et al, 2020) with some minor alterations. Cells from 1 L of expression were resuspended in a 2X hypotonic lysis buffer (100 mM HEPES-KOH pH 7.6, 2 mM DTT) supplemented with protease inhibitors (cOmplete, EDTA-free). Cold glycerol was added to a final concentration of 16.67% from a 50% stock followed by cold NaCl to a final concentration of 300 mM. The lysate was incubated for 30 min, 4 °C, stirring and insoluble material removed by centrifugation (35,000× *g*, 30 min, 4 °C). FLAG M2 affinity gel (1 mL) was added to the lysate and incubated for 2 h, 4 °C, rotating. Resin was collected in a 20 mL column and washed with 15 CV of buffer A (50 mM HEPES-KOH, 300 mM NaCl, 10% glycerol, 1 mM DTT, 0.02% NP-40). CST was eluted with 1 CV buffer A + 0.5 mg/ml 3× FLAG peptide after incubating for 10 min, 2 CV buffer A + 0.25 mg/ml 3× FLAG peptide after incubating for 5 min, and 3 CV of buffer A. Eluates were pooled and concentrated with an Amicon Ultra-15 30 kDa MWCO concentrator and applied onto a Superdex 200 Increase 10/300 GL column (GE Healthcare) equilibrated with Buffer A. Peak fractions were pooled, concentrated in an Amicon Ultra-4 30 kDa MWCO concentrator, aliquoted, frozen in liquid nitrogen, and stored at −80 °C.

#### CST- Pol α/prim complex

CST-Pol α/prim complex was purified as described (He et al, 2022).

### In vitro replication reactions

Replication assays were carried out at 37 °C in three steps based on previously published work (Baris et al, 2022; Georgescu et al, 2014). The final replication buffer contained 25 mM HEPES-KOH pH 7.6, 80 mM potassium L-glutamate, 10 mM Mg(OAc)_2_, 5% glycerol, 40 μg/ml BSA, 1 mM TCEP, and 0.1 mM EDTA. Unless stated in the figure legend, the final concentrations of DNA, protein and nucleotides were: 1.5 nM DNA template, 1.2 or 0.45 nM initiating oligonucleotide (in the absence or presence of Pol δ, Pol α/prim and RFC1-5 respectively), 40 nM CMG, 20 nM Pol ε, 50 nM PCNA, 20 nM CTF18-RFC, 20 nM AND-1, 20 nM Claspin, 20 nM Tipin-Timeless, 100 nM RPA, 1.25 nM RFC, 1.25 nM Pol δ, 20 nM of Pol α/prim, 20 nM FEN1, 40 nM LIG1, 5 mM ATP, 60 μM dA/C/T/GTP, 200 μM CTP/UTP/GTP, and 66 nM α-^32^P-dCTP.

Reactions were set up as follows: the initiating oligonucleotide (see Reagents and Tools Table for sequence) was incubated with 3 nM template for 15 min in reaction buffer supplemented with 0.1 mM AMP-PNP. Subsequently, CMG was loaded onto the primed DNA substrate at a final concentration of 80 nM in a 5 μl reaction and incubated for 10 min. The reaction was subsequently diluted to 7.5 μl by adding reaction buffer supplemented with 240 μM dA/dCTP and 0.1 mm AMP-PNP, and a premixed solution of Pol ε, PCNA, CTF18-RFC, AND-1, Claspin, Tipin-Timeless, and Pol α/prim (in reactions competent for lagging strand synthesis) and the reaction was incubated for 2 min.

For leading strand only reactions, replication was initiated by staggering the addition of 2.5 μl of reaction buffer supplemented with 20 mM ATP, 240 μM dT/GTP, 62 nM α-^32^P-dCTP, and RPA. For reaction competent for lagging strand synthesis, replication was initiated by adding 2.5 μl of reaction buffer supplemented with 20 mM ATP, 240 μM dT/GTP, 800 μM CTP/UTP/GTP, 62 nM α-^32^P-dCTP, RPA, RFC, Pol δ. For lagging strand maturation, FEN1 and LIG1 were added during the initiation step.

Where indicated BLM, WRN, FANCJ, shelterin, shelterin variants, LacI, CST- Pol α/prim, or phenDC3 were added in the last initiation mix or spiked in after initiation. For Figs. 5B and EV7A, shelterin lacking TPP1/POT1 was preincubated with wt TPP1/POT1 or TPP1/POT1 variants for 10 min. For P1 sensitivity assays, the amount of P1 nuclease (NEB, M0660) indicated in the figure legend was added 5 min before the reaction was quenched. Pulse-chase experiments were performed using the conditions for standard leading strand replication reactions, except that the concentration of dCTP was reduced to 20 μM during the pulse. In all, 600 µM unlabelled dA/G/C/TTP was added after 50 s.

### Molecular weight markers

For molecular weight marker, 12.5 μg HindIII-digested phage lambda DNA (NEB, N3012) was dephosphorylated in 1× rCutSmart buffer (NEB, B6004) with 10 U of Quick-CIP (NEB, M0525) in a final volume of 30 μl for 1 h at 37 °C. The phosphatase was inactivated at 80 °C for 2 min and the DNA was purified through a Microspin G-50 column (Cytiva, 27533002) and stored at −20 °C. For analysis on a 6% Urea-PAGE sequencing gel a 50 bp DNA ladder was used (NEB, N3236). The marker was end labelled in a 10 μl reaction with 2 μl of dephosphorylated marker, γ-^32^P-ATP (0.01 mCi), 1× T4 T4 Polynucleotide Kinase (PNK) buffer and 6 U of T4 PNK enzyme (NEB, M0201). The reaction was incubated for 30 min at 37 °C, followed by heat inactivation of PNK at 65 °C for 20 min. Unincorporated radiolabelled nucleotides were removed by passing the sample through a Microspin G-50 column and stored at −20 °C.

### Processing and analysis of replication products

Reactions were quenched at the indicated time by addition of an equal volume of stop buffer containing 50 mM EDTA, 0.4% SDS, and 0.4 mg/ml Proteinase K. After 20 min at 37 °C, an equal volume of phenol:chloroform:isoamyl alcohol (25:24:1) was added and the aqueous phase collected after centrifugation (14,000× *g*, 5 min). Unincorporated radiolabelled nucleotides were removed by passing the sample through a Microspin G-50 column.

For nicking assays, reaction products were digested with 10 U of Nb.BsrDI (NEB) for 2 h at 37 °C. For denaturing alkaline agarose electrophoresis, the samples and a molecular weight marker were supplemented with 20 mM EDTA, 50 mM NaOH, and 0.5% sucrose with xylene cyanol. Reactions were analysed by 0.7% denaturing alkaline agarose electrophoresis in a 20 × 17 cm cassette supplemented with 2 mM EDTA and 30 mM NaOH and run for 16 h at 24 V in 2 mM EDTA and 30 mM NaOH. Gels were fixed in 5% trichloroacetic acid for 30 min. For native agarose electrophoresis, the samples and a molecular weight marker were supplemented with 6X NEB Purple Loading Dye (NEB, B7024). Reactions were analysed by 0.7% agarose/TAE electrophoresis for 20 h at 30 V at 4 °C in a 20 × 17 cm cassette. For 2D native-denaturing electrophoresis analysis, replication products were analysed by 0.7% native agarose electrophoresis as above, the lane excised and separated by 0.7% denaturing alkaline agarose electrophoresis.

Gels were dried on filter paper before being exposed to a Storage Phosphor Screen (GE Healthcare, BAS-IP MS 2025, GE28-9564-75) and imaged on a Typhoon scanner (Cytiva). Images were analysed and quantified in ImageJ and graphs were made using Prism.

### Quantification of shelterin stall and P1 cleavage

Images were analysed and quantified in ImageJ. For the stall: both full length and stall signals were measured after denaturing alkaline agarose. Signal intensity was normalised to product length to account for differences in the incorporation of radiolabel and the background removed. The percentage stall signal was then calculated from the total signal of the FL and stall. For P1 cleavage product: both full length and P1 cleavage signals after native agarose electrophoresis were measured. Signal intensity was normalised to product length to account for differences in the incorporation of radiolabel and the background removed. The % P1 cleavage signal was then calculated from the total signal of the FL and cleaved product. Whether samples were compared relative to a control is indicated in the figure legends.

### Analysis of shelterin stall

For analysis of the shelterin stall position, 60 μl replication reactions were processed and cleaned-up by phenol-chloroform extraction, a Microspin G-50 column and ethanol precipitation as above. To immobilise replication products via the biotinylated initiating oligonucleotide, 12 μl of streptavidin beads (Invitrogen, 11205D) were washed twice with 10 mM Tris-HCl pH 7.5, 1 mM EDTA, 2 M NaCl and incubated with quenched replication reactions overnight at 4 °C with shaking. Pelleted beads were washed twice with 10 mM HEPES-KOH pH 7.6, 1 M KOAc, 1 mM EDTA, twice with 10 mM HEPES-KOH pH 7.6, 1 mM EDTA and resuspended in 20 μl of the same buffer.

Bead-bound DNA was digested with 10 U of NdeI (NEB) and 10 U PstI-HF (NEB) in a final reaction volume of 30 μl for 1 h at 37 °C with shaking, releasing DNA downstream of the NdeI site into the supernatant, which was added to an equal volume of stop mix containing 50 mM EDTA, 0.4% SDS, and 0.4 mg/ml Proteinase K. Following 20 min at 37 °C, the DNA was purified by phenol-chloroform extraction and ethanol precipitation, resuspended in 1 μl in TE pH 8.0 and 2 μl 99% formamide, 5 mM EDTA, bromophenol blue, xylene cyanol, boiled and separated by 6% Urea-PAGE for 4 h at 55 W. Dried gels were exposed to a Storage Phosphor Screen (GE Healthcare, BAS-IP MS 2025, GE28-9564-75) and imaged on a Typhoon scanner (Cytiva). Images were analysed and quantified in ImageJ as described in the figure legends.

### Helicase assay

CLB353 (see Reagents and Tools Table) was end labelled at a final concentration of 2 μM in a 10 μl reaction with γ-^32^P-ATP (0.02 mCi), 1× PNK buffer and 6 U of T4 PNK enzyme (NEB, M0201). The reaction was incubated for 30 min at 37 °C, followed by heat inactivation of PNK for 20 min at 65 °C for 20 min. Unincorporated radiolabelled nucleotides were removed by passing the sample through a G50 column. To make the forked DNA duplex, radiolabelled CLB353 and non-radiolabelled CLB354 (see Reagents and Tools Table) were heated to 95 °C for 5 min in TE buffer supplemented with 100 mM NaCl and cooled to room temperature at a rate of −1 °C/min to facilitate annealing. The non-radiolabelled oligonucleotide was at a twofold molar excess to the radiolabelled oligo.

For Fig. EV2G, BLM at the indicated concentrations was incubated with 1.5 nM of labelled forked DNA duplex for 5 min at 37 °C in a 10 μl reaction containing 20 mM Tris-HCl pH 7.5, 2 mM MgCl_2_, 1 mM TCEP, 0.1 mg/ml BSA. To stimulate unwinding, ATP was added to a final concentration of 5 mM along with 50 nM of unlabelled CLB353 to trap any unwound oligonucleotides and prevent substrate reannealing. Reactions were incubated at 37 °C for 10 min. For Fig. EV7F, BLM/WRN/FANCJ at the indicated concentrations were incubated with 1.5 nM of labelled forked DNA duplex and 10 nM RPA for 5 min at 37 °C in a 10 μl reaction containing 40 mM Tris-HCl pH 7.5, 40 mM KCl, 2 mM MgCl_2_, 1 mM TCEP, 0.1 mg/ml BSA. Unwinding reactions were stimulated as above and reactions were incubated at 37 °C for 15 min.

Reactions were quenched by addition of EDTA to 40 mM and SDS to 0.1% as well as xylene cyanol and bromophenol blue. Samples were analysed on 10% Novex TBE gels (ThermoFisher Scientific, LC6678) in 1× TBE (90 mM Tris-base, 90 mM Boric acid, 2.5 mM EDTA) at 90 V for 90 min. Gels were dried before being exposed to a Storage Phosphor Screen and imaged on a Typhoon scanner. Images were analysed and quantified in ImageJ.

### Immunoblotting

Recombinant shelterin and shelterin variants were resolved using 4–12% or 7.5% Tris-Glycine SDS-PAGE, as indicated, and transferred to a nitrocellulose membrane. Western blot was performed with 5% milk in TBS containing 0.1% Tween-20. TRF1 was detected with a custom-made rabbit antibody (a kind gift from Prof. Sebastian Guettler) at 1:5000 dilution. TRF2 was detected with antibody D1Y5D (Cell Signalling, 13136) at 1:2000 dilution. RAP1 was detected with anti-RAP1 (Bethyl Laboratories, A300-306A) at 1:4000 dilution. POT1 was detected with antibody EPR6319 (Abcam, ab124784) at 1:2000 dilution. TPP1 was detected with anti-ACD (Sigma, WH0065057M2) at 1:5000 dilution. Strep-TIN2 was detected with antibody StrepMAB-Classic (IBA Lifesciences, 21507001) at 1:2000 dilution. Primary antibodies were followed by secondary antibody goat anti-rabbit IgG (LI-COR, 926-68071), goat anti-mouse IgG (LI-COR, 926-68070), goat anti-rabbit IgG (LI-COR, 926-32211), or goat anti-mouse IgG (LI-COR, 926-32210) at 1:5000 dilution. Immunoblots were visualised with the LI-COR Odyssey imaging system according to the manufacturer’s instructions. Images were analysed in Image Studio.

### Mass photometry

Shelterin was diluted to 100 nM immediately prior to measurement in buffer A (50 mM HEPES-KOH pH 7.6, 300 mM NaCl, 1 mM DTT) and 2 μl was added to 12  μl of buffer A in a sealed well on a glass slide. Data collection was carried out on a Refeyn One^MP^ photometer as described previously (Koliopoulos et al, 2022). Mass photometry movies were acquired for 60 s in movies of 6000 frames and processed using the Refeyn Discover^MP^ 2.3.0 software. Data were plotted as normalised histograms with a bin width of 13.6. For the calibration, 2 μl of 1:100 diluted NativeMark unstained protein standard (ThermoFisher, LC0725) was used to generate a standard calibration curve from masses 66, 146, 480, 1048 kDa in the Discover software.

### Electrophoretic mobility shift assay

TBL297 (see Reagents and Tools Table) was end labelled at a final concentration of 2 μM in a 10 μl reaction with γ-^32^P-ATP (0.02 mCi), 1X T4 PNK buffer and 6 U of T4 PNK enzyme (NEB, M0201). The reaction was incubated for 30 min at 37 °C, followed by heat inactivation of PNK for 20 min at 65 °C for 20 min. Unincorporated radiolabelled nucleotides were removed by passing the sample through a G50 column. To make the forked DNA duplex, radiolabelled TBL297 and non-radiolabelled TBL296 (see Reagents and Tools Table) were heated to 95 °C for 5 min in TE buffer supplemented with 100 mM NaCl and cooled to room temperature at a rate of −1 °C/min to facilitate annealing. The non-radiolabelled oligonucleotide was at a 1.2-fold molar excess to the radiolabelled oligo.

For the EMSA, labelled DNA duplex was incubated for 30 min on ice at a final concentration of 2 nM with the shelterin concentrations indicated in a buffer containing 20 mM HEPES-KOH pH 8 and 100 mM NaCl. Where indicated non-radiolabelled competing forked DNA duplex (TBL296/TBL297 for telomeric and TBL337/TBL338) was included with the radiolablled duplex. Samples were supplemented with 6X NEB Purple Loading Dye, no SDS (NEB, B7025) and analysed by 1% agarose electrophoresis in 0.5× TBE at 100 V for 1.5 h in 0.5× TBE. Gels were dried on filter paper before being exposed to a Storage Phosphor Screen and imaged on a Typhoon scanner (Cytiva). Images were analysed and quantified in ImageJ.

### Electron microscopy analysis

For EM analysis, replication assays were performed using the conditions for standard leading and lagging strand replication reactions, except radiolabelled nucleotides were omitted and each sample condition was 50 μl total. Reactions were quenched by addition of EDTA to 5 mM at the times indicated in the figure legend. For crosslinking, samples were pipetted onto parafilm, and trioxsalen (Sigma, T6137) added to a final concentration of 2 μg/ml. Samples were exposed to 365 nm UV light for 5 min using a UVP Blak Ray Lamp Model UVL-21, with 365 nm UV bulbs at 2–3 cm from the light source. Samples were returned to a 1.5 ml Eppendorf. The reaction was quenched by addition of stop buffer as described above, and the DNA obtained by phenol-chloroform extraction and ethanol precipitation. The resulting pellet was resuspended in 8 μl of TE buffer and the concentration measured by a Qubit 4 Fluorometer (ThermoFisher).

For EM analysis, 5 µl of DNA in TE was mixed with 5 μl of formamide (Thermo Scientific, 17899) and 0.4 μl of 0.02% benzalkonium chloride (BAC, Sigma B6285) in TE. Immediately after mixing, the drop was allowed to slide through a freshly cleaved mica sheet (Ted Pella Inc, product no: 52-6) used as a ramp inside a 15 cm dish containing 50 ml of distilled water. The monomolecular layer above the water surface was gently touched with a carbon-coated copper grid, previously placed in contact with an ethidium bromide droplet (33.3 μg/ml in H_2_O) for 30–45 min. Carbon grids with absorbed DNA molecules were immediately stained with a solution of uranyl acetate 0.2 μg/μl in ethanol and subjected to low-angle rotary shadowing with 8 nm of platinum using a MED020 evaporator, modified with the low-angle grid shadowing kit (Leica 16770525).

TEM pictures were taken using a FEI Tecnai12 Bio twin microscope operated at 120 KV and equipped with a side-mounted GATAN Orius SC-1000 camera controlled by the Digital Micrograph software. For acquisition of large areas, overlapping fields were acquired and stitched using the Digital Micrograph software. Micrographs in DM3 format were analysed in FIJI/ImageJ. In these conditions, in BAC spreads, a length of 0.36 μm corresponds to 1 kb of double-stranded DNA. Images were randomised before scoring. Data annotation and storage during the analysis was performed with an ImageJ macro (Mazzucco et al, 2022)

### TPP1/POT1 and CST Strep pulldown

CST (300 nM) and Strep-TPP1/POT1 (100 nM) were incubated together for 30 min, at 4 °C in a final volume of 10 μl. The final buffer A contained 25 mM HEPES-KOH pH 7.6, 80 mM potassium L-glutamate, 10 mM Mg(OAc)_2_, 5% glycerol, 0.02% Tween-20, 1 mM TCEP, and 0.1 mM EDTA. MagStrep Streptactin XT beads (IBA Lifesciences, 2-5090-002) were washed with buffer A + /− 100 mM NaCl, resuspended to a 1:10 dilution and 10 μl added to each reaction for 30 min at 4 °C, shaking. Beads were pelleted on a magnetic rack and washed three times with buffer A + /− 100 mM NaCl, resuspended in 10 μl of buffer A and supplemented with SDS loading buffer before boiling and resolving by 4–12% Tris-Glycine SDS-PAGE. Products were analysed by a SilverQuest silver staining kit (Invitrogen LC6070).

### TPP1/POT1 and CST binding to DNA

In all, 30 µl M280 Dynabeads (ThermoFisher, 11205D) washed twice with TE pH 8.0 + 2 M NaCl and twice with TE pH 8.0 + 100 mM NaCl were incubated with 4 μl of oligonucleotide CLB401 (100 μM stock, (see Reagents and Tools Table) at 23 °C for 4 h with shaking, washed as above and resuspended in 30 μl of TE pH 8.0 + 100 mM NaCl.

The final reaction volume of 60 µl contained buffer A: 25 mM HEPES-KOH pH 7.6, 80 mM potassium L-glutamate, 10 mM Mg(OAc)_2_, 5% glycerol, 0.02% Tween-20, 1 mM TCEP, and 0.1 mM EDTA and 1 μl of beads. CST at the indicated concentrations was diluted in buffer and mQ before the addition of TPP1/POT1 immediately prior to the 1 μl of beads. Reactions were incubated for 15 min at 37 °C with shaking, beads washed three times with buffer A, resuspended in 10 μl of buffer A and supplemented with SDS loading buffer before boiling and resolving by 4–12% Tris-Glycine SDS-PAGE. Products were analysed by a SilverQuest silver staining kit (Invitrogen LC6070).

## Supplementary information

Appendix

Peer Review File

Source data Fig. 1

Source data Fig. 2

Source data Fig. 3

Source data Fig. 4

Source data Fig. 5

Expanded View Figures

## Data Availability

This study includes no data deposited in external repositories. The source data of this paper are collected in the following database record: biostudies:S-SCDT-10_1038-S44318-026-00844-7.
