# Supplementary material for: Inhibition of lagging strand replication by G-rich telomeric DNA and the shelterin subunit POT1
Source: EMBO J. 2026 Jul 9;45(16):5793–826. doi: 10.1038/s44318-026-00844-7 (PMC13483900; doi:10.1038/s44318-026-00844-7)

Appendix for:

Inhibition of lagging strand replication by G-rich telomeric DNA and the shelterin subunit POT1

Ciara Leonard-Booker[1], Giulia Mazzucco[2,4], Charlotte E.L. Fisher[1], Karim Hussain[1], Tom D. Deegan[3], Ylli Doksani[2] and Max E. Douglas[1*]

1. Telomere Biology Laboratory, The Institute of Cancer Research, 237 Fulham Road, London SW3 6JB, UK
2. IFOM ETS-The AIRC Institute of Molecular Oncology, Milan, Italy.
3. MRC Human Genetics Unit, University of Edinburgh, EH4 2XU, UK
4. Current affiliation, 4bases Italia S.r.l.. Viale A. Brambilla 60 27100 Pavia (PV) Italy

*****Correspondence: max.douglas@icr.ac.uk

Table of Contents

Appendix Text S1 - sequences of DNA constructs for in vitro replication page 3

Appendix Figure S1 - lane scans supporting figure 2 page 18

Appendix Text S1:

Control (for 240 TTAGGG)

CCTCTGACACATGCAGCTCCCGGACCTCTAGCCCTGCCCAGCGGAGAAGAGGCAACGACCCTCTGACAAGCAGCCCCGG
CAGAAGCAGCAGAAGAACCGACGCCCTGACCAGCTCCCCTGGCAGAGATCTGCCCCCATTCGAGGATGAGAGCGAGGGC
CTGCTGGGCACAGAGGGACCTCTGGAAGAAGAAGAGGACGGCGAGGAACTGATCGGCGACGGCATGGAACGGGACTAC
AGAGCCATCCCCGAGCTGGATGCCTATGAGGCCGAAGGCCTGGCCCTGGACGACGAGGATGTGGAAGAACTGACCGCC
AGCCAGAGAGAGGCCGCCGAGAGAGCTATGCGGCAGAGAGACAGAGAGGCTGGCAGAGGCCTGGGCAGAATGCGGAG
AGGCCTGCTGTACGACAGCGACGAGGAAGATGAGGAAAGACCCGCCAGAAAGCGGAGACAGGTGGAAAGAGCCACCGA
GGACGGGGAAGAGGATGAAGAGATGATCGAGAGCATCGAGAACCTGGAAGATCTGAAGGGCCACAGCGTGCGCGAGTG
GGTGTCAATGGCTGGCCCCAGACTGGAAATCCACCACCGGTTCAAGAACTTTCTGCGGACCCACGTGGACAGCCACGGC
CACAACGTGTTCAAAGAACGGATCAGCGACATGTGCAAAGAGAACCGCGAGAGCCTGGTCGTGAACTACGAGGATCTGG
CCGCCAGAGAACACGTGCTGGCCTACTTTCTGCCTGAGGCCCCTGCCGAGCTGCTCCAGATCTTTGATGAGGCCGCCCT
GGAAGTGGTGCTGGCCATGTACCCTAAGTACGACCGGATCACCAACCACATCCACGTGCGGATCAGCCATCTGCCCCTG
GTGGAAGAACTGCGGAGCCTGAGACAGCTGCACCTGAACCAGCTGATCCGGACCTCCGGCGTCGTGACCTCTTGTACAG
GCGTGCTGCCCCAGCTGTCTATGGTCAAGTACAACTGCAACAAGTGCAACTTCGTGCTGGGACCCTTCTGCCAGAGCCAG
AACCAGGAAGTGAAGCCCGGCAGCTGTCCCGAGTGTCAGAGCGCCGGACCCTTCGAAGTGAACATGGAAGAGACAATCT
ACCAGAACTACCAGCGCATCCGCATAATACGACTCACTATAGGCATATGCGGTGTGAAATACCGCACAGATGCGTAAGGA
GAAAATACCGCATCAGGCGCCATTCGCCATTCAGGCTGCGCAACTGTTGGGAAGGGCGATCGGTGCGGGCCTCTTCGCT
ATTACGCCAGCTGGCGAAAGGGGGATGTGCTGCAAGGCGATTAAGTTGGGTAACGCCAGGGTTTTCCCAGTCACGACGTT
GTAAAACGACGGCCAGTGAATTCGTCCTGAAGGCTAAGCTGCAAGAGGCCATGAAGCTGCGCCGCTTCGAAGAAAGGCA
GAAGCGTCAGGCTCTGTTCAAGCTGGACAACGAGGACGGTTTCGAAGAAGAAGAAGAGGAAGAGGAAGAAATGACCGAC
GAGTCCGAAGAGGACGGCGAGGAAAAGGTCGAAAAAGAGGAAAAAGAAGAAGAACTCGAGGAAGAAGAGGAAAAAGAAG
AGGAAGAAGAAGAAGAGGGCAATCAAGAGACTGCCGAGTTCCTGCTGTCCTCCGAAGAAATCGAGACAAAGGACGAGAAA
GAAATGGACAAAGAAAACAACGACGGCTCCTCCGAGATCGGCAAGGCTGTGGGTTTCCTGTCCGTGCCTAAGTCTCTGTC
CTCTGACTCTACCCTGCTGCTGTTCAAGGACTCCTCCAGCAAGATGGGCTACTTCCCCACCGAAGAGAAGTCCGAAACCG
ACGAGAACTCCGGCAAGCAGCCCTCTAAGTTGGACGAGGACGACTCCTGCTCTCTGCTGACCAAAGAGTCCAGCCACAAC
TCCAGCTTCGAGCTGATCGGTTCTACTATCCCATCCTACCAGCCTTGCAACCGTCAGACCGGTCGTGGTACTTCTTTCTTC
CCTACCGCTGGTGGTTTCAGGTCCCCTTCTCCTGGACTGTTCCGTGCTTCCCTGGTTTCCTCCGCTTCCAAGTCCTCCGGA
AAGCTGTCCGAGCCTTCTCTGCCTATCGAGGACTCCCAGGACCTGTACAACGCTTCCCCTGAGCCTAAGACACTGTTCCT
CGGTGCTGGCGACTTCCAGTTCTGCCTTGAGGACGACACTCAGTCCCAGCTGTTGGACGCTGACGGTTTCCTGAACGTGC
GTAACCACCGTAACCAGTACCAGGCTTTGAAGCCTCGTCTGCCCCTGGCTTCCATGGACGAGAATGCTATGGACGCTAAC
ATGGACGAACTGCTGGACCTGTGCACCGGCAAGTTCACTAGCCAGGCTGAGAAGCACTTGCCTCGCAAGTCCGACAAGAA
AGAAAACATGGAAGAGTTGCTGAACCTCTGCTCGGGAAAGTTCACCAGTCAGGACGCTTCTACCCCTGCTTCCTCTGAGCT
GAACAAGCAAGAGAAAGAATCCTCCATGGGCGACCCTATGGAAGAGGCTTTGGCTCTGTGCTCCGGTAGCTTCCCAACTG
ACAAAGAGGAAGAGGACGAAGAGGAAGAGTTCGGCGACTTCCGTCTGGTGTCCAACGACAACGAGTTCGACTCTGATGA
GGACGAGCACAGCGACTCTGGCAACGACTTGGCTCTGGAAGATCACGAGGATGACGACGAAGAAGAATTGCTGAAGCGT
TCCGAGAAGCTCAAGCGTCAGATGCGTCTGCGCAAGTACCTCGAGGATGAGGCTGAGGTGTCCGGTTCTGACGTCGGTT
CAGAGGACGAGTACGATGGCGAAGAAATTGACGAATACGAGGAAGATGTGATCGACGAGGTGCTGCCCAATTGGAGGAA
CTGCAGAGTCATTCTGAGAATAGTGTATGCGGCGACCGAGTTGCTCTTGCCCGGCGTCAATACGGGATAGTACCGCGCCA
CATAGCAGCACTTTGAAAGTGCTCATCATTGGAAAACGTTCTTCGGGGCGAAAACTCTCAAGGATCTTACCGCTGTTGAGA
TCCAGTTCGATGTAACCCACTCGTGCACCCAACTGATCTTCAGCATCTTTTACTTTCACCAGCGTTTCTGGGTGAGCAAGAA
CAGGAAGGCACAATGCCGCAGACAAGGGAATAAGGGCGACACGGAAATGTTGAATACTCATACTCTTCCTGCTTCAATAGT
ATTGAAGCATCTATCAGGGTTAGTGTCTCATGAGCGGATACATATCTGAATGTATGTAGAACACTAGACACATAGGGGTTC
CGCGCACATTTCCCCGAGAAGTGCCACCTGACGTCTAAGACACCAGTATCATCATGACATTGACCTCTAGACgGATCCGGG
TCGACCGCCACCATGAGCGGCTTTGACGACCCCGGCATCTTCTACAGCGACAGCTTCGGCGGAGATGCCCAGGCCGATG
AAGGCCAGGCCAGAAAGTCTCAGCTCCAGCGGCGGTTCAAAGAGTTCCTGCGGCAGTACAGAGTGGGCACCGACAGAAC
CGGCTTCACCTTCAAGTACCGGGACGAGCTGAAGCGGCACTACAACCTGGGCGAGTACTGGATCGAGGTGGAAATGGAA
GATCTGGCCAGCTTCGACGAGGACCTGGCCGACTACCTGTACAAGCAGCCCGCCGAGCATCTCCAGCTGCTGGAAGAGG
CCGCTAAAGAGGTGGCCGACGAAGTGACCAGACCCAGGCCTTCTGGCGAGGAAGTGCTCCAGGACATCCAAGTGATGCT

GAAGTCCGACGCCAGCCCCAGCAGCATCAGATCCCTGAAGTCTGACATGATGAGCCACCTCGTGAAGATCCCCGGAATCA
TCATTGCCGCCTCTGCCGTGCGGGCCAAGGCCACCAGAATCAGCATCCAGTGCAGAAGCTGCCGGAACACCCTGACCAA
TATCGCCATGCGCCCTGGCCTGGAAGGCTACGCCCTGCCCAGAAAGTGCAACACCGACCAGGCTGGCAGACCCAAGTGC
CCCCTGGACCCCTACTTCATCATGCCCGACAAGTGCAAATGCGTGGACTTCCAGACCCTGAAACTCCAGGAACTGCCCGA
CGCCGTGCCTCACGGCGAAATGCCTAGACACATGCAGCTGTACTGCGACAGATACCTGTGCGACAAGGTGGTGCCCGGC
AACAGAGTGACCATCATGGGCATCTACAGCATCAAGAAGTTCGGCCTGACCACCAGCCGGGGCAGAGATAGAGTGGGCG
TGGGCATCCGGTCCAGCTACATCAGAGTGCTGGGCATCCAGGTGGACACCGATGGCAGCGGCAGATCCTTTGCTGGGGC
CGTGAGCCCCCAGGAAGAGGAAGAGTTTAGACGGCTGGCCGCCCTGCCTAACGTGTACGAAGTGATCAGCAAGTCTATC
GCCCCCAGCATCTTCGGCGGCACCGATATGAAGAAGGCCATTGCCTGCCTGCTGTTCGGCGGCTCCAGAAAGAGACTGC
CTGACGGCCTGACAAGACGGGGCGACATCAACCTGCTGATGCTGGGCGATCCTGGCACCGCCAAATCACAGCTGCTGAA
GTTCGTGGAAAAGTGCAGCCCCATCGGCGTGTACACCAGCGGCAAGGGATCTTCTGCCGCCGGACTGACAGCCAGCGTG
ATGAGAGATCCCAGCAGCCGGAATTTCATCATGGAAGGCGGCGCTATGGTGCTGGCCGATGGCGGAGTCGTGTGCATCG
ACGAGTTCGACAAGATGCGCGAGGACGACCGGGTGGCCATTCACGAGGCTATGGAACAGCAGACCATCTCTATCGCCAA
GGCCGGAATCACCACCACCCTGAACAGCCGGTGTAGCGTGCTGGCTGCCGCCAATAGCGTGTTCGGCAGATGGGACGAG
ACAAAGGGCGAGGACAACATCGACTTCATGCCCACCATCCTGAGCCGCTTCGACATGATCTTCATCGTGAAGGACGAGCA
CAACGAGGAACGGGACGTGATGCTGGCCAAGCACGTGATCACCCTGCACGTGTCCGCCCTGACACAGACACAGGCCGTG
GAAGGCGAGATCGACCTGGCCAAACTGAAGAAGTTTATCGCCTACTGTCGCGTGAAGTGCGGCCCCAGACTGTCTGCCG
AGGCCGCCGAGAAGCTGAAGAACCGGTACATCATCATGCGGAGCGGAGCCCGGCAGCACGAGAGAGACAGCGATCGGA
GAAGCAGCATCCCCATCACCGTGCGGCAGCTGGAAGCCATCGTGCGGATTGCCGAAGCCCTGAGCAAGATGAAGCTCCA
GCCCTTCGCCACCGAGGCCGACGTGGAAGAAGCTCTGAGACTGTTCCAGGTGTCAACCCTGGACGCCGCCCTGAGCGGA
ACACTGTCTGGCGTGGAAGGATTCACCAGCCAGGAAGATCAGGAAATGCTGAGCAGAATCGAGAAACAGCTGAAGAGAAG
ATTCGCCATCGGAAGCCAGGTGTCCGAGCACAGCATCATCAAGGACTTCACCAAGCAGAAGTACCCCGAGCACGCCATCC
ACAAGGTGCTCCAGCTGATGCTGAGAAGAGGCGAGATCCAGCACCGGATGCAGCGGAAGGTGCTGTACCGGCTGAAATG
AGGCGCCGGAATCTTTGCAGGTGCTTACGCTTACTACCTAAACTACAATGGTGTTGTCGCTACTAGTGCCGCTTCTTCATC
CACTGCATCTGGTGCTTCCGCTTCCGTTACCGGTTCTAAGAAACTTCAGAAACTTCAGAAACCAGTGCTGCCCCTAAGACA
TACACTACTGCCACTGTTACTCAATGTGATGACAATGGTTGTAACGTCAAGATAATCACCTCTCAAATACCTGAAGCTACTT
CAACCGTCACCGCAACTAGTGCTTCTCCAAAGTCATACACTACTGTCACTTCTGAGGGTTCTAAAGCAACCTCATTGAGTTC
TCACTGCCCGCTTTCCAGTCGGGAAACCTGTCGTGCCAGCTGCATTAATGAATCGGCCAACGCGCGGGGAGAGGCGGTT
TGCGTATTGGGCGCTCTTCCGCTTCCTCGCTCACTGACTCGCTGCGCTCGGTCGTTCGGCTGCGGCGAGCGGTATCAGC
TCACTCAAAGGCGGTAATACGGTTATCCACAGAATCAGGGGATAACGCAGGACAGAACATGTGAGCAGAAGGCCAGCACG
AGGCCAGGAGCCGTAAGAAGGCCGCGTTGCTGGCGTTTTTCCATAGGCTCCGCCCCCTCGCGAGCTGAGGACTTAAGTG
CTGAGGTCGCGAGCATTCAGAAGAGCCATTCAAGGGATCGATGTGGTCGCACGACTACGCATCCCTCTGGGCCTTACATA
GCCGGATACAGTGACTTATCGATACGCGTGTTAACCTCGAGGCGGCCGCCATGGTGACAGGACTTGCATAGCTGCGTGC
GGGGGAAGGAACTCTTGCGTGTGAGTATGTAGACCCCTGTACTACGGATGCGGGCAGAAGATGTGGGCAGAGACACCCG
CGTCAAGTTCTCGACCTTCCCGTGGGAGGTGTTCCAGTCCGCCATACGACCATACCGTTCGGGCATGGCACTATGTACGC
GATATCGCTAGCGTTTGCGGGGCACAGCAATATGCAGGCATGCAAGCTTGGCGTAATCATGGTCATAGCTGTTTCCTGTGT
GAAATTGTTATCCGCTCACAATTCCACACAACATACGAGCCGGAAGCATAAAGTGTAAAGCCTGGGGTGCCTAATGAGTGA
GCTAACTCACATTAATTGCGTTGCGCTCACTGCCCGCTTTCCAGTCGGGAAACCTGTCGTGCCAGCTGCATTAATGAATCG
GCCAACGCGCGGGGAGAGGCGGTTTGCGTATTGGGCGCTCTTCCGCTTCCTCGCTCACTGACTCGCTGCGCTCGGTCGT
TCGGCTGCGGCGAGCGGTATCAGCTCACTCAAAGGCGGTAATACGGTTATCCACAGAATCAGGGGATAACGCAGGAAAG
AACATGTGAGCAAAAGGCCAGCAAAAGGCCAGGAACCGTAAAAAGGCCGCGTTGCTGGCGTTTTTCCATAGGCTCCGCCC
CCCTGACGAGCATCACAAAAATCGACGCTCAAGTCAGAGGTGGCGAAACCCGACAGGACTATAAAGATACCAGGCGTTTC
CCCCTGGAAGCTCCCTCGTGCGCTCTCCTGTTCCGACCCTGCCGCTTACCGGATACCTGTCCGCCTTTCTCCCTTCGGGA
AGCGTGGCGCTTTCTCATAGCTCACGCTGTAGGTATCTCAGTTCGGTGTAGGTCGTTCGCTCCAAGCTGGGCTGTGTGCA
CGAACCCCCCGTTCAGCCCGACCGCTGCGCCTTATCCGGTAACTATCGTCTTGAGTCCAACCCGGTAAGACACGACTTAT
CGCCACTGGCAGCAGCCACTGGTAACAGGATTAGCAGAGCGAGGTATGTAGGCGGTGCTACAGAGTTCTTGAAGTGGTG
GCCTAACTACGGCTACACTAGAAGAACAGTATTTGGTATCTGCGCTCTGCTGAAGCCAGTTACCTTCGGAAAAAGAGTTGG
TAGCTCTTGATCCGGCAAACAAACCACCGCTGGTAGCGGTGGTTTTTTTGTTTGCAAGCAGCAGATTACGCGCAGAAAAAA
AGGATCTCAAGAAGATCCTTTGATCTTTTCTACGGGGTCTGACGCTCAGTGGAACGAAAACTCACGTTAAGGGATTTTGGT
CATGAGATTATCAAAAAGGATCTTCACCTAGATCCTTTTAAATTAAAAATGAAGTTTTAAATCAATCTAAAGTATATATGAGTA

AACTTGGTCTGACAGTTACCAATGCTTAATCAGTGAGGCACCTATCTCAGCGATCTGTCTATTTCGTTCATCCATAGTTGCC
TGACTCCCCGTCGTGTAGATAACTACGATACGGGAGGGCTTACCATCTGGCCCCAGTGCTGCAATGATACCGCGAGACCC
ACGCTCACCGGCTCCAGATTTATCAGCAATAAACCAGCCAGCCGGAAGGGCCGAGCGCAGAAGTGGTCCTGCAACTTTAT
CCGCCTCCATCCAGTCTATTAATTGTTGCCGGGAAGCTAGAGTAAGTAGTTCGCCAGTTAATAGTTTGCGCAACGTTGTTG
CCATTGCTACAGGCATCGTGGTGTCACGCTCGTCGTTTGGTATGGCTTCATTCAGCTCCGGTTCCCAACGATCAAGGCGA
GTTACATGATCCCCCATGTTGTGCAAAAAAGCGGTTAGCTCCTTCGGTCCTCCGATCGTTGTCAGAAGTAAGTTGGCCGCA
GTGTTATCACTCATGGTTATGGCAGCACTGCATAATTCTCTTACTGTCATGCCATCCGTAAGATGCTTTTCTGTGACTGGTG
AGTACTCAACCAAGTCATTCTGAGAATAGTGTATGCGGCGACCGAGTTGCTCTTGCCCGGCGTCAATACGGGATAATACC
GCGCCACATAGCAGAACTTTAAAAGTGCTCATCATTGGAAAACGTTCTTCGGGGCGAAAACTCTCAAGGATCTTACCGCTG
TTGAGATCCAGTTCGATGTAACCCACTCGTGCACCCAACTGATCTTCAGCATCTTTTACTTTCACCAGCGTTTCTGGGTGAG
CAAAAACAGGAAGGCAAAATGCCGCAAAAAAGGGAATAAGGGCGACACGGAAATGTTGAATACTCATACTCTTCCTTTTTC
AATATTATTGAAGCATTTATCAGGGTTATTGTCTCATGAGCGGATACATATTTGAATGTATTTAGAAAAATAAACAAATAGGG
GTTCCGCGCACATTTCCCCGAAAAGTGCCACCTGACGTCTAAGAAACCATTATTATCATGACATTAACCTATAAAAATAGGC
GTATCACGAGGCCCTTTCG

240 TTAGGG telomeric

CCTCTGACACATGCAGCTCCCGGACCTCTAGCCCTGCCCAGCGGAGAAGAGGCAACGACCCTCTGACAAGCAGCCCCGG
CAGAAGCAGCAGAAGAACCGACGCCCTGACCAGCTCCCCTGGCAGAGATCTGCCCCCATTCGAGGATGAGAGCGAGGGC
CTGCTGGGCACAGAGGGACCTCTGGAAGAAGAAGAGGACGGCGAGGAACTGATCGGCGACGGCATGGAACGGGACTAC
AGAGCCATCCCCGAGCTGGATGCCTATGAGGCCGAAGGCCTGGCCCTGGACGACGAGGATGTGGAAGAACTGACCGCC
AGCCAGAGAGAGGCCGCCGAGAGAGCTATGCGGCAGAGAGACAGAGAGGCTGGCAGAGGCCTGGGCAGAATGCGGAG
AGGCCTGCTGTACGACAGCGACGAGGAAGATGAGGAAAGACCCGCCAGAAAGCGGAGACAGGTGGAAAGAGCCACCGA
GGACGGGGAAGAGGATGAAGAGATGATCGAGAGCATCGAGAACCTGGAAGATCTGAAGGGCCACAGCGTGCGCGAGTG
GGTGTCAATGGCTGGCCCCAGACTGGAAATCCACCACCGGTTCAAGAACTTTCTGCGGACCCACGTGGACAGCCACGGC
CACAACGTGTTCAAAGAACGGATCAGCGACATGTGCAAAGAGAACCGCGAGAGCCTGGTCGTGAACTACGAGGATCTGG
CCGCCAGAGAACACGTGCTGGCCTACTTTCTGCCTGAGGCCCCTGCCGAGCTGCTCCAGATCTTTGATGAGGCCGCCCT
GGAAGTGGTGCTGGCCATGTACCCTAAGTACGACCGGATCACCAACCACATCCACGTGCGGATCAGCCATCTGCCCCTG
GTGGAAGAACTGCGGAGCCTGAGACAGCTGCACCTGAACCAGCTGATCCGGACCTCCGGCGTCGTGACCTCTTGTACAG
GCGTGCTGCCCCAGCTGTCTATGGTCAAGTACAACTGCAACAAGTGCAACTTCGTGCTGGGACCCTTCTGCCAGAGCCAG
AACCAGGAAGTGAAGCCCGGCAGCTGTCCCGAGTGTCAGAGCGCCGGACCCTTCGAAGTGAACATGGAAGAGACAATCT
ACCAGAACTACCAGCGCATCCGCATAATACGACTCACTATAGGCATATGCGGTGTGAAATACCGCACAGATGCGTAAGGA
GAAAATACCGCATCAGGCGCCATTCGCCATTCAGGCTGCGCAACTGTTGGGAAGGGCGATCGGTGCGGGCCTCTTCGCT
ATTACGCCAGCTGGCGAAAGGGGGATGTGCTGCAAGGCGATTAAGTTGGGTAACGCCAGGGTTTTCCCAGTCACGACGTT
GTAAAACGACGGCCAGTGAATTCGAGCTCGGTACCCGGGGATCCTCTAGAGTCGACGAAGACTTAGGGTTAGGGTTAGG
GTTAGGGTTAGGGTTAGGGTTAGGGTTAGGGTTAGGGTTAGGGTTAGGGTTAGGGTTAGGGTTAGGGTTAGGGTTAGGGT
TAGGGTTAGGGTTAGGGTTAGGGTTAGGGTTAGGGTTAGGGTTAGGGTTAGGGTTAGGGTTAGGGTTAGGGTTAGGGTTA
GGGTTAGGGTTAGGGTTAGGGTTAGGGTTAGGGTTAGGGTTAGGGTTAGGGTTAGGGTTAGGGTTAGGGTTAGGGTTAG
GGTTAGGGTTAGGGTTAGGGTTAGGGTTAGGGTTAGGGTTAGGGTTAGGGTTAGGGTTAGGGTTAGGGTTAGGGTTAGG
GTTAGGGTTAGGGTTAGGGTTAGGGTTAGGGTTAGGGTTAGGGTTAGGGTTAGGGTTAGGGTTAGGGTTAGGGTTAGGGT
TAGGGTTAGGGTTAGGGTTAGGGTTAGGGTTAGGGTTAGGGTTAGGGTTAGGGTTAGGGTTAGGGTTAGGGTTAGGGTTA
GGGTTAGGGTTAGGGTTAGGGTTAGGGTTAGGGTTAGGGTTAGGGTTAGGGTTAGGGTTAGGGTTAGGGTTAGGGTTAG
GGTTAGGGTTAGGGTTAGGGTTAGGGTTAGGGTTAGGGTTAGGGTTAGGGTTAGGGTTAGGGTTAGGGTTAGGGTTAGG
GTTAGGGTTAGGGTTAGGGTTAGGGTTAGGGTTAGGGTTAGGGTTAGGGTTAGGGTTAGGGTTAGGGTTAGGGTTAGGGT
TAGGGTTAGGGTTAGGGTTAGGGTTAGGGTTAGGGTTAGGGTTAGGGTTAGGGTTAGGGTTAGGGTTAGGGTTAGGGTTA
GGGTTAGGGTTAGGGTTAGGGTTAGGGTTAGGGTTAGGGTTAGGGTTAGGGTTAGGGTTAGGGTTAGGGTTAGGGTTAG
GGTTAGGGTTAGGGTTAGGGTTAGGGTTAGGGTTAGGGTTAGGGTTAGGGTTAGGGTTAGGGTTAGGGTTAGGGTTAGG
GTTAGGGTTAGGGTTAGGGTTAGGGTTAGGGTTAGGGTTAGGGTTAGGGTTAGGGTTAGGGTTAGGGTTAGGGTTAGGGT
TAGGGTTAGGGTTAGGGTTAGGGTTAGGGTTAGGGTTAGGGTTAGGGTTAGGGTTAGGGTTAGGGTTAGGGTTAGGGTTA
GGGTTAGGGTTAGGGTTAGGGTTAGGGTTAGGGTTAGGGTTAGGGTTAGGGTTAGGGTTAGGGTTAGGGTTAGGGTTAG

GGTTAGGGTTAGGGTTAGGGTTAGGGTTAGGGTTAGGGTTAGGGTTAGGGTTAGGGTTAGGGTTAGGGTTAGGGTTAGG
GTTAGGGTTAGGGTTAGGGTTAGGGTTAGGGTTAGGGTTAGGGTTAGGGTTAGGGTTAGGGTTAGGGTTAGGGTTAGGGT
TAGGGTTAGGGTTAGGGTTAGGGTTAGGGTTAGGGTTAGGGTTAGGGTTAGGGTTAGGGTTAGGGTTAGGGCGAGACGT
GTCCCGTCGACCTGCATAGCGCTCAATTGCAACCACTGCAGAGTCATTCTGAGAATAGTGTATGCGGCGACCGAGTTGCT
CTTGCCCGGCGTCAATACGGGATAGTACCGCGCCACATAGCAGCACTTTGAAAGTGCTCATCATTGGAAAACGTTCTTCG
GGGCGAAAACTCTCAAGGATCTTACCGCTGTTGAGATCCAGTTCGATGTAACCCACTCGTGCACCCAACTGATCTTCAGCA
TCTTTTACTTTCACCAGCGTTTCTGGGTGAGCAAGAACAGGAAGGCACAATGCCGCAGACAAGGGAATAAGGGCGACACG
GAAATGTTGAATACTCATACTCTTCCTGCTTCAATAGTATTGAAGCATCTATCAGGGTTAGTGTCTCATGAGCGGATACATA
TCTGAATGTATGTAGAACACTAGACACATAGGGGTTCCGCGCACATTTCCCCGAGAAGTGCCACCTGACGTCTAAGACACC
AGTATCATCATGACATTGACCTCTAGACgGATCCGGGTCGACCGCCACCATGAGCGGCTTTGACGACCCCGGCATCTTCTA
CAGCGACAGCTTCGGCGGAGATGCCCAGGCCGATGAAGGCCAGGCCAGAAAGTCTCAGCTCCAGCGGCGGTTCAAAGA
GTTCCTGCGGCAGTACAGAGTGGGCACCGACAGAACCGGCTTCACCTTCAAGTACCGGGACGAGCTGAAGCGGCACTAC
AACCTGGGCGAGTACTGGATCGAGGTGGAAATGGAAGATCTGGCCAGCTTCGACGAGGACCTGGCCGACTACCTGTACA
AGCAGCCCGCCGAGCATCTCCAGCTGCTGGAAGAGGCCGCTAAAGAGGTGGCCGACGAAGTGACCAGACCCAGGCCTT
CTGGCGAGGAAGTGCTCCAGGACATCCAAGTGATGCTGAAGTCCGACGCCAGCCCCAGCAGCATCAGATCCCTGAAGTC
TGACATGATGAGCCACCTCGTGAAGATCCCCGGAATCATCATTGCCGCCTCTGCCGTGCGGGCCAAGGCCACCAGAATCA
GCATCCAGTGCAGAAGCTGCCGGAACACCCTGACCAATATCGCCATGCGCCCTGGCCTGGAAGGCTACGCCCTGCCCAG
AAAGTGCAACACCGACCAGGCTGGCAGACCCAAGTGCCCCCTGGACCCCTACTTCATCATGCCCGACAAGTGCAAATGCG
TGGACTTCCAGACCCTGAAACTCCAGGAACTGCCCGACGCCGTGCCTCACGGCGAAATGCCTAGACACATGCAGCTGTAC
TGCGACAGATACCTGTGCGACAAGGTGGTGCCCGGCAACAGAGTGACCATCATGGGCATCTACAGCATCAAGAAGTTCGG
CCTGACCACCAGCCGGGGCAGAGATAGAGTGGGCGTGGGCATCCGGTCCAGCTACATCAGAGTGCTGGGCATCCAGGT
GGACACCGATGGCAGCGGCAGATCCTTTGCTGGGGCCGTGAGCCCCCAGGAAGAGGAAGAGTTTAGACGGCTGGCCGC
CCTGCCTAACGTGTACGAAGTGATCAGCAAGTCTATCGCCCCCAGCATCTTCGGCGGCACCGATATGAAGAAGGCCATTG
CCTGCCTGCTGTTCGGCGGCTCCAGAAAGAGACTGCCTGACGGCCTGACAAGACGGGGCGACATCAACCTGCTGATGCT
GGGCGATCCTGGCACCGCCAAATCACAGCTGCTGAAGTTCGTGGAAAAGTGCAGCCCCATCGGCGTGTACACCAGCGGC
AAGGGATCTTCTGCCGCCGGACTGACAGCCAGCGTGATGAGAGATCCCAGCAGCCGGAATTTCATCATGGAAGGCGGCG
CTATGGTGCTGGCCGATGGCGGAGTCGTGTGCATCGACGAGTTCGACAAGATGCGCGAGGACGACCGGGTGGCCATTCA
CGAGGCTATGGAACAGCAGACCATCTCTATCGCCAAGGCCGGAATCACCACCACCCTGAACAGCCGGTGTAGCGTGCTG
GCTGCCGCCAATAGCGTGTTCGGCAGATGGGACGAGACAAAGGGCGAGGACAACATCGACTTCATGCCCACCATCCTGA
GCCGCTTCGACATGATCTTCATCGTGAAGGACGAGCACAACGAGGAACGGGACGTGATGCTGGCCAAGCACGTGATCAC
CCTGCACGTGTCCGCCCTGACACAGACACAGGCCGTGGAAGGCGAGATCGACCTGGCCAAACTGAAGAAGTTTATCGCC
TACTGTCGCGTGAAGTGCGGCCCCAGACTGTCTGCCGAGGCCGCCGAGAAGCTGAAGAACCGGTACATCATCATGCGGA
GCGGAGCCCGGCAGCACGAGAGAGACAGCGATCGGAGAAGCAGCATCCCCATCACCGTGCGGCAGCTGGAAGCCATCG
TGCGGATTGCCGAAGCCCTGAGCAAGATGAAGCTCCAGCCCTTCGCCACCGAGGCCGACGTGGAAGAAGCTCTGAGACT
GTTCCAGGTGTCAACCCTGGACGCCGCCCTGAGCGGAACACTGTCTGGCGTGGAAGGATTCACCAGCCAGGAAGATCAG
GAAATGCTGAGCAGAATCGAGAAACAGCTGAAGAGAAGATTCGCCATCGGAAGCCAGGTGTCCGAGCACAGCATCATCAA
GGACTTCACCAAGCAGAAGTACCCCGAGCACGCCATCCACAAGGTGCTCCAGCTGATGCTGAGAAGAGGCGAGATCCAG
CACCGGATGCAGCGGAAGGTGCTGTACCGGCTGAAATGAGGCGCCGGAATCTTTGCAGGTGCTTACGCTTACTACCTAAA
CTACAATGGTGTTGTCGCTACTAGTGCCGCTTCTTCATCCACTGCATCTGGTGCTTCCGCTTCCGTTACCGGTTCTAAGAA
ACTTCAGAAACTTCAGAAACCAGTGCTGCCCCTAAGACATACACTACTGCCACTGTTACTCAATGTGATGACAATGGTTGTA
ACGTCAAGATAATCACCTCTCAAATACCTGAAGCTACTTCAACCGTCACCGCAACTAGTGCTTCTCCAAAGTCATACACTAC
TGTCACTTCTGAGGGTTCTAAAGCAACCTCATTGAGTTCTCACTGCCCGCTTTCCAGTCGGGAAACCTGTCGTGCCAGCTG
CATTAATGAATCGGCCAACGCGCGGGGAGAGGCGGTTTGCGTATTGGGCGCTCTTCCGCTTCCTCGCTCACTGACTCGCT
GCGCTCGGTCGTTCGGCTGCGGCGAGCGGTATCAGCTCACTCAAAGGCGGTAATACGGTTATCCACAGAATCAGGGGAT
AACGCAGGACAGAACATGTGAGCAGAAGGCCAGCACGAGGCCAGGAGCCGTAAGAAGGCCGCGTTGCTGGCGTTTTTCC
ATAGGCTCCGCCCCCTCGCGAGCTGAGGACTTAAGTGCTGAGGTCGCGAGCATTCAGAAGAGCCATTCAAGGGATCGAT
GTGGTCGCACGACTACGCATCCCTCTGGGCCTTACATAGCCGGATACAGTGACTTATCGATACGCGTGTTAACCTCGAGG
CGGCCGCCATGGTGACAGGACTTGCATAGCTGCGTGCGGGGGAAGGAACTCTTGCGTGTGAGTATGTAGACCCCTGTAC
TACGGATGCGGGCAGAAGATGTGGGCAGAGACACCCGCGTCAAGTTCTCGACCTTCCCGTGGGAGGTGTTCCAGTCCGC
CATACGACCATACCGTTCGGGCATGGCACTATGTACGCGATATCGCTAGCGTTTGCGGGGCACAGCAATATGCAGGCATG

CAAGCTTGGCGTAATCATGGTCATAGCTGTTTCCTGTGTGAAATTGTTATCCGCTCACAATTCCACACAACATACGAGCCG
GAAGCATAAAGTGTAAAGCCTGGGGTGCCTAATGAGTGAGCTAACTCACATTAATTGCGTTGCGCTCACTGCCCGCTTTCC
AGTCGGGAAACCTGTCGTGCCAGCTGCATTAATGAATCGGCCAACGCGCGGGGAGAGGCGGTTTGCGTATTGGGCGCTC
TTCCGCTTCCTCGCTCACTGACTCGCTGCGCTCGGTCGTTCGGCTGCGGCGAGCGGTATCAGCTCACTCAAAGGCGGTAA
TACGGTTATCCACAGAATCAGGGGATAACGCAGGAAAGAACATGTGAGCAAAAGGCCAGCAAAAGGCCAGGAACCGTAAA
AAGGCCGCGTTGCTGGCGTTTTTCCATAGGCTCCGCCCCCCTGACGAGCATCACAAAAATCGACGCTCAAGTCAGAGGTG
GCGAAACCCGACAGGACTATAAAGATACCAGGCGTTTCCCCCTGGAAGCTCCCTCGTGCGCTCTCCTGTTCCGACCCTGC
CGCTTACCGGATACCTGTCCGCCTTTCTCCCTTCGGGAAGCGTGGCGCTTTCTCATAGCTCACGCTGTAGGTATCTCAGTT
CGGTGTAGGTCGTTCGCTCCAAGCTGGGCTGTGTGCACGAACCCCCCGTTCAGCCCGACCGCTGCGCCTTATCCGGTAA
CTATCGTCTTGAGTCCAACCCGGTAAGACACGACTTATCGCCACTGGCAGCAGCCACTGGTAACAGGATTAGCAGAGCGA
GGTATGTAGGCGGTGCTACAGAGTTCTTGAAGTGGTGGCCTAACTACGGCTACACTAGAAGAACAGTATTTGGTATCTGCG
CTCTGCTGAAGCCAGTTACCTTCGGAAAAAGAGTTGGTAGCTCTTGATCCGGCAAACAAACCACCGCTGGTAGCGGTGGT
TTTTTTGTTTGCAAGCAGCAGATTACGCGCAGAAAAAAAGGATCTCAAGAAGATCCTTTGATCTTTTCTACGGGGTCTGACG
CTCAGTGGAACGAAAACTCACGTTAAGGGATTTTGGTCATGAGATTATCAAAAAGGATCTTCACCTAGATCCTTTTAAATTA
AAAATGAAGTTTTAAATCAATCTAAAGTATATATGAGTAAACTTGGTCTGACAGTTACCAATGCTTAATCAGTGAGGCACCTA
TCTCAGCGATCTGTCTATTTCGTTCATCCATAGTTGCCTGACTCCCCGTCGTGTAGATAACTACGATACGGGAGGGCTTAC
CATCTGGCCCCAGTGCTGCAATGATACCGCGAGACCCACGCTCACCGGCTCCAGATTTATCAGCAATAAACCAGCCAGCC
GGAAGGGCCGAGCGCAGAAGTGGTCCTGCAACTTTATCCGCCTCCATCCAGTCTATTAATTGTTGCCGGGAAGCTAGAGT
AAGTAGTTCGCCAGTTAATAGTTTGCGCAACGTTGTTGCCATTGCTACAGGCATCGTGGTGTCACGCTCGTCGTTTGGTAT
GGCTTCATTCAGCTCCGGTTCCCAACGATCAAGGCGAGTTACATGATCCCCCATGTTGTGCAAAAAAGCGGTTAGCTCCTT
CGGTCCTCCGATCGTTGTCAGAAGTAAGTTGGCCGCAGTGTTATCACTCATGGTTATGGCAGCACTGCATAATTCTCTTAC
TGTCATGCCATCCGTAAGATGCTTTTCTGTGACTGGTGAGTACTCAACCAAGTCATTCTGAGAATAGTGTATGCGGCGACC
GAGTTGCTCTTGCCCGGCGTCAATACGGGATAATACCGCGCCACATAGCAGAACTTTAAAAGTGCTCATCATTGGAAAACG
TTCTTCGGGGCGAAAACTCTCAAGGATCTTACCGCTGTTGAGATCCAGTTCGATGTAACCCACTCGTGCACCCAACTGATC
TTCAGCATCTTTTACTTTCACCAGCGTTTCTGGGTGAGCAAAAACAGGAAGGCAAAATGCCGCAAAAAAGGGAATAAGGGC
GACACGGAAATGTTGAATACTCATACTCTTCCTTTTTCAATATTATTGAAGCATTTATCAGGGTTATTGTCTCATGAGCGGAT
ACATATTTGAATGTATTTAGAAAAATAAACAAATAGGGGTTCCGCGCACATTTCCCCGAAAAGTGCCACCTGACGTCTAAGA
AACCATTATTATCATGACATTAACCTATAAAAATAGGCGTATCACGAGGCCCTTTCG

Control for 60 TTAGGG

GTTTGCAAGCAGCAGATTACGCGCAGAAAAAAAGGATCTCAAGAAGATCCTTTGATCTTTTCTACGGGGTCTGACGCTCAG
TGGAACGAAAACTCACGTTAAGGGATTTTGGTCATGAGATTATCAAAAAGGATCTTCACCTAGATCCTTTTAAATTAAAAATG
AAGTTTTAAATCAATCTAAAGTATATATGAGTAAACTTGGTCTGACAGTTACCAATGCTTAATCAGTGAGGCACCTATCTCAG
CGATCTGTCTATTTCGTTCATCCATAGTTGCCTGACTCCCCGTCGTGTAGATAACTACGATACGGGAGGGCTTACCATCTG
GCCCCAGTGCTGCAATGATACCGCGAGACCCACGCTCACCGGCTCCAGATTTATCAGCAATAAACCAGCCAGCCGGAAG
GGCCGAGCGCAGAAGTGGTCCTGCAACTTTATCCGCCTCCATCCAGTCTATTAATTGTTGCCGGGAAGCTAGAGTAAGTA
GTTCGCCAGTTAATAGTTTGCGCAACGTTGTTGCCATTGCTACAGGCATCGTGGTGTCACGCTCGTCGTTTGGTATGGCTT
CATTCAGCTCCGGTTCCCAACGATCAAGGCGAGTTACATGATCCCCCATGTTGTGCAAAAAAGCGGTTAGCTCCTTCGGTC
CTCCGATCGTTGTCAGAAGTAAGTTGGCCGCAGTGTTATCACTCATGGTTATGGCAGCACTGCATAATTCTCTTACTGTCAT
GCCATCCGTAAGATGCTTTTCTGTGACTGGTGAGTACTCAACCAAGTCATTCTGAGAATAGTGTATGCGGCGACCGAGTTG
CTCTTGCCCGGCGTCAATACGGGATAATACCGCGCCACATAGCAGAACTTTAAAAGTGCTCATCATTGGAAAACGTTCTTC
GGGGCGAAAACTCTCAAGGATCTTACCGCTGTTGAGATCCAGTTCGATGTAACCCACTCGTGCACCCAACTGATCTTCAGC
ATCTTTTACTTTCACCAGCGTTTCTGGGTGAGCAAAAACAGGAAGGCAAAATGCCGCAAAAAAGGGAATAAGGGCGACAC
GGAAATGTTGAATACTCATACTCTTCCTTTTTCAATATTATTGAAGCATTTATCAGGGTTATTGTCTCATGAGCGGATACATA
TTTGAATGTATTTAGAAAAATAAACAAATAGGGGTTCCGCGCACATTTCCCCGAAAAGTGCCACCTGACGTCTAAGAAACCA
TTATTATCATGACATTAACCTATAAAAATAGGCGTATCACGAGGCCCTTTCCCTCTGACACATGCAGCTCCCGGACCTCTAG
CCCTGCCCAGCGGAGAAGAGGCAACGACCCTCTGACAAGCAGCCCCGGCAGAAGCAGCAGAAGAACCGACGCCCTGAC
CAGCTCCCCTGGCAGAGATCTGCCCCCATTCGAGGATGAGAGCGAGGGCCTGCTGGGCACAGAGGGACCTCTGGAAGAA
GAAGAGGACGGCGAGGAACTGATCGGCGACGGCATGGAACGGGACTACAGAGCCATCCCCGAGCTGGATGCCTATGAG

GCCGAAGGCCTGGCCCTGGACGACGAGGATGTGGAAGAACTGACCGCCAGCCAGAGAGAGGCCGCCGAGAGAGCTATG
CGGCAGAGAGACAGAGAGGCTGGCAGAGGCCTGGGCAGAATGCGGAGAGGCCTGCTGTACGACAGCGACGAGGAAGAT
GAGGAAAGACCCGCCAGAAAGCGGAGACAGGTGGAAAGAGCCACCGAGGACGGGGAAGAGGATGAAGAGATGATCGAG
AGCATCGAGAACCTGGAAGATCTGAAGGGCCACAGCGTGCGCGAGTGGGTGTCAATGGCTGGCCCCAGACTGGAAATCC
ACCACCGGTTCAAGAACTTTCTGCGGACCCACGTGGACAGCCACGGCCACAACGTGTTCAAAGAACGGATCAGCGACATG
TGCAAAGAGAACCGCGAGAGCCTGGTCGTGAACTACGAGGATCTGGCCGCCAGAGAACACGTGCTGGCCTACTTTCTGC
CTGAGGCCCCTGCCGAGCTGCTCCAGATCTTTGATGAGGCCGCCCTGGAAGTGGTGCTGGCCATGTACCCTAAGTACGA
CCGGATCACCAACCACATCCACGTGCGGATCAGCCATCTGCCCCTGGTGGAAGAACTGCGGAGCCTGAGACAGCTGCAC
CTGAACCAGCTGATCCGGACCTCCGGCGTCGTGACCTCTTGTACAGGCGTGCTGCCCCAGCTGTCTATGGTCAAGTACAA
CTGCAACAAGTGCAACTTCGTGCTGGGACCCTTCTGCCAGAGCCAGAACCAGGAAGTGAAGCCCGGCAGCTGTCCCGAG
TGTCAGAGCGCCGGACCCTTCGAAGTGAACATGGAAGAGACAATCTACCAGAACTACCAGCGCATCCGCATAATACGACT
CACTATAGGCATATGCGGTGTGAAATACCGCACAGATGCGTAAGGAGAAAATACCGCATCAGGCGCCATTCGCCATTCAG
GCTGCGCAACTGTTGGGAAGGGCGATCGGTGCGGGCCTCTTCGCTATTACGCCAGCTGGCGAAAGGGGGATGTGCTGCA
AGGCGATTAAGTTGGGTAACGCCAGGGTTTTCCCAGTCACGACGTTGTAAAACGACGGCCAGTGAATTCGTCCTGAAGGC
TAAGCTGCAAGAGGCCATGAAGCTGCGCCGCTTCGAAGAAAGGCAGAAGCGTCAGGCTCTGTTCAAGCTGGACAACGAG
GACGGTTTCGAAGAAGAAGAAGAGGAAGAGGAAGAAATGACCGACGAGTCCGAAGAGGACGGCGAGGTACTTGCGCAGA
CGCATCTGACGCTTGAGCTTCTCGGAACGCTTCAGCAATTCTTCTTCGTCGTCATCCTCGTGATCTTCCAGAGCCAAGTCG
TTGCCAGAGTCGCTGTGCTCGTCCTCATCAGAGTCGAACTCGTTGTCGTTGGACACCAGACGGAAGTCGGAGGACAGCAG
GAACTCGGCAGTCTCTTGATTGCGATGAGGCTGAGGTGTCCGGTTCTGACGTCGGTTCAGAGGACGAGTACGATGGCGA
AGAAATTGACGAATACGAGGAAGATGTGATCGACGAGGTGCTGCCCAATTGGAGGAACGAGTCATTCTGAGAATAGTGTA
TGCGGCGACCGAGTTGCTCTTGCCCGGCGTCAATACGGGATAGTACCGCGCCACATAGCAGCACTTTGAAAGTGCTCATC
ATTGGAAAACGTTCTTCGGGGCGAAAACTCTCAAGGATCTTACCGCTGTTGAGATCCAGTTCGATGTAACCCACTCGTGCA
CCCAACTGATCTTCAGCATCTTTTACTTTCACCAGCGTTTCTGGGTGAGCAAGAACAGGAAGGCACAATGCCGCAGACAAG
GGAATAAGGGCGACACGGAAATGTTGAATACTCATACTCTTCCTGCTTCAATAGTATTGAAGCATCTATCAGGGTTAGTGTC
TCATGAGCGGATACATATCTGAATGTATGTAGAACACTAGACACATAGGGGTTCCGCGCACATTTCCCCGAGAAGTGCCAC
CTGACGTCTAAGACACCAGTATCATCATGACATTGACCTCTAGACGGATCCGGGTCGACCGCCACCATGAGCGGCTTTGA
CGACCCCGGCATCTTCTACAGCGACAGCTTCGGCGGAGATGCCCAGGCCGATGAAGGCCAGGCCAGAAAGTCTCAGCTC
CAGCGGCGGTTCAAAGAGTTCCTGCGGCAGTACAGAGTGGGCACCGACAGAACCGGCTTCACCTTCAAGTACCGGGACG
AGCTGAAGCGGCACTACAACCTGGGCGAGTACTGGATCGAGGTGGAAATGGAAGATCTGGCCAGCTTCGACGAGGACCT
GGCCGACTACCTGTACAAGCAGCCCGCCGAGCATCTCCAGCTGCTGGAAGAGGCCGCTAAAGAGGTGGCCGACGAAGTG
ACCAGACCCAGGCCTTCTGGCGAGGAAGTGCTCCAGGACATCCAAGTGATGCTGAAGTCCGACGCCAGCCCCAGCAGCA
TCAGATCCCTGAAGTCTGACATGATGAGCCACCTCGTGAAGATCCCCGGAATCATCATTGCCGCCTCTGCCGTGCGGGCC
AAGGCCACCAGAATCAGCATCCAGTGCAGAAGCTGCCGGAACACCCTGACCAATATCGCCATGCGCCCTGGCCTGGAAG
GCTACGCCCTGCCCAGAAAGTGCAACACCGACCAGGCTGGCAGACCCAAGTGCCCCCTGGACCCCTACTTCATCATGCC
CGACAAGTGCAAATGCGTGGACTTCCAGACCCTGAAACTCCAGGAACTGCCCGACGCCGTGCCTCACGGCGAAATGCCT
AGACACATGCAGCTGTACTGCGACAGATACCTGTGCGACAAGGTGGTGCCCGGCAACAGAGTGACCATCATGGGCATCTA
CAGCATCAAGAAGTTCGGCCTGACCACCAGCCGGGGCAGAGATAGAGTGGGCGTGGGCATCCGGTCCAGCTACATCAGA
GTGCTGGGCATCCAGGTGGACACCGATGGCAGCGGCAGATCCTTTGCTGGGGCCGTGAGCCCCCAGGAAGAGGAAGAG
TTTAGACGGCTGGCCGCCCTGCCTAACGTGTACGAAGTGATCAGCAAGTCTATCGCCCCCAGCATCTTCGGCGGCACCGA
TATGAAGAAGGCCATTGCCTGCCTGCTGTTCGGCGGCTCCAGAAAGAGACTGCCTGACGGCCTGACAAGACGGGGCGAC
ATCAACCTGCTGATGCTGGGCGATCCTGGCACCGCCAAATCACAGCTGCTGAAGTTCGTGGAAAAGTGCAGCCCCATCGG
CGTGTACACCAGCGGCAAGGGATCTTCTGCCGCCGGACTGACAGCCAGCGTGATGAGAGATCCCAGCAGCCGGAATTTC
ATCATGGAAGGCGGCGCTATGGTGCTGGCCGATGGCGGAGTCGTGTGCATCGACGAGTTCGACAAGATGCGCGAGGAC
GACCGGGTGGCCATTCACGAGGCTATGGAACAGCAGACCATCTCTATCGCCAAGGCCGGAATCACCACCACCCTGAACA
GCCGGTGTAGCGTGCTGGCTGCCGCCAATAGCGTGTTCGGCAGATGGGACGAGACAAAGGGCGAGGACAACATCGACTT
CATGCCCACCATCCTGAGCCGCTTCGACATGATCTTCATCGTGAAGGACGAGCACAACGAGGAACGGGACGTGATGCTG
GCCAAGCACGTGATCACCCTGCACGTGTCCGCCCTGACACAGACACAGGCCGTGGAAGGCGAGATCGACCTGGCCAAAC
TGAAGAAGTTTATCGCCTACTGTCGCGTGAAGTGCGGCCCCAGACTGTCTGCCGAGGCCGCCGAGAAGCTGAAGAACCG
GTACATCATCATGCGGAGCGGAGCCCGGCAGCACGAGAGAGACAGCGATCGGAGAAGCAGCATCCCCATCACCGTGCG
GCAGCTGGAAGCCATCGTGCGGATTGCCGAAGCCCTGAGCAAGATGAAGCTCCAGCCCTTCGCCACCGAGGCCGACGTG

GAAGAAGCTCTGAGACTGTTCCAGGTGTCAACCCTGGACGCCGCCCTGAGCGGAACACTGTCTGGCGTGGAAGGATTCA
CCAGCCAGGAAGATCAGGAAATGCTGAGCAGAATCGAGAAACAGCTGAAGAGAAGATTCGCCATCGGAAGCCAGGTGTC
CGAGCACAGCATCATCAAGGACTTCACCAAGCAGAAGTACCCCGAGCACGCCATCCACAAGGTGCTCCAGCTGATGCTGA
GAAGAGGCGAGATCCAGCACCGGATGCAGCGGAAGGTGCTGTACCGGCTGAAATGAGGCGCCGGAATCTTTGCAGGTGC
TTACGCTTACTACCTAAACTACAATGGTGTTGTCGCTACTAGTGCCGCTTCTTCATCCACTGCATCTGGTGCTTCCGCTTCC
GTTACCGGTTCTAAGAAACTTCAGAAACTTCAGAAACCAGTGCTGCCCCTAAGACATACACTACTGCCACTGTTACTCAATG
TGATGACAATGGTTGTAACGTCAAGATAATCACCTCTCAAATACCTGAAGCTACTTCAACCGTCACCGCAACTAGTGCTTCT
CCAAAGTCATACACTACTGTCACTTCTGAGGGTTCTAAAGCAACCTCATTGAGTTCTCACTGCCCGCTTTCCAGTCGGGAA
ACCTGTCGTGCCAGCTGCATTAATTAATCGGCCAACGCGCGGGGAGAGGCGGTTTGCGTATTGGGCGCTCTTCCGCTTCC
TCGCTCACTGACTCGCTGCGCTCGGTCGTTCGGCTGCGGCGAGCGGTATCAGCTCACTCAAAGGCGGTAATACGGTTATC
CACAGAATCAGGGGATAACGCAGGACAGAACATGTGAGCAGAAGGCCAGCACGAGGCCAGGAGCCGTAAGAAGGCCGC
GTTGCTGGCGTTTTTCCATAGGCTCCGCCCCCTCGCGAGCTGAGGACTTAAGTGCTGAGGTCGCGAGCATTCAGAAGAGC
CATTCAAGGGATCGATGTGGTCGCACGACTACGCATCCCTCTGGGCCTTACATAGCCGGATACAGTGACTTATCGATACG
CGTGATGCCTTGCCTGCAGAGCCGCGTGTTAACCTCGAGGCGGCCGCCATGGTGACAGGACTTGCATAGCTGCGTGCGG
GGGAAGGAACTCTTGCGTGTGAGTATGTAGACCCCTGTACTACGGATGCGGGCAGAAGATGTGGGCAGAGACACCCGCG
TCAAGTTCTCGACCTTCCCGTGGGAGGTGTTCCAGTCCGCCATACGACCATACCGTTCGGGCATGGCACTATGTACGCGA
TATCGCTAGCGTTTGCGGGGCACAGCAATATGCAGGCATGCAAGCTTGGCGTAATCATGGTCATAGCTGTTTCCTGTGTGA
AATTGTTATCCGCTCACAATTCCACACAACATACGAGCCGGAAGCATAAAGTGTAAAGCCTGGGGTGCCTAATGAGTGAGC
TAACTCACATTAATTGCGTTGCGCTCACTGCCCGCTTTCCAGTCGGGAAACCTGTCGTGCCAGCTGCATTAATGAATCGGC
CAACGCGCGGGGAGAGGCGGTTTGCGTATTGGGCGCTCTTCCGCTTCCTCGCTCACTGACTCGCTGCGCTCGGTCGTTC
GGCTGCGGCGAGCGGTATCAGCTCACTCAAAGGCGGTAATACGGTTATCCACAGAATCAGGGGATAACGCAGGAAAGAA
CATGTGAGCAAAAGGCCAGCAAAAGGCCAGGAACCGTAAAAAGGCCGCGTTGCTGGCGTTTTTCCATAGGCTCCGCCCC
CCTGACGAGCATCACAAAAATCGACGCTCAAGTCAGAGGTGGCGAAACCCGACAGGACTATAAAGATACCAGGCGTTTCC
CCCTGGAAGCTCCCTCGTGCGCTCTCCTGTTCCGACCCTGCCGCTTACCGGATACCTGTCCGCCTTTCTCCCTTCGGGAA
GCGTGGCGCTTTCTCATAGCTCACGCTGTAGGTATCTCAGTTCGGTGTAGGTCGTTCGCTCCAAGCTGGGCTGTGTGCAC
GAACCCCCCGTTCAGCCCGACCGCTGCGCCTTATCCGGTAACTATCGTCTTGAGTCCAACCCGGTAAGACACGACTTATC
GCCACTGGCAGCAGCCACTGGTAACAGGATTAGCAGAGCGAGGTATGTAGGCGGTGCTACAGAGTTCTTGAAGTGGTGG
CCTAACTACGGCTACACTAGAAGAACAGTATTTGGTATCTGCGCTCTGCTGAAGCCAGTTACCTTCGGAAAAAGAGTTGGT
AGCTCTTGATCCGGCAAACAAACCACCGCTGGTAGCGGTGGTTTTTTT

60 TTAGGG telomeric

CGAAAGGGCCTCGTGATACGCCTATTTTTATAGGTTAATGTCATGATAATAATGGTTTCTTAGACGTCAGGTGGCACTTTTC
GGGGAAATGTGCGCGGAACCCCTATTTGTTTATTTTTCTAAATACATTCAAATATGTATCCGCTCATGAGACAATAACCCTG
ATAAATGCTTCAATAATATTGAAAAAGGAAGAGTATGAGTATTCAACATTTCCGTGTCGCCCTTATTCCCTTTTTTGCGGCAT
TTTGCCTTCCTGTTTTTGCTCACCCAGAAACGCTGGTGAAAGTAAAAGATGCTGAAGATCAGTTGGGTGCACGAGTGGGTT
ACATCGAACTGGATCTCAACAGCGGTAAGATCCTTGAGAGTTTTCGCCCCGAAGAACGTTTTCCAATGATGAGCACTTTTA
AAGTTCTGCTATGTGGCGCGGTATTATCCCGTATTGACGCCGGGCAAGAGCAACTCGGTCGCCGCATACACTATTCTCAG
AATGACTTGGTTGAGTACTCACCAGTCACAGAAAAGCATCTTACGGATGGCATGACAGTAAGAGAATTATGCAGTGCTGCC
ATAACCATGAGTGATAACACTGCGGCCAACTTACTTCTGACAACGATCGGAGGACCGAAGGAGCTAACCGCTTTTTTGCAC
AACATGGGGGATCATGTAACTCGCCTTGATCGTTGGGAACCGGAGCTGAATGAAGCCATACCAAACGACGAGCGTGACAC
CACGATGCCTGTAGCAATGGCAACAACGTTGCGCAAACTATTAACTGGCGAACTACTTACTCTAGCTTCCCGGCAACAATT
AATAGACTGGATGGAGGCGGATAAAGTTGCAGGACCACTTCTGCGCTCGGCCCTTCCGGCTGGCTGGTTTATTGCTGATA
AATCTGGAGCCGGTGAGCGTGGGTCTCGCGGTATCATTGCAGCACTGGGGCCAGATGGTAAGCCCTCCCGTATCGTAGT
TATCTACACGACGGGGAGTCAGGCAACTATGGATGAACGAAATAGACAGATCGCTGAGATAGGTGCCTCACTGATTAAGC
ATTGGTAACTGTCAGACCAAGTTTACTCATATATACTTTAGATTGATTTAAAACTTCATTTTTAATTTAAAAGGATCTAGGTGA
AGATCCTTTTTGATAATCTCATGACCAAAATCCCTTAACGTGAGTTTTCGTTCCACTGAGCGTCAGACCCCGTAGAAAAGAT
CAAAGGATCTTCTTGAGATCCTTTTTTTCTGCGCGTAATCTGCTGCTTGCAAACAAAAAAACCACCGCTACCAGCGGTGGTT
TGTTTGCCGGATCAAGAGCTACCAACTCTTTTTCCGAAGGTAACTGGCTTCAGCAGAGCGCAGATACCAAATACTGTTCTT
CTAGTGTAGCCGTAGTTAGGCCACCACTTCAAGAACTCTGTAGCACCGCCTACATACCTCGCTCTGCTAATCCTGTTACCA

GTGGCTGCTGCCAGTGGCGATAAGTCGTGTCTTACCGGGTTGGACTCAAGACGATAGTTACCGGATAAGGCGCAGCGGT
CGGGCTGAACGGGGGGTTCGTGCACACAGCCCAGCTTGGAGCGAACGACCTACACCGAACTGAGATACCTACAGCGTGA
GCTATGAGAAAGCGCCACGCTTCCCGAAGGGAGAAAGGCGGACAGGTATCCGGTAAGCGGCAGGGTCGGAACAGGAGA
GCGCACGAGGGAGCTTCCAGGGGGAAACGCCTGGTATCTTTATAGTCCTGTCGGGTTTCGCCACCTCTGACTTGAGCGTC
GATTTTTGTGATGCTCGTCAGGGGGGCGGAGCCTATGGAAAAACGCCAGCAACGCGGCCTTTTTACGGTTCCTGGCCTTT
TGCTGGCCTTTTGCTCACATGTTCTTTCCTGCGTTATCCCCTGATTCTGTGGATAACCGTATTACCGCCTTTGAGTGAGCTG
ATACCGCTCGCCGCAGCCGAACGACCGAGCGCAGCGAGTCAGTGAGCGAGGAAGCGGAAGAGCGCCCAATACGCAAAC
CGCCTCTCCCCGCGCGTTGGCCGATTCATTAATGCAGCTGGCACGACAGGTTTCCCGACTGGAAAGCGGGCAGTGAGCG
CAACGCAATTAATGTGAGTTAGCTCACTCATTAGGCACCCCAGGCTTTACACTTTATGCTTCCGGCTCGTATGTTGTGTGGA
ATTGTGAGCGGATAACAATTTCACACAGGAAACAGCTATGACCATGATTACGCCAAGCTTGCATGCCTGCATATTGCTGTG
CCCCGCAAACGCTAGCGATATCGCGTACATAGTGCCATGCCCGAACGGTATGGTCGTATGGCGGACTGGAACACCTCCCA
CGGGAAGGTCGAGAACTTGACGCGGGTGTCTCTGCCCACATCTTCTGCCCGCATCCGTAGTACAGGGGTCTACATACTCA
CACGCAAGAGTTCCTTCCCCCGCACGCAGCTATGCAAGTCCTGTCACCATGGCGGCCGCCTCGAGGTTAACACGCGGCT
CTGCAGGCAAGGCATCACGCGTATCGATAAGTCACTGTATCCGGCTATGTAAGGCCCAGAGGGATGCGTAGTCGTGCGAC
CACATCGATCCCTTGAATGGCTCTTCTGAATGCTCGCGACCTCAGCACTTAAGTCCTCAGCTCGCGAGGGGGCGGAGCCT
ATGGAAAAACGCCAGCAACGCGGCCTTCTTACGGCTCCTGGCCTCGTGCTGGCCTTCTGCTCACATGTTCTGTCCTGCGT
TATCCCCTGATTCTGTGGATAACCGTATTACCGCCTTTGAGTGAGCTGATACCGCTCGCCGCAGCCGAACGACCGAGCGC
AGCGAGTCAGTGAGCGAGGAAGCGGAAGAGCGCCCAATACGCAAACCGCCTCTCCCCGCGCGTTGGCCGATTCATTAAT
GCAGCTGGCACGACAGGTTTCCCGACTGGAAAGCGGGCAGTGAGAACTCAATGAGGTTGCTTTAGAACCCTCAGAAGTGA
CAGTAGTGTATGACTTTGGAGAAGCACTAGTTGCGGTGACGGTTGAAGTAGCTTCAGGTATTTGAGAGGTGATTATCTTGA
CGTTACAACCATTGTCATCACATTGAGTAACAGTGGCAGTAGTGTATGTCTTAGGGGCAGCACTGGTTTCTGAAGTTTCTG
AAGTTTCTTAGAACCGGTAACGGAAGCGGAAGCACCAGATGCAGTGGATGAAGAAGCGGCACTAGTAGCGACAACACCAT
TGTAGTTTAGGTAGTAAGCGTAAGCACCTGCAAAGATTCCGGCGCCTCATTTCAGCCGGTACAGCACCTTCCGCTGCATCC
GGTGCTGGATCTCGCCTCTTCTCAGCATCAGCTGGAGCACCTTGTGGATGGCGTGCTCGGGGTACTTCTGCTTGGTGAAG
TCCTTGATGATGCTGTGCTCGGACACCTGGCTTCCGATGGCGAATCTTCTCTTCAGCTGTTTCTCGATTCTGCTCAGCATTT
CCTGATCTTCCTGGCTGGTGAATCCTTCCACGCCAGACAGTGTTCCGCTCAGGGCGGCGTCCAGGGTTGACACCTGGAAC
AGTCTCAGAGCTTCTTCCACGTCGGCCTCGGTGGCGAAGGGCTGGAGCTTCATCTTGCTCAGGGCTTCGGCAATCCGCAC
GATGGCTTCCAGCTGCCGCACGGTGATGGGGATGCTGCTTCTCCGATCGCTGTCTCTCTCGTGCTGCCGGGCTCCGCTC
CGCATGATGATGTACCGGTTCTTCAGCTTCTCGGCGGCCTCGGCAGACAGTCTGGGGCCGCACTTCACGCGACAGTAGG
CGATAAACTTCTTCAGTTTGGCCAGGTCGATCTCGCCTTCCACGGCCTGTGTCTGTGTCAGGGCGGACACGTGCAGGGTG
ATCACGTGCTTGGCCAGCATCACGTCCCGTTCCTCGTTGTGCTCGTCCTTCACGATGAAGATCATGTCGAAGCGGCTCAG
GATGGTGGGCATGAAGTCGATGTTGTCCTCGCCCTTTGTCTCGTCCCATCTGCCGAACACGCTATTGGCGGCAGCCAGCA
CGCTACACCGGCTGTTCAGGGTGGTGGTGATTCCGGCCTTGGCGATAGAGATGGTCTGCTGTTCCATAGCCTCGTGAATG
GCCACCCGGTCGTCCTCGCGCATCTTGTCGAACTCGTCGATGCACACGACTCCGCCATCGGCCAGCACCATAGCGCCGC
CTTCCATGATGAAATTCCGGCTGCTGGGATCTCTCATCACGCTGGCTGTCAGTCCGGCGGCAGAAGATCCCTTGCCGCTG
GTGTACACGCCGATGGGGCTGCACTTTTCCACGAACTTCAGCAGCTGTGATTTGGCGGTGCCAGGATCGCCCAGCATCAG
CAGGTTGATGTCGCCCCGTCTTGTCAGGCCGTCAGGCAGTCTCTTTCTGGAGCCGCCGAACAGCAGGCAGGCAATGGCC
TTCTTCATATCGGTGCCGCCGAAGATGCTGGGGGCGATAGACTTGCTGATCACTTCGTACACGTTAGGCAGGGCGGCCAG
CCGTCTAAACTCTTCCTCTTCCTGGGGGCTCACGGCCCCAGCAAAGGATCTGCCGCTGCCATCGGTGTCCACCTGGATGC
CCAGCACTCTGATGTAGCTGGACCGGATGCCCACGCCCACTCTATCTCTGCCCCGGCTGGTGGTCAGGCCGAACTTCTTG
ATGCTGTAGATGCCCATGATGGTCACTCTGTTGCCGGGCACCACCTTGTCGCACAGGTATCTGTCGCAGTACAGCTGCAT
GTGTCTAGGCATTTCGCCGTGAGGCACGGCGTCGGGCAGTTCCTGGAGTTTCAGGGTCTGGAAGTCCACGCATTTGCACT
TGTCGGGCATGATGAAGTAGGGGTCCAGGGGGCACTTGGGTCTGCCAGCCTGGTCGGTGTTGCACTTTCTGGGCAGGGC
GTAGCCTTCCAGGCCAGGGCGCATGGCGATATTGGTCAGGGTGTTCCGGCAGCTTCTGCACTGGATGCTGATTCTGGTG
GCCTTGGCCCGCACGGCAGAGGCGGCAATGATGATTCCGGGGATCTTCACGAGGTGGCTCATCATGTCAGACTTCAGGG
ATCTGATGCTGCTGGGGCTGGCGTCGGACTTCAGCATCACTTGGATGTCCTGGAGCACTTCCTCGCCAGAAGGCCTGGGT
CTGGTCACTTCGTCGGCCACCTCTTTAGCGGCCTCTTCCAGCAGCTGGAGATGCTCGGCGGGCTGCTTGTACAGGTAGTC
GGCCAGGTCCTCGTCGAAGCTGGCCAGATCTTCCATTTCCACCTCGATCCAGTACTCGCCCAGGTTGTAGTGCCGCTTCA
GCTCGTCCCGGTACTTGAAGGTGAAGCCGGTTCTGTCGGTGCCCACTCTGTACTGCCGCAGGAACTCTTTGAACCGCCGC
TGGAGCTGAGACTTTCTGGCCTGGCCTTCATCGGCCTGGGCATCTCCGCCGAAGCTGTCGCTGTAGAAGATGCCGGGGT

CGTCAAAGCCGCTCATGGTGGCGGTCGACCCGGATCcGTCTAGAGGTCAATGTCATGATGATACTGGTGTCTTAGACGTC
AGGTGGCACTTCTCGGGGAAATGTGCGCGGAACCCCTATGTGTCTAGTGTTCTACATACATTCAGATATGTATCCGCTCAT
GAGACACTAACCCTGATAGATGCTTCAATACTATTGAAGCAGGAAGAGTATGAGTATTCAACATTTCCGTGTCGCCCTTATT
CCCTTGTCTGCGGCATTGTGCCTTCCTGTTCTTGCTCACCCAGAAACGCTGGTGAAAGTAAAAGATGCTGAAGATCAGTTG
GGTGCACGAGTGGGTTACATCGAACTGGATCTCAACAGCGGTAAGATCCTTGAGAGTTTTCGCCCCGAAGAACGTTTTCC
AATGATGAGCACTTTCAAAGTGCTGCTATGTGGCGCGGTACTATCCCGTATTGACGCCGGGCAAGAGCAACTCGGTCGCC
GCATACACTATTCTCAGAATGACTCGTGGTTGCAATTGAGCGGGGATCCTCTAGAGTCGACGAAGACTTAGGGTTAGGGTT
AGGGTTAGGGTTAGGGTTAGGGTTAGGGTTAGGGTTAGGGTTAGGGTTAGGGTTAGGGTTAGGGTTAGGGTTAGGGTTAG
GGTTAGGGTTAGGGTTAGGGTTAGGGTTAGGGTTAGGGTTAGGGTTAGGGTTAGGGTTAGGGTTAGGGTTAGGGTTAGG
GTTAGGGTTAGGGTTAGGGTTAGGGTTAGGGTTAGGGTTAGGGTTAGGGTTAGGGTTAGGGTTAGGGTTAGGGTTAGGGT
TAGGGTTAGGGTTAGGGTTAGGGTTAGGGTTAGGGTTAGGGTTAGGGTTAGGGTTAGGGTTAGGGTTAGGGTTAGGGTTA
GGGTTAGGGTTAGGGTTAGGGTTAGGGCGAGACGTGTCCCGTCGACCTGCATAGCGGGTACCGAGCTCGAATTCACTGG
CCGTCGTTTTACAACGTCGTGACTGGGAAAACCCTGGCGTTACCCAACTTAATCGCCTTGCAGCACATCCCCCTTTCGCCA
GCTGGCGTAATAGCGAAGAGGCCCGCACCGATCGCCCTTCCCAACAGTTGCGCAGCCTGAATGGCGAATGGCGCCTGAT
GCGGTATTTTCTCCTTACGCATCTGTGCGGTATTTCACACCGCATATGCCTATAGTGAGTCGTATTATGCGGATGCGCTGG
TAGTTCTGGTAGATTGTCTCTTCCATGTTCACTTCGAAGGGTCCGGCGCTCTGACACTCGGGACAGCTGCCGGGCTTCAC
TTCCTGGTTCTGGCTCTGGCAGAAGGGTCCCAGCACGAAGTTGCACTTGTTGCAGTTGTACTTGACCATAGACAGCTGGG
GCAGCACGCCTGTACAAGAGGTCACGACGCCGGAGGTCCGGATCAGCTGGTTCAGGTGCAGCTGTCTCAGGCTCCGCAG
TTCTTCCACCAGGGGCAGATGGCTGATCCGCACGTGGATGTGGTTGGTGATCCGGTCGTACTTAGGGTACATGGCCAGCA
CCACTTCCAGGGCGGCCTCATCAAAGATCTGGAGCAGCTCGGCAGGGGCCTCAGGCAGAAAGTAGGCCAGCACGTGTTC
TCTGGCGGCCAGATCCTCGTAGTTCACGACCAGGCTCTCGCGGTTCTCTTTGCACATGTCGCTGATCCGTTCTTTGAACAC
GTTGTGGCCGTGGCTGTCCACGTGGGTCCGCAGAAAGTTCTTGAACCGGTGGTGGATTTCCAGTCTGGGGCCAGCCATT
GACACCCACTCGCGCACGCTGTGGCCCTTCAGATCTTCCAGGTTCTCGATGCTCTCGATCATCTCTTCATCCTCTTCCCCG
TCCTCGGTGGCTCTTTCCACCTGTCTCCGCTTTCTGGCGGGTCTTTCCTCATCTTCCTCGTCGCTGTCGTACAGCAGGCCT
CTCCGCATTCTGCCCAGGCCTCTGCCAGCCTCTCTGTCTCTCTGCCGCATAGCTCTCTCGGCGGCCTCTCTCTGGCTGGC
GGTCAGTTCTTCCACATCCTCGTCGTCCAGGGCCAGGCCTTCGGCCTCATAGGCATCCAGCTCGGGGATGGCTCTGTAGT
CCCGTTCCATGCCGTCGCCGATCAGTTCCTCGCCGTCCTCTTCTTCTTCCAGAGGTCCCTCTGTGCCCAGCAGGCCCTCG
CTCTCATCCTCGAATGGGGGCAGATCTCTGCCAGGGGAGCTGGTCAGGGCGTCGGTTCTTCTGCTGCTTCTGCCGGGGC
TGCTTGTCAGAGGGTCGTTGCCTCTTCTCCGCTGGGCAGGGCTAGAGGTCCGGGAGCTGCATGTGTCAGAGG

60 TTAGGG telomeric from Fig. 3C

CCTCTGACACATGCAGCTCCCGGACCTCTAGCCCTGCCCAGCGGAGAAGAGGCAACGACCCTCTGACAAGCAGCCCCGG
CAGAAGCAGCAGAAGAACCGACGCCCTGACCAGCTCCCCTGGCAGAGATCTGCCCCCATTCGAGGATGAGAGCGAGGGC
CTGCTGGGCACAGAGGGACCTCTGGAAGAAGAAGAGGACGGCGAGGAACTGATCGGCGACGGCATGGAACGGGACTAC
AGAGCCATCCCCGAGCTGGATGCCTATGAGGCCGAAGGCCTGGCCCTGGACGACGAGGATGTGGAAGAACTGACCGCC
AGCCAGAGAGAGGCCGCCGAGAGAGCTATGCGGCAGAGAGACAGAGAGGCTGGCAGAGGCCTGGGCAGAATGCGGAG
AGGCCTGCTGTACGACAGCGACGAGGAAGATGAGGAAAGACCCGCCAGAAAGCGGAGACAGGTGGAAAGAGCCACCGA
GGACGGGGAAGAGGATGAAGAGATGATCGAGAGCATCGAGAACCTGGAAGATCTGAAGGGCCACAGCGTGCGCGAGTG
GGTGTCAATGGCTGGCCCCAGACTGGAAATCCACCACCGGTTCAAGAACTTTCTGCGGACCCACGTGGACAGCCACGGC
CACAACGTGTTCAAAGAACGGATCAGCGACATGTGCAAAGAGAACCGCGAGAGCCTGGTCGTGAACTACGAGGATCTGG
CCGCCAGAGAACACGTGCTGGCCTACTTTCTGCCTGAGGCCCCTGCCGAGCTGCTCCAGATCTTTGATGAGGCCGCCCT
GGAAGTGGTGCTGGCCATGTACCCTAAGTACGACCGGATCACCAACCACATCCACGTGCGGATCAGCCATCTGCCCCTG
GTGGAAGAACTGCGGAGCCTGAGACAGCTGCACCTGAACCAGCTGATCCGGACCTCCGGCGTCGTGACCTCTTGTACAG
GCGTGCTGCCCCAGCTGTCTATGGTCAAGTACAACTGCAACAAGTGCAACTTCGTGCTGGGACCCTTCTGCCAGAGCCAG
AACCAGGAAGTGAAGCCCGGCAGCTGTCCCGAGTGTCAGAGCGCCGGACCCCTTCGAAGTGAACATGGAAGAGACAATCT
ACCAGAACTACCAGCGCATCCGCATAATACGACTCACTATAGGCATATGCGGTGTGAAATACCGCACAGATGCGTAAGGA
GAAAATACCGCATCAGGCGCCATTCGCCATTCAGGCTGCGCAACTGTTGGGAAGGGCGATCGGTGCGGGCCTCTTCGCT
ATTACGCCAGCTGGCGAAAGGGGGATGTGCTGCAAGGCGATTAAGTTGGGTAACGCCAGGGTTTTCCCAGTCACGACGTT
GTAAAACGACGGCCAGTGAATTCGAGCTCGGTACCCGGGGATCCTCTAGAGTCGACGAAGACTTAGGGTTAGGGTTAGG

GTTAGGGTTAGGGTTAGGGTTAGGGTTAGGGTTAGGGTTAGGGTTAGGGTTAGGGTTAGGGTTAGGGTTAGGGTTAGGGT
TAGGGTTAGGGTTAGGGTTAGGGTTAGGGTTAGGGTTAGGGTTAGGGTTAGGGTTAGGGTTAGGGTTAGGGTTAGGGTTA
GGGTTAGGGTTAGGGTTAGGGTTAGGGTTAGGGTTAGGGTTAGGGTTAGGGTTAGGGTTAGGGTTAGGGTTAGGGTTAG
GGTTAGGGTTAGGGTTAGGGTTAGGGTTAGGGTTAGGGTTAGGGTTAGGGTTAGGGTTAGGGTTAGGGTTAGGGTTAGG
GTTAGGGTTAGGGTTAGGGTTAGGGCGAGACGTGTCCCGTCGACCTGCATAGCGCTCAATTGCAACCACTGCAGAGTCAT
TCTGAGAATAGTGTATGCGGCGACCGAGTTGCTCTTGCCCGGCGTCAATACGGGATAGTACCGCGCCACATAGCAGCACT
TTGAAAGTGCTCATCATTGGAAAACGTTCTTCGGGGCGAAAACTCTCAAGGATCTTACCGCTGTTGAGATCCAGTTCGATG
TAACCCACTCGTGCACCCAACTGATCTTCAGCATCTTTTACTTTCACCAGCGTTTCTGGGTGAGCAAGAACAGGAAGGCAC
AATGCCGCAGACAAGGGAATAAGGGCGACACGGAAATGTTGAATACTCATACTCTTCCTGCTTCAATAGTATTGAAGCATC
TATCAGGGTTAGTGTCTCATGAGCGGATACATATCTGAATGTATGTAGAACACTAGACACATAGGGGTTCCGCGCACATTT
CCCCGAGAAGTGCCACCTGACGTCTAAGACACCAGTATCATCATGACATTGACCTCTAGACgGATCCGGGTCGACCGCCA
CCATGAGCGGCTTTGACGACCCCGGCATCTTCTACAGCGACAGCTTCGGCGGAGATGCCCAGGCCGATGAAGGCCAGGC
CAGAAAGTCTCAGCTCCAGCGGCGGTTCAAAGAGTTCCTGCGGCAGTACAGAGTGGGCACCGACAGAACCGGCTTCACC
TTCAAGTACCGGGACGAGCTGAAGCGGCACTACAACCTGGGCGAGTACTGGATCGAGGTGGAAATGGAAGATCTGGCCA
GCTTCGACGAGGACCTGGCCGACTACCTGTACAAGCAGCCCGCCGAGCATCTCCAGCTGCTGGAAGAGGCCGCTAAAGA
GGTGGCCGACGAAGTGACCAGACCCAGGCCTTCTGGCGAGGAAGTGCTCCAGGACATCCAAGTGATGCTGAAGTCCGAC
GCCAGCCCCAGCAGCATCAGATCCCTGAAGTCTGACATGATGAGCCACCTCGTGAAGATCCCCGGAATCATCATTGCCGC
CTCTGCCGTGCGGGCCAAGGCCACCAGAATCAGCATCCAGTGCAGAAGCTGCCGGAACACCCTGACCAATATCGCCATG
CGCCCTGGCCTGGAAGGCTACGCCCTGCCCAGAAAGTGCAACACCGACCAGGCTGGCAGACCCAAGTGCCCCCTGGAC
CCCTACTTCATCATGCCCGACAAGTGCAAATGCGTGGACTTCCAGACCCTGAAACTCCAGGAACTGCCCGACGCCGTGCC
TCACGGCGAAATGCCTAGACACATGCAGCTGTACTGCGACAGATACCTGTGCGACAAGGTGGTGCCCGGCAACAGAGTG
ACCATCATGGGCATCTACAGCATCAAGAAGTTCGGCCTGACCACCAGCCGGGGCAGAGATAGAGTGGGCGTGGGCATCC
GGTCCAGCTACATCAGAGTGCTGGGCATCCAGGTGGACACCGATGGCAGCGGCAGATCCTTTGCTGGGGCCGTGAGCCC
CCAGGAAGAGGAAGAGTTTAGACGGCTGGCCGCCCTGCCTAACGTGTACGAAGTGATCAGCAAGTCTATCGCCCCCAGC
ATCTTCGGCGGCACCGATATGAAGAAGGCCATTGCCTGCCTGCTGTTCGGCGGCTCCAGAAAGAGACTGCCTGACGGCC
TGACAAGACGGGGCGACATCAACCTGCTGATGCTGGGCGATCCTGGCACCGCCAAATCACAGCTGCTGAAGTTCGTGGA
AAAGTGCAGCCCCATCGGCGTGTACACCAGCGGCAAGGGATCTTCTGCCGCCGGACTGACAGCCAGCGTGATGAGAGAT
CCCAGCAGCCGGAATTTCATCATGGAAGGCGGCGCTATGGTGCTGGCCGATGGCGGAGTCGTGTGCATCGACGAGTTCG
ACAAGATGCGCGAGGACGACCGGGTGGCCATTCACGAGGCTATGGAACAGCAGACCATCTCTATCGCCAAGGCCGGAAT
CACCACCACCCTGAACAGCCGGTGTAGCGTGCTGGCTGCCGCCAATAGCGTGTTCGGCAGATGGGACGAGACAAAGGGC
GAGGACAACATCGACTTCATGCCCACCATCCTGAGCCGCTTCGACATGATCTTCATCGTGAAGGACGAGCACAACGAGGA
ACGGGACGTGATGCTGGCCAAGCACGTGATCACCCTGCACGTGTCCGCCCTGACACAGACACAGGCCGTGGAAGGCGA
GATCGACCTGGCCAAACTGAAGAAGTTTATCGCCTACTGTCGCGTGAAGTGCGGCCCCAGACTGTCTGCCGAGGCCGCC
GAGAAGCTGAAGAACCGGTACATCATCATGCGGAGCGGAGCCCGGCAGCACGAGAGAGACAGCGATCGGAGAAGCAGC
ATCCCCATCACCGTGCGGCAGCTGGAAGCCATCGTGCGGATTGCCGAAGCCCTGAGCAAGATGAAGCTCCAGCCCTTCG
CCACCGAGGCCGACGTGGAAGAAGCTCTGAGACTGTTCCAGGTGTCAACCCTGGACGCCGCCCTGAGCGGAACACTGTC
TGGCGTGGAAGGATTCACCAGCCAGGAAGATCAGGAAATGCTGAGCAGAATCGAGAAACAGCTGAAGAGAAGATTCGCCA
TCGGAAGCCAGGTGTCCGAGCACAGCATCATCAAGGACTTCACCAAGCAGAAGTACCCCGAGCACGCCATCCACAAGGT
GCTCCAGCTGATGCTGAGAAGAGGCGAGATCCAGCACCGGATGCAGCGGAAGGTGCTGTACCGGCTGAAATGAGGCGC
CGGAATCTTTGCAGGTGCTTACGCTTACTACCTAAACTACAATGGTGTTGTCGCTACTAGTGCCGCTTCTTCATCCACTGCA
TCTGGTGCTTCCGCTTCCGTTACCGGTTCTAAGAAACTTCAGAAACTTCAGAAACCAGTGCTGCCCCTAAGACATACACTA
CTGCCACTGTTACTCAATGTGATGACAATGGTTGTAACGTCAAGATAATCACCTCTCAAATACCTGAAGCTACTTCAACCGT
CACCGCAACTAGTGCTTCTCCAAAGTCATACACTACTGTCACTTCTGAGGGTTCTAAAGCAACCTCATTGAGTTCTCACTGC
CCGCTTTCCAGTCGGGAAACCTGTCGTGCCAGCTGCATTAATGAATCGGCCAACGCGCGGGGAGAGGCGGTTTGCGTAT
TGGGCGCTCTTCCGCTTCCTCGCTCACTGACTCGCTGCGCTCGGTCGTTCGGCTGCGGCGAGCGGTATCAGCTCACTCA
AAGGCGGTAATACGGTTATCCACAGAATCAGGGGATAACGCAGGACAGAACATGTGAGCAGAAGGCCAGCACGAGGCCA
GGAGCCGTAAGAAGGCCGCGTTGCTGGCGTTTTTCCATAGGCTCCGCCCCCTCGCGAGCTGAGGACTTAAGTGCTGAGG
TCGCGAGCATTCAGAAGAGCCATTCAAGGGATCGATGTGGTCGCACGACTACGCATCCCTCTGGGCCTTACATAGCCGGA
TACAGTGACTTATCGATACGCGTGTTAACCTCGAGGCGGCCGCCATGGTGACAGGACTTGCATAGCTGCGTGCGGGGGA
AGGAACTCTTGCGTGTGAGTATGTAGACCCCTGTACTACGGATGCGGGCAGAAGATGTGGGCAGAGACACCCGCGTCAA

GTTCTCGACCTTCCCGTGGGAGGTGTTCCAGTCCGCCATACGACCATACCGTTCGGGCATGGCACTATGTACGCGATATC
GCTAGCGTTTGCGGGGCACAGCAATATGCAGGCATGCAAGCTTGGCGTAATCATGGTCATAGCTGTTTCCTGTGTGAAATT
GTTATCCGCTCACAATTCCACACAACATACGAGCCGGAAGCATAAAGTGTAAAGCCTGGGGTGCCTAATGAGTGAGCTAAC
TCACATTAATTGCGTTGCGCTCACTGCCCGCTTTCCAGTCGGGAAACCTGTCGTGCCAGCTGCATTAATGAATCGGCCAAC
GCGCGGGGAGAGGCGGTTTGCGTATTGGGCGCTCTTCCGCTTCCTCGCTCACTGACTCGCTGCGCTCGGTCGTTCGGCT
GCGGCGAGCGGTATCAGCTCACTCAAAGGCGGTAATACGGTTATCCACAGAATCAGGGGATAACGCAGGAAAGAACATGT
GAGCAAAAGGCCAGCAAAAGGCCAGGAACCGTAAAAAGGCCGCGTTGCTGGCGTTTTTCCATAGGCTCCGCCCCCCTGA
CGAGCATCACAAAAATCGACGCTCAAGTCAGAGGTGGCGAAACCCGACAGGACTATAAAGATACCAGGCGTTTCCCCCTG
GAAGCTCCCTCGTGCGCTCTCCTGTTCCGACCCTGCCGCTTACCGGATACCTGTCCGCCTTTCTCCCTTCGGGAAGCGTG
GCGCTTTCTCATAGCTCACGCTGTAGGTATCTCAGTTCGGTGTAGGTCGTTCGCTCCAAGCTGGGCTGTGTGCACGAACC
CCCCGTTCAGCCCGACCGCTGCGCCTTATCCGGTAACTATCGTCTTGAGTCCAACCCGGTAAGACACGACTTATCGCCAC
TGGCAGCAGCCACTGGTAACAGGATTAGCAGAGCGAGGTATGTAGGCGGTGCTACAGAGTTCTTGAAGTGGTGGCCTAAC
TACGGCTACACTAGAAGAACAGTATTTGGTATCTGCGCTCTGCTGAAGCCAGTTACCTTCGGAAAAAGAGTTGGTAGCTCT
TGATCCGGCAAACAAACCACCGCTGGTAGCGGTGGTTTTTTTGTTTGCAAGCAGCAGATTACGCGCAGAAAAAAAGGATCT
CAAGAAGATCCTTTGATCTTTTCTACGGGGTCTGACGCTCAGTGGAACGAAAACTCACGTTAAGGGATTTTGGTCATGAGA
TTATCAAAAAGGATCTTCACCTAGATCCTTTTAAATTAAAAATGAAGTTTTAAATCAATCTAAAGTATATATGAGTAAACTTGG
TCTGACAGTTACCAATGCTTAATCAGTGAGGCACCTATCTCAGCGATCTGTCTATTTCGTTCATCCATAGTTGCCTGACTCC
CCGTCGTGTAGATAACTACGATACGGGAGGGCTTACCATCTGGCCCCAGTGCTGCAATGATACCGCGAGACCCACGCTCA
CCGGCTCCAGATTTATCAGCAATAAACCAGCCAGCCGGAAGGGCCGAGCGCAGAAGTGGTCCTGCAACTTTATCCGCCTC
CATCCAGTCTATTAATTGTTGCCGGGAAGCTAGAGTAAGTAGTTCGCCAGTTAATAGTTTGCGCAACGTTGTTGCCATTGCT
ACAGGCATCGTGGTGTCACGCTCGTCGTTTGGTATGGCTTCATTCAGCTCCGGTTCCCAACGATCAAGGCGAGTTACATG
ATCCCCCATGTTGTGCAAAAAAGCGGTTAGCTCCTTCGGTCCTCCGATCGTTGTCAGAAGTAAGTTGGCCGCAGTGTTATC
ACTCATGGTTATGGCAGCACTGCATAATTCTCTTACTGTCATGCCATCCGTAAGATGCTTTTCTGTGACTGGTGAGTACTCA
ACCAAGTCATTCTGAGAATAGTGTATGCGGCGACCGAGTTGCTCTTGCCCGGCGTCAATACGGGATAATACCGCGCCACA
TAGCAGAACTTTAAAAGTGCTCATCATTGGAAAACGTTCTTCGGGGCGAAAACTCTCAAGGATCTTACCGCTGTTGAGATC
CAGTTCGATGTAACCCACTCGTGCACCCAACTGATCTTCAGCATCTTTTACTTTCACCAGCGTTTCTGGGTGAGCAAAAACA
GGAAGGCAAAATGCCGCAAAAAAGGGAATAAGGGCGACACGGAAATGTTGAATACTCATACTCTTCCTTTTTCAATATTATT
GAAGCATTTATCAGGGTTATTGTCTCATGAGCGGATACATATTTGAATGTATTTAGAAAAATAAACAAATAGGGGTTCCGCG
CACATTTCCCCGAAAAGTGCCACCTGACGTCTAAGAAACCATTATTATCATGACATTAACCTATAAAAATAGGCGTATCACG
AGGCCCTTTCG

LacO

CCTCTGACACATGCAGCTCCCGGACCTCTAGCCCTGCCCAGCGGAGAAGAGGCAACGACCCTCTGACAAGCAGCCCCGG
CAGAAGCAGCAGAAGAACCGACGCCCTGACCAGCTCCCCTGGCAGAGATCTGCCCCCATTCGAGGATGAGAGCGAGGGC
CTGCTGGGCACAGAGGGACCTCTGGAAGAAGAAGAGGACGGCGAGGAACTGATCGGCGACGGCATGGAACGGGACTAC
AGAGCCATCCCCGAGCTGGATGCCTATGAGGCCGAAGGCCTGGCCCTGGACGACGAGGATGTGGAAGAACTGACCGCC
AGCCAGAGAGAGGCCGCCGAGAGAGCTATGCGGCAGAGAGACAGAGAGGCTGGCAGAGGCCTGGGCAGAATGCGGAG
AGGCCTGCTGTACGACAGCGACGAGGAAGATGAGGAAAGACCCGCCAGAAAGCGGAGACAGGTGGAAAGAGCCACCGA
GGACGGGGAAGAGGATGAAGAGATGATCGAGAGCATCGAGAACCTGGAAGATCTGAAGGGCCACAGCGTGCGCGAGTG
GGTGTCAATGGCTGGCCCCAGACTGGAAATCCACCACCGGTTCAAGAACTTTCTGCGGACCCACGTGGACAGCCACGGC
CACAACGTGTTCAAAGAACGGATCAGCGACATGTGCAAAGAGAACCGCGAGAGCCTGGTCGTGAACTACGAGGATCTGG
CCGCCAGAGAACACGTGCTGGCCTACTTTCTGCnCTGAGGCCCCTGCCGAGCTGCTCCAGATCTTTGATGAGGCCGCCCT
GGAAGTGGTGCTGGCCATGTACCCTAAGTACGACCGGATCACCAACCACATCCACGTGCGGATCAGCCATCTGCCCCTG
GTGGAAGAACTGCGGAGCCTGAGACAGCTGCACCTGAACCAGCTGATCCGGACCTCCGGCGTCGTGACCTCTTGTACAG
GCGTGCTGCCCCAGCTGTCTATGGTCAAGTACAACTGCAACAAGTGCAACTTCGTGCTGGGACCCTTCTGCCAGAGCCAG
AACCAGGAAGTGAAGCCCGGCAGCTGTCCCGAGTGTCAGAGCGCCGGACCCTTCGAAGTGAACATGGAAGAGACAATCT
ACCAGAACTACCAGCGCATCCGCATAATACGACTCACTATAGGCATATGCGGTGTGAAATACCGCACAGATGCGTAAGGA
GAAAATACCGCATCAGGCGCCATTCGCCATTCAGGCTGCGCAACTGTTGGGAAGGGCGATCGGTGCGGGCCTCTTCGCT
ATTACGCCAGCTGGCGAAAGGGGGATGTGCTGCAAGGCGATTAAGTTGGGTAACGCCAGGGTTTTCCCAGTCACGACGTT

GTAAAACGACGGCCAGTGaattcCTCGATTTTTTTATGTTTAGTTTCGCGGACGACGGTTTCGAGGTGGCGGTCTGGACCAC
GCCGGAGAGCGTCGAAGCGGAGGCGGTGTTCGCCGAGATCGGGAGCTCTCACACCTACAAGGGATGTACATCAATTGTG
AGCGGATAACAATTGTTAGGGAGGAATTGTGAGCGGATAACAATTTGGAGTTGATAATTGTGAGCGGATAACAATTGGCTT
CAACGTAATTGTGAGCGGATAACAATTTCCGTACGAATGTGCCGAACTTATGGTACCGCCGAGCGCGACGGTGTGCCCGC
CTTCCTGGAGACCTCCGCGCTCCGCAACCTCCACTTCTAAAAGCGGCTCGGCTTCAACTAAACATAAAAAtACGAGGTGCC
CGAAGGACCGCGCACCTGGTGCATGACCCGCAAGCCCGGTGCCTGACGCTCGCCACACGACCCGCAGCGCCCGACCGA
AAGGAGCGCACGAcccgggGATCCGGGTCGACCGCCACCATGAGCGGCTTTGACGACCCCGGCATCTTCTACAGCGACAG
CTTCGGCGGAGATGCCCAGGCCGATGAAGGCCAGGCCAGAAAGTCTCAGCTCCAGCGGCGGTTCAAAGAGTTCCTGCGG
CAGTACAGAGTGGGCACCGACAGAACCGGCTTCACCTTCAAGTACCGGGACGAGCTGAAGCGGCACTACAACCTGGGCG
AGTACTGGATCGAGGTGGAAATGGAAGATCTGGCCAGCTTCGACGAGGACCTGGCCGACTACCTGTACAAGCAGCCCGC
CGAGCATCTCCAGCTGCTGGAAGAGGCCGCTAAAGAGGTGGCCGACGAAGTGACCAGACCCAGGCCTTCTGGCGAGGAA
GTGCTCCAGGACATCCAAGTGATGCTGAAGTCCGACGCCAGCCCCAGCAGCATCAGATCCCTGAAGTCTGACATGATGAG
CCACCTCGTGAAGATCCCCGGAATCATCATTGCCGCCTCTGCCGTGCGGGCCAAGGCCACCAGAATCAGCATCCAGTGC
AGAAGCTGCCGGAACACCCTGACCAATATCGCCATGCGCCCTGGCCTGGAAGGCTACGCCCTGCCCAGAAAGTGCAACA
CCGACCAGGCTGGCAGACCCAAGTGCCCCCTGGACCCCTACTTCATCATGCCCGACAAGTGCAAATGCGTGGACTTCCA
GACCCTGAAACTCCAGGAACTGCCCGACGCCGTGCCTCACGGCGAAATGCCTAGACACATGCAGCTGTACTGCGACAGA
TACCTGTGCGACAAGGTGGTGCCCGGCAACAGAGTGACCATCATGGGCATCTACAGCATCAAGAAGTTCGGCCTGACCAC
CAGCCGGGGCAGAGATAGAGTGGGCGTGGGCATCCGGTCCAGCTACATCAGAGTGCTGGGCATCCAGGTGGACACCGA
TGGCAGCGGCAGATCCTTTGCTGGGGCCGTGAGCCCCCAGGAAGAGGAAGAGTTTAGACGGCTGGCCGCCCTGCCTAAC
GTGTACGAAGTGATCAGCAAGTCTATCGCCCCCAGCATCTTCGGCGGCACCGATATGAAGAAGGCCATTGCCTGCCTGCT
GTTCGGCGGCTCCAGAAAGAGACTGCCTGACGGCCTGACAAGACGGGGCGACATCAACCTGCTGATGCTGGGCGATCCT
GGCACCGCCAAATCACAGCTGCTGAAGTTCGTGGAAAAGTGCAGCCCCATCGGCGTGTACACCAGCGGCAAGGGATCTT
CTGCCGCCGGACTGACAGCCAGCGTGATGAGAGATCCCAGCAGCCGGAATTTCATCATGGAAGGCGGCGCTATGGTGCT
GGCCGATGGCGGAGTCGTGTGCATCGACGAGTTCGACAAGATGCGCGAGGACGACCGGGTGGCCATTCACGAGGCTAT
GGAACAGCAGACCATCTCTATCGCCAAGGCCGGAATCACCACCACCCTGAACAGCCGGTGTAGCGTGCTGGCTGCCGCC
AATAGCGTGTTCGGCAGATGGGACGAGACAAAGGGCGAGGACAACATCGACTTCATGCCCACCATCCTGAGCCGCTTCG
ACATGATCTTCATCGTGAAGGACGAGCACAACGAGGAACGGGACGTGATGCTGGCCAAGCACGTGATCACCCTGCACGT
GTCCGCCCTGACACAGACACAGGCCGTGGAAGGCGAGATCGACCTGGCCAAACTGAAGAAGTTTATCGCCTACTGTCGC
GTGAAGTGCGGCCCCAGACTGTCTGCCGAGGCCGCCGAGAAGCTGAAGAACCGGTACATCATCATGCGGAGCGGAGCC
CGGCAGCACGAGAGAGACAGCGATCGGAGAAGCAGCATCCCCATCACCGTGCGGCAGCTGGAAGCCATCGTGCGGATT
GCCGAAGCCCTGAGCAAGATGAAGCTCCAGCCCTTCGCCACCGAGGCCGACGTGGAAGAAGCTCTGAGACTGTTCCAGG
TGTCAACCCTGGACGCCGCCCTGAGCGGAACACTGTCTGGCGTGGAAGGATTCACCAGCCAGGAAGATCAGGAAATGCT
GAGCAGAATCGAGAAACAGCTGAAGAGAAGATTCGCCATCGGAAGCCAGGTGTCCGAGCACAGCATCATCAAGGACTTCA
CCAAGCAGAAGTACCCCGAGCACGCCATCCACAAGGTGCTCCAGCTGATGCTGAGAAGAGGCGAGATCCAGCACCGGAT
GCAGCGGAAGGTGCTGTACCGGCTGAAATGAGGCGCCGGAATCTTTGCAGGTGCTTACGCTTACTACCTAAACTACAATG
GTGTTGTCGCTACTAGTGCCGCTTCTTCATCCACTGCATCTGGTGCTTCCGCTTCCGTTACCGGTTCTAAGAAACTTCAGA
AACTTCAGAAACCAGTGCTGCCCCTAAGACATACACTACTGCCACTGTTACTCAATGTGATGACAATGGTTGTAACGTCAA
GATAATCACCTCTCAAATACCTGAAGCTACTTCAACCGTCACCGCAACTAGTGCTTCTCCAAAGTCATACACTACTGTCACT
TCTGAGGGTTCTAAAGCAACCTCATTGAGTTCTCACTGCCCGCTTTCCAGTCGGGAAACCTGTCGTGCCAGCTGCATTAAT
GAATCGGCCAACGCGCGGGGAGAGGCGGTTTGCGTATTGGGCGCTCTTCCGCTTCCTCGCTCACTGACTCGCTGCGCTC
GGTCGTTCGGCTGCGGCGAGCGGTATCAGCTCACTCAAAGGCGGTAATACGGTTATCCACAGAATCAGGGGATAACGCA
GGACAGAACATGTGAGCAGAAGGCCAGCACGAGGCCAGGAGCCGTAAGAAGGCCGCGTTGCTGGCGTTTTTCCATAGGC
TCCGCCCCCTCGCGAGCTGAGGACTTAAGTGCTGAGGTCGCGAGCATTCAGAAGAGCCATTCAAGGGATCGATGTGGTC
GCACGACTACGCATCCCTCTGGGCCTTACATAGCCGGATACAGTGACTTATCGATACGCGTGATGCCTTGCCTGCAGAGC
CGCGTGTTAACCTCGAGGCGGCCGCCATGGTGACAGGACTTGCATAGCTGCGTGCGGGGGAAGGAACTCTTGCGTGTGA
GTATGTAGACCCCTGTACTACGGATGCGGGCAGAAGATGTGGGCAGAGACACCCGCGTCAAGTTCTCGACCTTCCCGTG
GGAGGTGTTCCAGTCCGCCATACGACCATACCGTTCGGGCATGGCACTATGTACGCGATATCGCTAGCGTTTGCGGGGCA
CAGCAATATGCAGGCATGCAAGCTTGGCGTAATCATGGTCATAGCTGTTTCCTGTGTGAAATTGTTATCCGCTCACAATTCC
ACACAACATACGAGCCGGAAGCATAAAGTGTAAAGCCTGGGGTGCCTAATGAGTGAGCTAACTCACATTAATTGCGTTGCG
CTCACTGCCCGCTTTCCAGTCGGGAAACCTGTCGTGCCAGCTGCATTAATGAATCGGCCAACGCGCGGGGAGAGGCGGT

TTGCGTATTGGGCGCTCTTCCGCTTCCTCGCTCACTGACTCGCTGCGCTCGGTCGTTCGGCTGCGGCGAGCGGTATCAG
CTCACTCAAAGGCGGTAATACGGTTATCCACAGAATCAGGGGATAACGCAGGAAAGAACATGTGAGCAAAAGGCCAGCAA
AAGGCCAGGAACCGTAAAAAGGCCGCGTTGCTGGCGTTTTTCCATAGGCTCCGCCCCCCTGACGAGCATCACAAAAATCG
ACGCTCAAGTCAGAGGTGGCGAAACCCGACAGGACTATAAAGATACCAGGCGTTTCCCCCTGGAAGCTCCCTCGTGCGCT
CTCCTGTTCCGACCCTGCCGCTTACCGGATACCTGTCCGCCTTTCTCCCTTCGGGAAGCGTGGCGCTTTCTCATAGCTCA
CGCTGTAGGTATCTCAGTTCGGTGTAGGTCGTTCGCTCCAAGCTGGGCTGTGTGCACGAACCCCCCGTTCAGCCCGACC
GCTGCGCCTTATCCGGTAACTATCGTCTTGAGTCCAACCCGGTAAGACACGACTTATCGCCACTGGCAGCAGCCACTGGT
AACAGGATTAGCAGAGCGAGGTATGTAGGCGGTGCTACAGAGTTCTTGAAGTGGTGGCCTAACTACGGCTACACTAGAAG
AACAGTATTTGGTATCTGCGCTCTGCTGAAGCCAGTTACCTTCGGAAAAAGAGTTGGTAGCTCTTGATCCGGCAAACAAAC
CACCGCTGGTAGCGGTGGTTTTTTTGTTTGCAAGCAGCAGATTACGCGCAGAAAAAAAGGATCTCAAGAAGATCCTTTGAT
CTTTTCTACGGGGTCTGACGCTCAGTGGAACGAAAACTCACGTTAAGGGATTTTGGTCATGAGATTATCAAAAAGGATCTT
CACCTAGATCCTTTTAAATTAAAAATGAAGTTTTAAATCAATCTAAAGTATATATGAGTAAACTTGGTCTGACAGTTACCAATG
CTTAATCAGTGAGGCACCTATCTCAGCGATCTGTCTATTTCGTTCATCCATAGTTGCCTGACTCCCCGTCGTGTAGATAACT
ACGATACGGGAGGGCTTACCATCTGGCCCCAGTGCTGCAATGATACCGCGAGACCCACGCTCACCGGCTCCAGATTTATC
AGCAATAAACCAGCCAGCCGGAAGGGCCGAGCGCAGAAGTGGTCCTGCAACTTTATCCGCCTCCATCCAGTCTATTAATT
GTTGCCGGGAAGCTAGAGTAAGTAGTTCGCCAGTTAATAGTTTGCGCAACGTTGTTGCCATTGCTACAGGCATCGTGGTGT
CACGCTCGTCGTTTGGTATGGCTTCATTCAGCTCCGGTTCCCAACGATCAAGGCGAGTTACATGATCCCCCATGTTGTGCA
AAAAAGCGGTTAGCTCCTTCGGTCCTCCGATCGTTGTCAGAAGTAAGTTGGCCGCAGTGTTATCACTCATGGTTATGGCAG
CACTGCATAATTCTCTTACTGTCATGCCATCCGTAAGATGCTTTTCTGTGACTGGTGAGTACTCAACCAAGTCATTCTGAGA
ATAGTGTATGCGGCGACCGAGTTGCTCTTGCCCGGCGTCAATACGGGATAATACCGCGCCACATAGCAGAACTTTAAAAG
TGCTCATCATTGGAAAACGTTCTTCGGGGCGAAAACTCTCAAGGATCTTACCGCTGTTGAGATCCAGTTCGATGTAACCCA
CTCGTGCACCCAACTGATCTTCAGCATCTTTTACTTTCACCAGCGTTTCTGGGTGAGCAAAAACAGGAAGGCAAAATGCCG
CAAAAAAGGGAATAAGGGCGACACGGAAATGTTGAATACTCATACTCTTCCTTTTTCAATATTATTGAAGCATTTATCAGGG
TTATTGTCTCATGAGCGGATACATATTTGAATGTATTTAGAAAAATAAACAAATAGGGGTTCCGCGCACATTTCCCCGAAAA
GTGCCACCTGACGTCTAAGAAACCATTATTATCATGACATTAACCTATAAAAATAGGCGTATCACGAGGCCCTTTCG

Control (Figure EV1A and B)

CCTCTGACACATGCAGCTCCCGGACCTCTAGCCCTGCCCAGCGGAGAAGAGGCAACGACCCTCTGACAAGCAGCCCCGG
CAGAAGCAGCAGAAGAACCGACGCCCTGACCAGCTCCCCTGGCAGAGATCTGCCCCCATTCGAGGATGAGAGCGAGGGC
CTGCTGGGCACAGAGGGACCTCTGGAAGAAGAAGAGGACGGCGAGGAACTGATCGGCGACGGCATGGAACGGGACTAC
AGAGCCATCCCCGAGCTGGATGCCTATGAGGCCGAAGGCCTGGCCCTGGACGACGAGGATGTGGAAGAACTGACCGCC
AGCCAGAGAGAGGCCGCCGAGAGAGCTATGCGGCAGAGAGACAGAGAGGCTGGCAGAGGCCTGGGCAGAATGCGGAG
AGGCCTGCTGTACGACAGCGACGAGGAAGATGAGGAAAGACCCGCCAGAAAGCGGAGACAGGTGGAAAGAGCCACCGA
GGACGGGGAAGAGGATGAAGAGATGATCGAGAGCATCGAGAACCTGGAAGATCTGAAGGGCCACAGCGTGCGCGAGTG
GGTGTCAATGGCTGGCCCCAGACTGGAAATCCACCACCGGTTCAAGAACTTTCTGCGGACCCACGTGGACAGCCACGGC
CACAACGTGTTCAAAGAACGGATCAGCGACATGTGCAAAGAGAACCGCGAGAGCCTGGTCGTGAACTACGAGGATCTGG
CCGCCAGAGAACACGTGCTGGCCTACTTTCTGCCTGAGGCCCCTGCCGAGCTGCTCCAGATCTTTGATGAGGCCGCCCT
GGAAGTGGTGCTGGCCATGTACCCTAAGTACGACCGGATCACCAACCACATCCACGTGCGGATCAGCCATCTGCCCCTG
GTGGAAGAACTGCGGAGCCTGAGACAGCTGCACCTGAACCAGCTGATCCGGACCTCCGGCGTCGTGACCTCTTGTACAG
GCGTGCTGCCCCAGCTGTCTATGGTCAAGTACAACTGCAACAAGTGCAACTTCGTGCTGGGACCCTTCTGCCAGAGCCAG
AACCAGGAAGTGAAGCCCGGCAGCTGTCCCGAGTGTCAGAGCGCCGGACCCTTCGAAGTGAACATGGAAGAGACAATCT
ACCAGAACTACCAGCGCATCCGCATAATACGACTCACTATAGGCATATGCGGTGTGAAATACCGCACAGATGCGTAAGGA
GAAAATACCGCATCAGGCGCCATTCGCCATTCAGGCTGCGCAACTGTTGGGAAGGGCGATCGGTGCGGGCCTCTTCGCT
ATTACGCCAGCTGGCGAAAGGGGGATGTGCTGCAAGGCGATTAAGTTGGGTAACGCCAGGGTTTTCCCAGTCACGACGTT
GTAAAACGACGGCCAGTGAATTCGAGCTCGGTACCCGGGGATCCTCTAGAGTCGACGAAGACCACCAGCTAGTACAACCA
CATACTTTATGGAGAAATTTCAAAACGCAAAGAAGAACGAAGATAAACAAATTGCCAAGTTTGAAAGCATGATGAATGCAAG
AGTACATACGTTCAGTACCGATGAGAAGAAATATGTGCCGATAATCACAAACGAATTAGAAAGCTTTTCAAATCTTTGGGTT
AAAAAGAGGTACATACCTGAAGATGACTTAAAACGGGCTTTGCATGAGATCAAAATCCTTCGGTTGGGCAAACTTTTTGCTA
AAATTCGCCCACCTAAATTTCAAGAGCCTGAATACGCCAACTGGGCCACCGTAGGCCTCATTAGCCACAAATCGGACATCA

AATTTACATCATCTGAGACGTGTCCCGTCGACCTGCATAGCGCTCAATTGCAACCACTGCAGAGTCATTCTGAGAATAGTG
TATGCGGCGACCGAGTTGCTCTTGCCCGGCGTCAATACGGGATAGTACCGCGCCACATAGCAGCACTTTGAAAGTGCTCA
TCATTGGAAAACGTTCTTCGGGGCGAAAACTCTCAAGGATCTTACCGCTGTTGAGATCCAGTTCGATGTAACCCACTCGTG
CACCCAACTGATCTTCAGCATCTTTTACTTTCACCAGCGTTTCTGGGTGAGCAAGAACAGGAAGGCACAATGCCGCAGACA
AGGGAATAAGGGCGACACGGAAATGTTGAATACTCATACTCTTCCTGCTTCAATAGTATTGAAGCATCTATCAGGGTTAGT
GTCTCATGAGCGGATACATATCTGAATGTATGTAGAACACTAGACACATAGGGGTTCCGCGCACATTTCCCCGAGAAGTGC
CACCTGACGTCTAAGACACCAGTATCATCATGACATTGACCTCTAGACgGATCCGGGTCGACCGCCACCATGAGCGGCTTT
GACGACCCCGGCATCTTCTACAGCGACAGCTTCGGCGGAGATGCCCAGGCCGATGAAGGCCAGGCCAGAAAGTCTCAGC
TCCAGCGGCGGTTCAAAGAGTTCCTGCGGCAGTACAGAGTGGGCACCGACAGAACCGGCTTCACCTTCAAGTACCGGGA
CGAGCTGAAGCGGCACTACAACCTGGGCGAGTACTGGATCGAGGTGGAAATGGAAGATCTGGCCAGCTTCGACGAGGAC
CTGGCCGACTACCTGTACAAGCAGCCCGCCGAGCATCTCCAGCTGCTGGAAGAGGCCGCTAAAGAGGTGGCCGACGAAG
TGACCAGACCCAGGCCTTCTGGCGAGGAAGTGCTCCAGGACATCCAAGTGATGCTGAAGTCCGACGCCAGCCCCAGCAG
CATCAGATCCCTGAAGTCTGACATGATGAGCCACCTCGTGAAGATCCCCGGAATCATCATTGCCGCCTCTGCCGTGCGGG
CCAAGGCCACCAGAATCAGCATCCAGTGCAGAAGCTGCCGGAACACCCTGACCAATATCGCCATGCGCCCTGGCCTGGA
AGGCTACGCCCTGCCCAGAAAGTGCAACACCGACCAGGCTGGCAGACCCAAGTGCCCCCTGGACCCCTACTTCATCATG
CCCGACAAGTGCAAATGCGTGGACTTCCAGACCCTGAAACTCCAGGAACTGCCCGACGCCGTGCCTCACGGCGAAATGC
CTAGACACATGCAGCTGTACTGCGACAGATACCTGTGCGACAAGGTGGTGCCCGGCAACAGAGTGACCATCATGGGCATC
TACAGCATCAAGAAGTTCGGCCTGACCACCAGCCGGGGCAGAGATAGAGTGGGCGTGGGCATCCGGTCCAGCTACATCA
GAGTGCTGGGCATCCAGGTGGACACCGATGGCAGCGGCAGATCCTTTGCTGGGGCCGTGAGCCCCCAGGAAGAGGAAG
AGTTTAGACGGCTGGCCGCCCTGCCTAACGTGTACGAAGTGATCAGCAAGTCTATCGCCCCCAGCATCTTCGGCGGCACC
GATATGAAGAAGGCCATTGCCTGCCTGCTGTTCGGCGGCTCCAGAAAGAGACTGCCTGACGGCCTGACAAGACGGGGCG
ACATCAACCTGCTGATGCTGGGCGATCCTGGCACCGCCAAATCACAGCTGCTGAAGTTCGTGGAAAAGTGCAGCCCCATC
GGCGTGTACACCAGCGGCAAGGGATCTTCTGCCGCCGGACTGACAGCCAGCGTGATGAGAGATCCCAGCAGCCGGAATT
TCATCATGGAAGGCGGCGCTATGGTGCTGGCCGATGGCGGAGTCGTGTGCATCGACGAGTTCGACAAGATGCGCGAGGA
CGACCGGGTGGCCATTCACGAGGCTATGGAACAGCAGACCATCTCTATCGCCAAGGCCGGAATCACCACCACCCTGAAC
AGCCGGTGTAGCGTGCTGGCTGCCGCCAATAGCGTGTTCGGCAGATGGGACGAGACAAAGGGCGAGGACAACATCGACT
TCATGCCCACCATCCTGAGCCGCTTCGACATGATCTTCATCGTGAAGGACGAGCACAACGAGGAACGGGACGTGATGCTG
GCCAAGCACGTGATCACCCTGCACGTGTCCGCCCTGACACAGACACAGGCCGTGGAAGGCGAGATCGACCTGGCCAAAC
TGAAGAAGTTTATCGCCTACTGTCGCGTGAAGTGCGGCCCCAGACTGTCTGCCGAGGCCGCCGAGAAGCTGAAGAACCG
GTACATCATCATGCGGAGCGGAGCCCGGCAGCACGAGAGAGACAGCGATCGGAGAAGCAGCATCCCCATCACCGTGCG
GCAGCTGGAAGCCATCGTGCGGATTGCCGAAGCCCTGAGCAAGATGAAGCTCCAGCCCTTCGCCACCGAGGCCGACGTG
GAAGAAGCTCTGAGACTGTTCCAGGTGTCAACCCTGGACGCCGCCCTGAGCGGAACACTGTCTGGCGTGGAAGGATTCA
CCAGCCAGGAAGATCAGGAAATGCTGAGCAGAATCGAGAAACAGCTGAAGAGAAGATTCGCCATCGGAAGCCAGGTGTC
CGAGCACAGCATCATCAAGGACTTCACCAAGCAGAAGTACCCCGAGCACGCCATCCACAAGGTGCTCCAGCTGATGCTGA
GAAGAGGCGAGATCCAGCACCGGATGCAGCGGAAGGTGCTGTACCGGCTGAAATGAGGCGCCGGAATCTTTGCAGGTGC
TTACGCTTACTACCTAAACTACAATGGTGTTGTCGCTACTAGTGCCGCTTCTTCATCCACTGCATCTGGTGCTTCCGCTTCC
GTTACCGGTTCTAAGAAACTTCAGAAACTTCAGAAACCAGTGCTGCCCCTAAGACATACACTACTGCCACTGTTACTCAATG
TGATGACAATGGTTGTAACGTCAAGATAATCACCTCTCAAATACCTGAAGCTACTTCAACCGTCACCGCAACTAGTGCTTCT
CCAAAGTCATACACTACTGTCACTTCTGAGGGTTCTAAAGCAACCTCATTGAGTTCTCACTGCCCGCTTTCCAGTCGGGAA
ACCTGTCGTGCCAGCTGCATTAATGAATCGGCCAACGCGCGGGGAGAGGCGGTTTGCGTATTGGGCGCTCTTCCGCTTC
CTCGCTCACTGACTCGCTGCGCTCGGTCGTTCGGCTGCGGCGAGCGGTATCAGCTCACTCAAAGGCGGTAATACGGTTAT
CCACAGAATCAGGGGATAACGCAGGACAGAACATGTGAGCAGAAGGCCAGCACGAGGCCAGGAGCCGTAAGAAGGCCG
CGTTGCTGGCGTTTTTCCATAGGCTCCGCCCCCTCGCGAGCTGAGGACTTAAGTGCTGAGGTCGCGAGCATTCAGAAGAG
CCATTCAAGGGATCGATGTGGTCGCACGACTACGCATCCCTCTGGGCCTTACATAGCCGGATACAGTGACTTATCGATAC
GCGTGTTAACCTCGAGGCGGCCGCCATGGTGACAGGACTTGCATAGCTGCGTGCGGGGGAAGGAACTCTTGCGTGTGAG
TATGTAGACCCCTGTACTACGGATGCGGGCAGAAGATGTGGGCAGAGACACCCGCGTCAAGTTCTCGACCTTCCCGTGG
GAGGTGTTCCAGTCCGCCATACGACCATACCGTTCGGGCATGGCACTATGTACGCGATATCGCTAGCGTTTGCGGGGCAC
AGCAATATGCAGGCATGCAAGCTTGGCGTAATCATGGTCATAGCTGTTTCCTGTGTGAAATTGTTATCCGCTCACAATTCCA
CACAACATACGAGCCGGAAGCATAAAGTGTAAAGCCTGGGGTGCCTAATGAGTGAGCTAACTCACATTAATTGCGTTGCG
CTCACTGCCCGCTTTCCAGTCGGGAAACCTGTCGTGCCAGCTGCATTAATGAATCGGCCAACGCGCGGGGAGAGGCGGT

TTGCGTATTGGGCGCTCTTCCGCTTCCTCGCTCACTGACTCGCTGCGCTCGGTCGTTCGGCTGCGGCGAGCGGTATCAG
CTCACTCAAAGGCGGTAATACGGTTATCCACAGAATCAGGGGATAACGCAGGAAAGAACATGTGAGCAAAAGGCCAGCAA
AAGGCCAGGAACCGTAAAAAGGCCGCGTTGCTGGCGTTTTTCCATAGGCTCCGCCCCCCTGACGAGCATCACAAAAATCG
ACGCTCAAGTCAGAGGTGGCGAAACCCGACAGGACTATAAAGATACCAGGCGTTTCCCCCTGGAAGCTCCCTCGTGCGCT
CTCCTGTTCCGACCCTGCCGCTTACCGGATACCTGTCCGCCTTTCTCCCTTCGGGAAGCGTGGCGCTTTCTCATAGCTCA
CGCTGTAGGTATCTCAGTTCGGTGTAGGTCGTTCGCTCCAAGCTGGGCTGTGTGCACGAACCCCCCGTTCAGCCCGACC
GCTGCGCCTTATCCGGTAACTATCGTCTTGAGTCCAACCCGGTAAGACACGACTTATCGCCACTGGCAGCAGCCACTGGT
AACAGGATTAGCAGAGCGAGGTATGTAGGCGGTGCTACAGAGTTCTTGAAGTGGTGGCCTAACTACGGCTACACTAGAAG
AACAGTATTTGGTATCTGCGCTCTGCTGAAGCCAGTTACCTTCGGAAAAAGAGTTGGTAGCTCTTGATCCGGCAAACAAAC
CACCGCTGGTAGCGGTGGTTTTTTTGTTTGCAAGCAGCAGATTACGCGCAGAAAAAAAGGATCTCAAGAAGATCCTTTGAT
CTTTTCTACGGGGTCTGACGCTCAGTGGAACGAAAACTCACGTTAAGGGATTTTGGTCATGAGATTATCAAAAAGGATCTT
CACCTAGATCCTTTTAAATTAAAAATGAAGTTTTAAATCAATCTAAAGTATATATGAGTAAACTTGGTCTGACAGTTACCAATG
CTTAATCAGTGAGGCACCTATCTCAGCGATCTGTCTATTTCGTTCATCCATAGTTGCCTGACTCCCCGTCGTGTAGATAACT
ACGATACGGGAGGGCTTACCATCTGGCCCCAGTGCTGCAATGATACCGCGAGACCCACGCTCACCGGCTCCAGATTTATC
AGCAATAAACCAGCCAGCCGGAAGGGCCGAGCGCAGAAGTGGTCCTGCAACTTTATCCGCCTCCATCCAGTCTATTAATT
GTTGCCGGGAAGCTAGAGTAAGTAGTTCGCCAGTTAATAGTTTGCGCAACGTTGTTGCCATTGCTACAGGCATCGTGGTGT
CACGCTCGTCGTTTGGTATGGCTTCATTCAGCTCCGGTTCCCAACGATCAAGGCGAGTTACATGATCCCCCATGTTGTGCA
AAAAAGCGGTTAGCTCCTTCGGTCCTCCGATCGTTGTCAGAAGTAAGTTGGCCGCAGTGTTATCACTCATGGTTATGGCAG
CACTGCATAATTCTCTTACTGTCATGCCATCCGTAAGATGCTTTTCTGTGACTGGTGAGTACTCAACCAAGTCATTCTGAGA
ATAGTGTATGCGGCGACCGAGTTGCTCTTGCCCGGCGTCAATACGGGATAATACCGCGCCACATAGCAGAACTTTAAAAG
TGCTCATCATTGGAAAACGTTCTTCGGGGCGAAAACTCTCAAGGATCTTACCGCTGTTGAGATCCAGTTCGATGTAACCCA
CTCGTGCACCCAACTGATCTTCAGCATCTTTTACTTTCACCAGCGTTTCTGGGTGAGCAAAAACAGGAAGGCAAAATGCCG
CAAAAAAGGGAATAAGGGCGACACGGAAATGTTGAATACTCATACTCTTCCTTTTTCAATATTATTGAAGCATTTATCAGGG
TTATTGTCTCATGAGCGGATACATATTTGAATGTATTTAGAAAAATAAACAAATAGGGGTTCCGCGCACATTTCCCCGAAAA
GTGCCACCTGACGTCTAAGAAACCATTATTATCATGACATTAACCTATAAAAATAGGCGTATCACGAGGCCCTTTCG

Appendix Figure S1

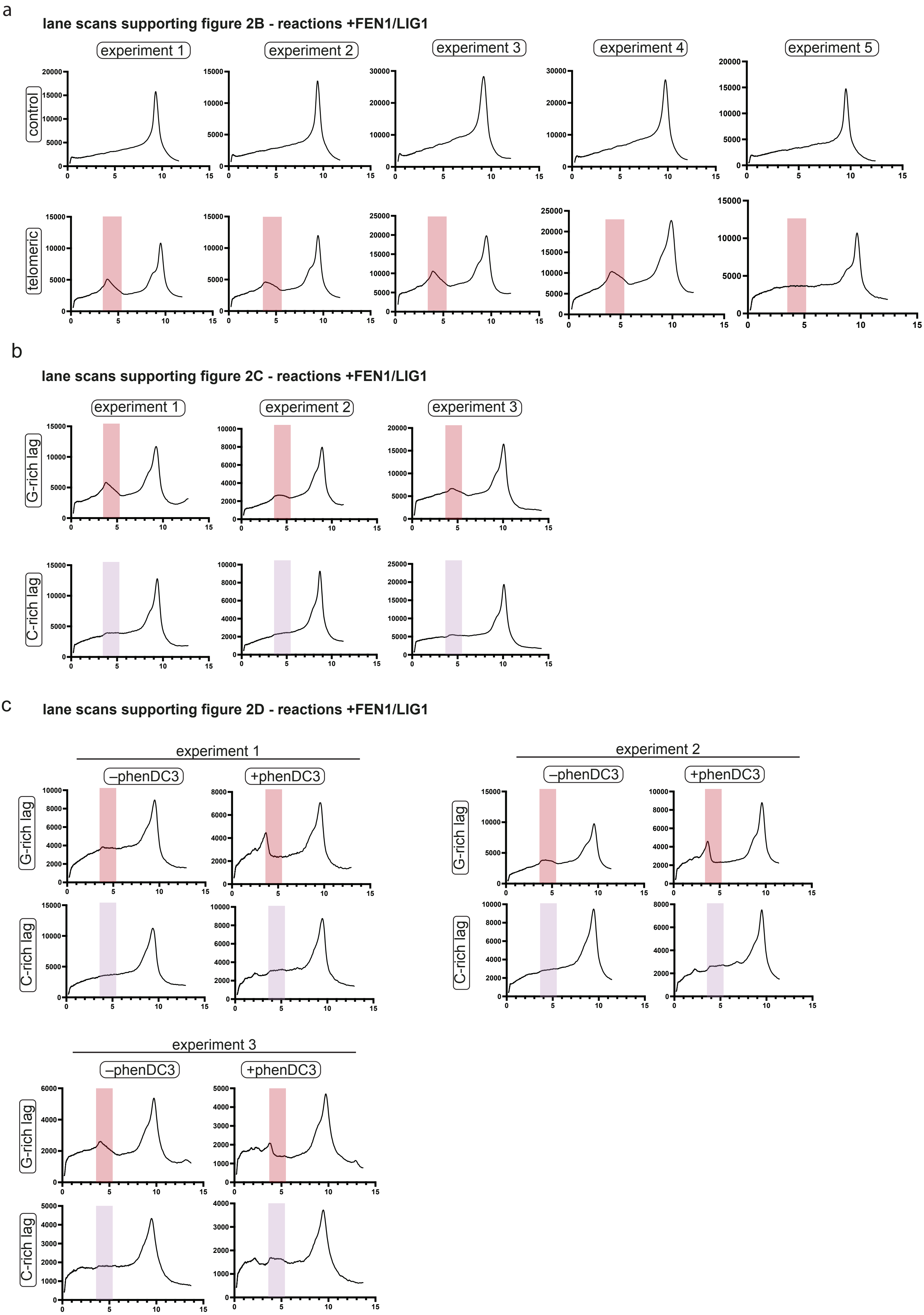

d

lane scans supporting figure 2E - reactions +FEN1/LIG1

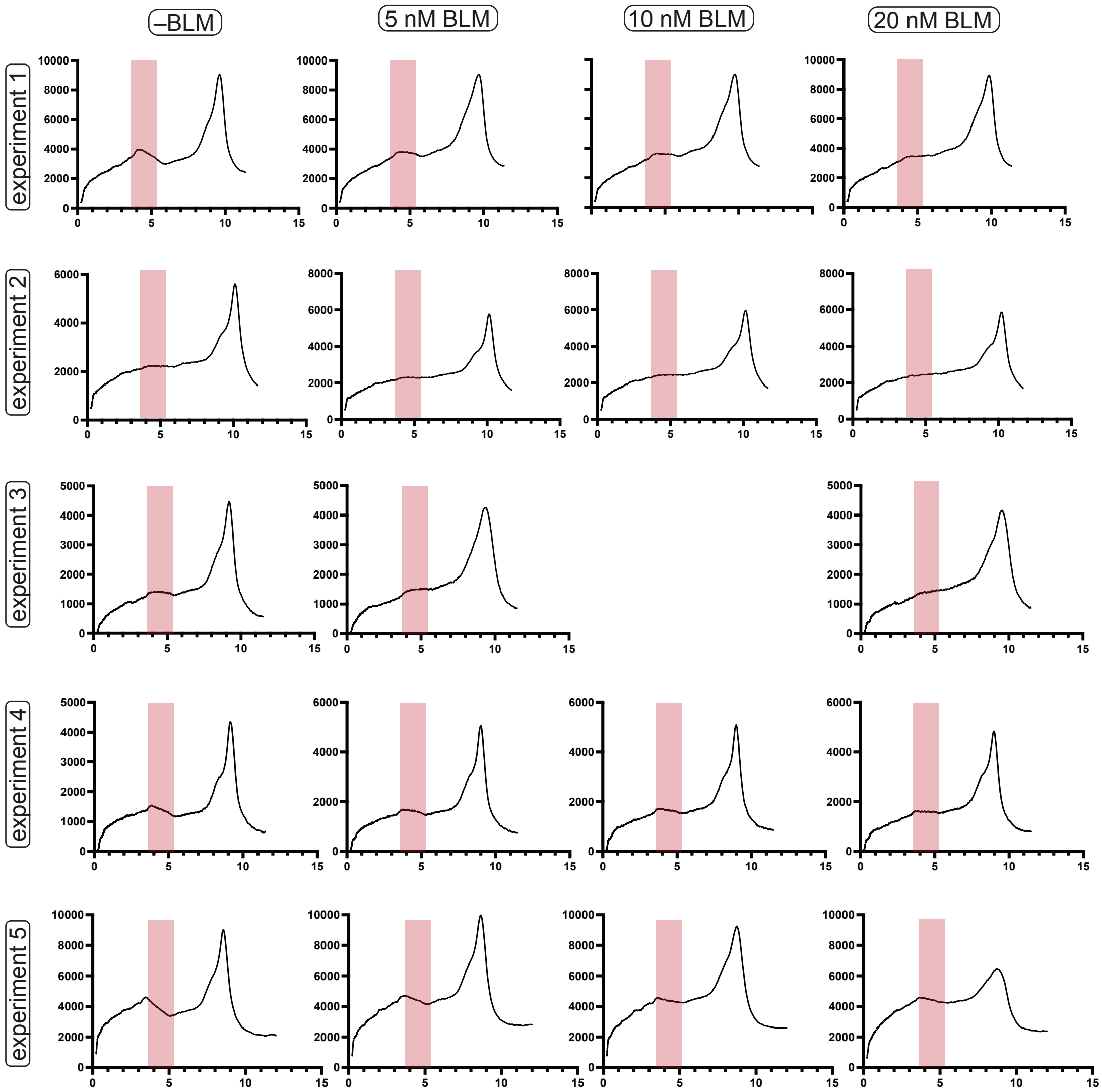

Supplement: Supplementary file 1 [file 44318_2026_844_MOESM1_ESM.pdf]
