# Supplementary material for: Inhibition of lagging strand replication by G-rich telomeric DNA and the shelterin subunit POT1
Source: EMBO J. 2026 Jul 9;45(16):5793–826. doi: 10.1038/s44318-026-00844-7 (PMC13483900; doi:10.1038/s44318-026-00844-7)
Supplement: Supplementary file 2 [file 44318_2026_844_MOESM2_ESM.pdf]

THE EMBO JOURNAL

Inhibition of lagging strand replication by G-rich telomeric DNA and the shelterin subunit POT1

Ciara Leonard-Booker, Giulia Mazzucco, Charlotte Fisher, Karim Hussain, Tom Deegan, Ylli Doksani, and Max Douglas

Corresponding author(s): Max Douglas (max.douglas@icr.ac.uk)

**Review Timeline:**		
	Submission Date:	16th Sep 25
	Editorial Decision:	27th Oct 25
	Revision Received:	1st May 26
	Editorial Decision:	26th May 26
	Revision Received:	15th Jun 26
	Accepted:	16th Jun 26

Editor: Hartmut Vodermaier

Transaction Report:

(Note: With the exception of the correction of typographical or spelling errors that could be a source of ambiguity, letters and reports are not edited. Depending on transfer agreements, referee reports obtained elsewhere may or may not be included in this compilation. Referee reports are anonymous unless the Referee chooses to sign their reports.)

Dr. Max E Douglas
The Institute of Cancer Research
Cell and Molecular Biology
237 Fulham Road
London SW3 6JB
United Kingdom

27th Oct 2025

Re: EMBOJ-2025-122482
Inhibition of lagging strand replication by G-rich telomeric DNA and the shelterin subunit POT1

Dear Max,

Thank you for submitting your manuscript on recombinant reconstitution of human telomere replication for our consideration. I sent it to three expert referees, who have now returned the reports that are copied below. As you will see, all referees agree on the importance of the subject and the value of your system; but they also raise a number of substantive issues that would need to clarified prior to publication. Recurrent comments concern insufficient experimental explanations, uncertainties about stoichiometries and protein complex formation, but also the need for more quantitative assessments. In addition, each of the referees bring up suggestions for extensions that might provide deeper insights, such as consideration of nucleosome, additional helicases, or longer assessment of larger telomeric inserts, incorporation of some of which may help to further strengthen the study.

In this situation, I would like to give you an opportunity to address the referees' points by way of a revised manuscript, which we would be happy to consider further for EMBO Journal publication. However, I should remind you that our single-major-revision-round policy makes it important to comprehensively respond to each referee point at the time of resubmission; I would therefore encourage you to contact me with a preliminary point-by-point response and revision plan already during the early stages of your revision work, in order to clarify if and how key issues raised in the reports may be solved. We would also be open to extension of the regular three-months revision period if needed; our 'scooping protection' (meaning that competing work appearing elsewhere in the meantime will not affect our considerations of your study) would of course remain valid also throughout such an extension.

Further information on preparing, formatting and uploading a revised manuscript can be found below and in our Guide to Authors. Thank you again for the opportunity to consider this work for The EMBO Journal, and I look forward to hearing from you soon.

With kind regards,

Hartmut

Hartmut Vodermaier, PhD
Senior Editor, The EMBO Journal
h.vodermaier@embojournal.org

*** PLEASE NOTE: All revised manuscript are subject to initial checks for completeness and adherence to our formatting guidelines. Revisions may be returned to the authors and delayed in their editorial re-evaluation if they fail to comply to the following requirements (see also our Guide to Authors for further information):

1) Every manuscript requires a Data Availability section (even if only stating that no deposited datasets are included). Primary datasets or computer code produced in the current study have to be deposited in appropriate public repositories prior to resubmission, and reviewer access details provided in case that public access is not yet allowed. Further information: embopress.org/page/journal/14602075/authorguide#dataavailability

2) Each figure legend must specify
- size of the scale bars that are mandatory for all micrograph panels
- the statistical test used to generate error bars and P-values
- the type error bars (e.g., S.E.M., S.D.)
- the number (n) and nature (biological or technical replicate) of independent experiments underlying each data point
- Figures may not include error bars for experiments with n<3; scatter plots showing individual data points should be used

instead.

3) Revised manuscript text (including main tables, and figure legends for main and EV figures) has to be submitted as editable text file (e.g., .docx format). We encourage highlighting of changes (e.g., via text color) for the referees' reference.

4) Each main and each Expanded View (EV) figure should be uploaded as individual production-quality files (preferably in .eps, .tif, .jpg formats). For suggestions on figure preparation/layout, please refer to our Figure Preparation Guidelines: http://bit.ly/EMBOPressFigurePreparationGuideline

5) Point-by-point response letters should include the original referee comments in full together with your detailed responses to them (and to specific editor requests if applicable), and also be uploaded as editable (e.g., .docx) text files.

6) Please complete our Author Checklist, and make sure that information entered into the checklist is also reflected in the manuscript; the checklist will be available to readers as part of the Review Process File. A download link is found at the top of our Guide to Authors: embopress.org/page/journal/14602075/authorguide

7) All authors listed as (co-)corresponding need to deposit, in their respective author profiles in our submission system, a unique ORCiD identifier linked to their name. Please see our Guide to Authors for detailed instructions.

8) Please note that supplementary information at EMBO Press has been superseded by the 'Expanded View' for inclusion of additional figures, tables, movies or datasets; with up to five EV Figures being typeset and directly accessible in the HTML version of the article. For details and guidance, please refer to: embopress.org/page/journal/14602075/authorguide#expandedview

9) To facilitate reproducibilty and cross-laboratory adoption of methodologies, please structure the Materials & Methods section as outlined in our guide to authors, including a completed Reagents and Tools Table that can be downloaded from our author guidelines as well (https://www.embopress.org/page/journal/14602075/authorguide#structuredmethods).

10) Digital image enhancement is acceptable practice, as long as it accurately represents the original data and conforms to community standards. If a figure has been subjected to significant electronic manipulation, this must be clearly noted in the figure legend and/or the 'Materials and Methods' section. The editors reserve the right to request original versions of figures and the original images that were used to assemble the figure. Finally, we generally encourage uploading of numerical as well as gel/blot image source data; for details see: embopress.org/page/journal/14602075/authorguide#sourcedata

At EMBO Press we ask authors to provide source data for the main manuscript figures. You will receive a separate email with instructions for providing source data with your revised manuscript, including how to upload and organize the files.

Further information is available in our Guide For Authors:
https://www.embopress.org/page/journal/14602075/authorguide

In the interest of ensuring the conceptual advance provided by the work, we recommend submitting a revision within 3 months (25th Jan 2026). Please discuss the revision progress ahead of this time with the editor if you require more time to complete the revisions. Use the link below to submit your revision:

Link Not Available

Referee #1:

This study represents a major accomplishment in mammalian telomere biochemistry. Chromosome end replication has been studied in parts, both in terms of studying G-strand extension by telomerase and C-strand fill-in by CST-Pol-alpha, and in terms of studying individual proteins, protein complexes, or enzymes. This study is a major leap in that regard since it reconstitutes the mammalian replisome and shelterin complexes to study telomere replication in vitro. The purity of the complexes and the quality of the replication assays are top-notch. The observations of shelterin proteins acting as additional hurdles to the replication machinery go against the notion of them helping with replication through telomeric structures, changing the way we think about shelterin in telomere replication. Overall, this study marks a significant step forward in reconstituting a fundamental process in mammalian molecular biology. It sets the stage for answering longstanding questions in chromosome end protection and replication. Below are a few points to consider before publication. They are divided into major and minor points as per the journal's request.
Major points

1. The shelterin proteins used are of high quality based on the SDS-PAGE data. However, do we know if the proteins form a complex or form a mixture of subcomplexes in addition to or instead of a full complex? This was not clear from the results or methods. (I apologize if I didn't read them carefully!) Were all six proteins co-expressed in insect cells? Only POT1 and TPP1 out of the six proteins are mentioned to be made in insect cells. In addition to clarifying these details in the methods, the information on complex formation would be helpful to include, unless it was shown in a previous publication. The shelterin context dependence of the POT1 phenotype indicates the existence of a full complex, but it is not direct evidence. This could be done with SEC, SEC-MALS, or Mass Photometry, to cite a few methods, on the purified shelterin complex. Shelterin is known to be conformationally heterogeneous, but is it also compositionally heterogeneous under the conditions of this study?
2. The authors duly note that super-stoichiometric quantities of CST are needed to counter the POT1 phenotype in their replication assays. Given that CST binds POT1 and both encompass single-stranded DNA binding activity, there are several conceivable mechanisms for how CST counters POT1. This includes CST sequestering POT1 off telomeres by binding it and masking its DNA-binding function. EMSAs or other binding/competition experiments could be used to answer what the status of POT1, CST, and the replication templates/intermediates are with respect to each other. Under conditions of the replication assays where CST is effective, it would be nice to know if it is bound to DNA, POT1, or both. And the same question for POT1, whether it is bound to CST, DNA, or both.
3. The current study is a major biochemical achievement in mammalian telomere biology. It sets the stage for building up further complexity, including the context of telomeric nucleosomes, whose structural biology is gaining traction. Although I am not suggesting inclusion of nucleosomes in this study, it seems important to put the results of this study in the context of what is known about the interaction of shelterin and nucleosomes and how the authors think including nucleosomes in their experiments could change the results or interpretation of this study (in the Discussion section).

Minor points
4. Although this is stated in the legend, it would be useful to indicate in the figure 3 gel annotation that all lanes contain shelterin, including the lane labeled "-"
5. Consider changing "Electromobility shift assay" to the more standard term Electrophoretic Mobility Shift Assay
6. Replace "is" by "are" in "The details of the affinity tagging strategy and expression system used for each factor is detailed in Supplementary table S1."

JK Nandakumar

Referee #2:

The study seeks to understand mechanisms responsible for telomeres being difficult to replication regions of the genome by reconstituting the replisome on substrates containing telomeric duplex sequences in the presence and absence of telomere shelterin proteins. The authors should be applauded for such a large undertaking in purifying numerous proteins to achieve reconstitution. The studies confirmed that G-quadruplex formation on TTAGGG templates during OK fragment processing on the lagging strand, but not on the leading strand, stall the replisome. Further studies revealed that the shelterin complex stalls the replisome on both the leading and lagging strands, dependent on the myb telomere binding domains of TRF1 and TRF2. The POT1-TPP1 complex promotes single strand gap formation on the lagging strand dependent on POT1 binding the G-rich ssDNA, which can be partly rescued by adding the CST complex. Overall, the manuscript is well written, and the biochemistry data support the conclusions. However, there are a few points that require further clarification, particularly how the biochemical reconstitution relates to the cellular context, as detailed below.
1. For Fig. 1 and subsequent figures, it would be helpful to clarify what the replication intermediates (RI) represent on the native gel in the main text. Are these incomplete products? Do they differ in leading and lagging strand replication products?
2. Fig 2. Previous studies on circularized templates reported the Pol delta pauses on the G-rich lagging strand even in the absence of G4 folding (PMID: 24038470). Consistent with this, BLM helicase addition reduced, but did not appear to completely eliminate, stalled products (Fig. 2f). Have the authors considered whether features of the G-rich sequence on the lagging strand beyond G4 folding may contribute to stalling?
3. Fig. 3. The text should include the rationale for the stoichiometric amounts of shelterin members used. Were equal molar amounts of each component added? This should also be added to the figure legend. How do the stoichiometric amounts added relate to the physiological concentrations of shelterin, which were reported to be unequal (i.e. not 1:1 ratios) (see PMID 19864690). Based on the amount of shelterin added and the number of telomeric repeats, do the authors predict that the telomeric sequences in the replication substrates will be fully bound by shelterin at the higher concentrations? Subcomplexes of shelterin have been described. Do the authors have evidence that the added proteins form the 6-member complex? Perhaps size exclusion chromatography could address the existence of complete and sub- complexes.
4. Fig. 3a. Do the hyphen marks point to the lane or represent "minus" symbols? Please clarify in the figure legend. Why does TRF2 appear larger than its molecular weight (~60 kDa)? I would have expected that TRF1 and TRF2 would migrate the same given their similar molecular weight (see also PMID 19864690). Based on the gel it does not appear that equal amounts of shelterin components are present (see previous comment).
5. Supplemental Fig 3a. For the EMSA, it would be helpful to show the wells of the gel to ensure that the bound complex migrated into the gel, rather than being retained in the well. Controls for non-telomeric DNA should be included to ensure any effects are due to shelterin binding to the telomeric sequences in the replication model.

6. Fig. 4. The conclusion that fork stalling and the induction of gaps are separate events is counterintuitive. Could fork uncoupling due to stalling lead to gaps on the lagging strand that are then bound by POT1?
7. Have the authors considered whether BLM, in addition to RTEL1, might be able to displace shelterin? Previous reports indicate that BLM can displace RAD51 from DNA (PMID: 18003860). Therefore, it may be worth testing other helicases that prevent telomere fragility (i.e. BLM, FANCJ, WRN), based on the biochemical data presented that shelterin can stall forks.

Minor comment
1. The authors have included much detail regarding protein purification. But it would be useful to include which subunits of Pol delta (3 or 4 subunits) were included. Was the shelterin complex purified from human or insect cells?

Referee #3:

Summary:
This manuscript describes how telomeric-specific components impact telomere replisome progression using an in vitro reconstituted system. The authors reconstituted the human replisome (or subcomplexes of it) using recombinant proteins and tested its progression on a telomeric forked template. Through gel-based analysis, they found ~1.5 kb of telomeric sequences had no impact on leading strand synthesis replication fork progression but the replisome is partially stalled for lagging strand synthesis when the telomeric G-rich template is used. They then tested how various shelterin complexes affect replisome progression across telomeric DNA. Through subunit-depletion, they observed the partial replisome stalling event diminished when the TRF1/2 myb domains were removed, leading to their conclusion that TRF1 or/and TRF2 binding can stall replisome progression across telomeric sequences. Finally, they discovered shelterin addition led to ~500 nt size ssDNA gaps using EM image analysis and confirmed the ssDNA presence using P1 nuclease gel studies. These gaps went away when POT1 is omitted from the shelterin complexes. Adding CST and Pola-primase to their experiments can alleviate the POT1 effect.
This work addresses multiple important questions in telomere replication and uses a powerful technique to do so. However, there are several major concerns regarding reproducibility, controls, and a lack of quantitative and statistical treatments that could strongly impact the conclusions made in this manuscript. Most interpretations and conclusions are handwaving and qualitative at best. In addition, the presentation, organization and writing of the manuscript need improvement.
Please see our comments below for details:
Major Comments:
1. Telomere replication studies have been waiting for a long time for a reconstituted system that allows scientists to carefully tease out the various mechanisms in place for the replisome to navigate the repetitive DNA landscape. Thus, we really appreciate and recognize the power of this work's approach and insights. There are several interesting observations, some consistent with cellular findings and some are not. That's expected with a cutting-edge study like this, but our enthusiasm is much dimmed when the tracked signal (stalled band) is small and relatively weaker than the unperturbed signal (full length products). Most, if not all, of the authors' analysis and conclusions are based on a faint signal. Our concern is further compounded with the current qualitative treatment and not using a quantitative statistical analysis of the stalled products (p-value across comparison, quantification of key bands, etc.). The authors should show statistical analysis (p-values should be provided) derived from quantification across three separate biochemical replicates for all experiments.
2. It is unclear if the FL signal in the gels were from unperturbed replisome products or from both leading and lagging strand synthesis. It will be helpful if the authors can offer more information to how the experimental signals were interpretated.
3. The authors have used a 1.6 kb size telomeric repeat tract as a telomeric template model and most interpretations for stalled products are perceived to stem from the telomeric insert. Have the authors tested moving the relative position of the telomeric insert and see a corresponding change in the size of the stalled band?
4. Have the authors tested a larger telomeric insert that would better recapitulate the cellular telomere length? Perhaps, a longer telomeric tract will result in more stalled products over full-length products and lead to easier quantification versus a faint band quantification.
5. Do the authors think that the replisome was stalled at the onset of arriving at the telomeric region or the stalling was evenly distributed within the telomeric repeats? We think a clarification of the stalling distribution would be an important information for the field to understand how various mitigating or non-mitigating factors work.
6. The manuscript writing needs improvements. Often, the rationale and motivation for an experiment condition were not provided before the data is described. This makes it tedious to read and especially for a reader to appreciate the result interpretation. For example, in page 9, last paragraph, the authors asked if CST can "overcome" (maybe alleviate or suppress is a better action verb?) the inhibitory effect of POT1. Then the authors described they used a reduced RPA concentration. Our question is why do they need to reduce the RPA concentration for this test? Also, what happens if they kept RPA at the same concentration as the rest of the paper? We think having someone outside the field of telomere and DNA replication to read will be extremely helpful in highlighting missing context or background information.
7. Manuscript organization: Fig 1B is skipped in the text and comes after Fig 1C is described. Also, the description of the telomeric DNA fork construct came late in page 4, 2nd paragraph. This description should be introduced earlier in that result section.
8. Figure 1d/manuscript organization: Page 4, paragraph 2 states that a pulse chase assay was used. The figure caption for figure 1 states that a chase was used. Nowhere does it state what the pulse or the chase was in a concise way for the reader. This should be made immediately clear when introducing the experiment/figure. It would be helpful if this experiment (and

others) were laid out in such a way that the reader would not need to go searching all the way to the last sentence of page 31 in the methods for essential information. Additionally, have the authors quantified the bulk replication rate after the pulse?
9. Fig. S2a lane 2 showed the products made by the addition of Pola-primase only. However, the DNA markers in the gel do not provide the appropriate range to assess the size range of primers made by Pola-primase. Based on the available marker bands, it seems the enzyme is making primers of several hundred nucleotides long. Are these just RNA-DNA primers made by Pola-primase or extended primers by other added polymerase? RNA-DNA primers made by Pola-primase are typically ~30 nt in size which would contradicts the authors interpretation of their gel signal. Also, We are not convinced that there is a significant change in the size of the OF bands (smear) after DNA polymerase delta addition. This point here also emphasizes on the need to carefully quantify these perceived changes described by the authors.
10. A major concern is the QC for recombinant shelterin complexes used in this work. The authors assigned the identity of protein bands in a SDS-PAGE in fig 3a. The largest band is assigned as TRF2 but past studies including similar analysis showed POT1 migrates slower than TRF2 and TPP1. Here, the authors assigned POT1 and TPP1 as similar migration size. We understand that depending on the tags and gel buffer system used, the migration rate of a protein can be different. Thus we ask if the authors have verified their band assignment using western blot? If yes, we think they should be provided.
11. Figure 3: As mentioned before, the faint stall bands should be quantified with replicates. This is important evidence for a reader to be convinced of the authors' conclusions. For example, in Fig 3B, the authors claim that "forks arrested on the telomere within the first five minutes remained there for at least a further 55 minutes, suggesting the replisome alone is ineffective at bypassing shelterin once a stall has taken place". The authors should quantify the stall band and provide an explanation for the increase in signal of the full-length band. A second example is Figure 3C, which the authors use to claim that "Shelterin was equally effective at stalling the replisome when either C- or G-rich telomeric DNA was the leading strand template." Again, the authors do not quantify the stall or full-length bands used to make this conclusion. Have the authors tested this conclusion with an additional data point between 25 and 45 nM shelterin?
12. It was not clear which shelterin complex was used for the EMSA shown in fig S3A. We assumed it is the largest 6 subunit shelterin complex. If yes, have the authors tested the complex stoichiometry? The reason why we are asking this is because the complex stoichiometry has a direct impact to the design of the telomeric DNA substrate used in an EMSA assay; how many TRF1 and TRF2 and POT1 binding sites to use. Along this line, a 360 bp telomeric DNA is a poor substrate to use for checking if their purified shelterin can bind specifically to telomeric DNA. The larger than necessary substrate complicates binding affinity quantification and likely also resulted in a seemingly well-shift situation. Achieving a gel-shift condition is important for proper binding analysis. Additionally, the authors should provide data to confirm that shelterin does not bind their control DNA.
13. Fig 3e: Did the authors test if adding only TRF1 or TRF2 or TIN2-TRF1-TRF2 leads to the same stalling effect, as predicted by their interpretation?
14. Fig 4a: The authors reference figure 4a on page 7-8 as if it contains data with and without shelterin. However, only data with shelterin is provided, while the data without shelterin is not presented in figures 4 or S4. This is confusing for the reader. This figure should be improved so that the quantitation is clearly visually shown in a single, unified figure, without the need for such a complicated textual explanation in the figure reference.
15. Methods: The organization of the methods section could use some clarification. In some cases, additional break points into new paragraphs will be enough to make clear which reaction is being described. In other cases, additional clarification is needed. For example, page 31 lines 5 and 6, ranges are provided for the concentration of RPA and RFC in the in vitro reactions, but it is not made clear enough in the manuscript why these are ranges or what concentration was used in each experiment.

Minor Comments:
1. Figure S1A should be included in main figure 1, as it provides essential context for the work.
2. Page 5, first paragraph: OF synthesis. We may have missed the initial definition for this acronym. If not, please provide the full name definition if this is used for the first time in the text.
3. Figure 3A should be split into three separate panels.
4. Figure 3E: What does TRF1/2 myb domain deletion mean? Both TRF1 and TRF2 deletion?
5. Nicking/gapping site annotation: The gaps used in the figures to denote gaps (2A) or nicks (4C) in the DNA are rather small, it might be helpful to make the overall picture larger or to make these sites more obvious in some other way (more colors, widened gaps, thicker line width?).

Note: This manuscript is co-reviewed with a member of a referee's laboratory as part of the mentoring process. The primary referee has independently evaluated the manuscript and agrees with the report filed.

General comments:

Many thanks for taking the time to carefully read our manuscript and for your considered and informed comments. As described in the point-by-point response below, we have added an extensive range of new experiments and rewritten large parts of the text in response to your input, which we feel has significantly strengthened the study.

Specific responses:

Referee #1:

This study represents a major accomplishment in mammalian telomere biochemistry. Chromosome end replication has been studied in parts, both in terms of studying G-strand extension by telomerase and C-strand fill-in by CST-Pol-alpha, and in terms of studying individual proteins, protein complexes, or enzymes. This study is a major leap in that regard since it reconstitutes the mammalian replisome and shelterin complexes to study telomere replication in vitro. The purity of the complexes and the quality of the replication assays are top-notch. The observations of shelterin proteins acting as additional hurdles to the replication machinery go against the notion of them helping with replication through telomeric structures, changing the way we think about shelterin in telomere replication. Overall, this study marks a significant step forward in reconstituting a fundamental process in mammalian molecular biology. It sets the stage for answering longstanding questions in chromosome end protection and replication.

We are very grateful for the feedback and are delighted with the positive comments. Many thanks, JK.

Major points

1. The shelterin proteins used are of high quality based on the SDS-PAGE data. However, do we know if the proteins form a complex or form a mixture of subcomplexes in addition to or instead of a full complex? This was not clear from the results or methods. (I apologize if I didn't read them carefully!) Were all six proteins co-expressed in insect cells?

Our apologies that this was not clear. Unless noted otherwise, all six shelterin subunits were co-expressed in insect cells, purified via a strep-tag on the TIN2 subunit and passed over a size exclusion column. We have changed the methods to clarify this point and added it into the main results text. Additionally, we have included an immunoblotting analysis (Fig EV3A) showing that the relative amounts of each subunit are consistent between two independent preparations. Please also see the response directly below.

The shelterin context dependence of the POT1 phenotype indicates the existence of a full complex, but it is not direct evidence. This could be done with SEC, SEC-MALS, or Mass Photometry, to cite a few methods, on the purified shelterin complex. Shelterin is known to be conformationally heterogeneous, but is it also compositionally heterogeneous under the conditions of this study?

We have performed mass photometry on the full complex as requested (new figure EV3D), revealing a primary peak at around 700 KDa, and several additional peaks at lower molecular weights. This profile is consistent with a previous mass photometry analysis of purified shelterin (PMID: 35881804 - please note the peak at 780 kDa in this study includes MBP- and GFP tags on several subunits) and suggests we have indeed purified a full complex, but that there are also some subcomplexes present.

2. The authors duly note that super-stoichiometric quantities of CST are needed to counter the POT1 phenotype in their replication assays. Given that CST binds POT1 and both encompass single-stranded DNA binding activity, there are several conceivable mechanisms for how CST counters POT1. This includes CST sequestering POT1 off telomeres by binding it and masking its DNA-binding function. EMSAs or other binding/competition experiments could be used to answer what the status of POT1, CST, and the replication templates/intermediates are with respect to each other.
Under conditions of the replication assays where CST is effective, it would be nice to know if it is bound to DNA, POT1, or both. And the same question for POT1, whether it is bound to CST, DNA, or both.

Many thanks for the suggestions. To examine whether CST can bind POT1 in our reactions, we performed a pulldown assay with bead-immobilised strep-TPP1/POT1 and purified CST. New figure EV7D shows that only a small fraction of TPP1/POT1 was bound to CST under the conditions of the assay, and that this was very sensitive to washing with a slightly higher salt. This finding agrees with previously published work by the de Lange lab (PMID: 38838667) that human CST and TPP1/POT1 are largely unable to bind one-another without the reported phosphoregulation. Therefore, we do not think that the DNA binding function of POT1 is likely to be masked through it forming a complex with CST here.

To examine binding of TPP1/POT1 and CST to single stranded DNA, we immobilised a single stranded 18-mer of TTAGGG repeats onto streptavidin beads, which we used as bait in pulldown assays with purified TPP1/POT1 and purified CST. New figure EV7E shows that CST could compete with TPP1/POT1 for binding to single stranded DNA. It is possible that this competition contributes to the effect of CST we have observed. In future experiments, we feel it will be important to examine the reported phosphoregulation of the CST-TPP1/POT1 interaction (PMID: 38838667), which may enable CST to more effectively counteract the replicative gaps.

3. The current study is a major biochemical achievement in mammalian telomere biology. It sets the stage for building up further complexity, including the context of telomeric nucleosomes, whose structural biology is gaining traction. Although I am not suggesting inclusion of nucleosomes in this study, it seems important to put the results of this study in the context of what is known about the interaction of shelterin and nucleosomes and how the authors think including nucleosomes in their experiments could change the results or interpretation of this study (in the Discussion section).

We are indeed very interested to see how chromatin impacts the shelterin effects and vice versa. We have now included a section in the discussion section emphasising that the

stalling effect of shelterin may be attenuated or altered by the presence of nucleosomes, especially given the ability of TRF1 and TRF2 to remodel and stabilise nucleosomes on telomeric DNA (PMID: 37624885 and PMID: 38177309). Many thanks for this suggestion and for the positive feedback.

Minor points
4. Although this is stated in the legend, it would be useful to indicate in the figure 3 gel annotation that all lanes contain shelterin, including the lane labeled "-"

Thanks for the suggestion we have changed the dash to ‘0’ throughout to avoid confusion.

5. Consider changing "Electromobility shift assay" to the more standard term Electrophoretic Mobility Shift Assay

Thanks for the suggestion. We have changed this in the revised manuscript

6. Replace "is" by "are" in "The details of the affinity tagging strategy and expression system used for each factor is detailed in Supplementary table S1."

Thanks for spotting this – it has been changed in the revised manuscript.

Referee #2:

The study seeks to understand mechanisms responsible for telomeres being difficult to replication regions of the genome by reconstituting the replisome on substrates containing telomeric duplex sequences in the presence and absence of telomere shelterin proteins. The authors should be applauded for such a large undertaking in purifying numerous proteins to achieve reconstitution.

Many thanks for the positive comments – they are much appreciated.

1. For Fig. 1 and subsequent figures, it would be helpful to clarify what the replication intermediates (RI) represent on the native gel in the main text. Are these incomplete products? Do they differ in leading and lagging strand replication products?

Indeed, these replication intermediates - also observed by a previous reconstitution of the human replisome (PMID: 35585232) - are likely to derive from molecules where a fork synthesising leading and lagging strands has not completely translocated along the template, as indicated by a previous 2D agarose electrophoresis analysis (see for example PMID: 35585232 extended data figure 8). Similar products are observed with the reconstituted budding yeast replisome.

2. Fig 2. Previous studies on circularized templates reported the Pol delta pauses on the G-rich lagging strand even in the absence of G4 folding (PMID: 24038470). Consistent with this, BLM helicase addition reduced, but did not appear to completely eliminate, stalled

products (Fig. 2f). Have the authors considered whether features of the G-rich sequence on the lagging strand beyond G4 folding may contribute to stalling?

Many thanks for raising this point, and our apologies this paper was not mentioned in the first submission. We agree that this is important to consider and in the revised manuscript, we have noted the previous study in the discussion and emphasised that other properties of G-rich telomeric DNA may feed into the inhibitory effect we observe.

3. Fig. 3. The text should include the rationale for the stoichiometric amounts of shelterin members used. Were equal molar amounts of each component added? This should also be added to the figure legend.

Apologies for the lack of clarity here – shelterin was added as a complex that was purified via a strep-tag on TIN2 from insect cells co-expressing all six subunits. We have expanded the methods to clarify this point and noted this in the results section. Please also see the points below.

How do the stoichiometric amounts added relate to the physiological concentrations of shelterin, which were reported to be unequal (i.e. not 1:1 ratios) (see PMID 19864690).

Indeed, not all shelterin subunits are likely to be expressed to the same level based on the paper cited and a previous mass spectrometry analysis (PMID: 26496610). The protein titrations included in our analysis aim to cover the likely concentrations of 40-50 nM TRF2 (60,000 per cell - PMID 19864690), roughly 2/3 this for TRF1 and 1/3 this for TPP1/POT1 (PMID: 26496610). Hence our titrations throughout the paper encompassing 5-45 nM. Please also see our reply to point 4, below.

Based on the amount of shelterin added and the number of telomeric repeats, do the authors predict that the telomeric sequences in the replication substrates will be fully bound by shelterin at the higher concentrations?

45 nM shelterin corresponds to around twenty shelterin molecules per template molecule. On the ‘short’ telomeric tract (360 bp), this would equate to one TRF2 dimer per 10-20 bp, which we assume is saturation or close to saturation. On the ‘long’ template, this would equate to one per 70-80 bp, which may be sub-saturation. These numbers are roughly in line with a previous estimate of shelterin density at telomeres in human cells (PMID 19864690).

Subcomplexes of shelterin have been described. Do the authors have evidence that the added proteins form the 6-member complex? Perhaps size exclusion chromatography could address the existence of complete and sub- complexes.

Thanks for this suggestion. We have used immunoblotting to explicitly show all six subunits are present in the complex (new figure EV3A). We have also included a mass photometry analysis of our purified shelterin (new figure EV3D), revealing a complex at 700 kDa, as well as several peaks at lower molecular weights. This profile is in line with a previous analysis (PMID: 35881804 – please note the peak at 780 kDa in this analysis includes MBP- and GFP

tags on several subunits) and suggests that even though the complex was purified including size exclusion chromatography, there are likely to be some subcomplexes present.

4. Fig. 3a. Do the hyphen marks point to the lane or represent "minus" symbols? Please clarify in the figure legend.

They are indeed 'minus' symbols. We have now changed our labelling to '0' instead of the minus symbol to avoid any confusion.

Why does TRF2 appear larger than its molecular weight (~60 kDa)? I would have expected that TRF1 and TRF2 would migrate the same given their similar molecular weight (see also PMID 19864690).

The TRF2 construct used here is the long isoform, hence why it runs above TRF1. We have now clarified this in the methods.

Based on the gel it does not appear that equal amounts of shelterin components are present (see previous comment).

Indeed, purifying shelterin via a strep-tag on TIN2 after co-expressing all six subunits, we consistently observe differences in the level of subunits. Quantifying the intensity of subunit bands from three separate shelterin preparations following SDS-PAGE, we observe a slight excess of TRF2 to TIN2, consistent with other studies (PMID: 35881804 and PMID: 29057866). We also observe less TPP1/POT1 than TIN2. This quantification is now included in figure EV3A.

5. Supplemental Fig 3a. For the EMSA, it would be helpful to show the wells of the gel to ensure that the bound complex migrated into the gel, rather than being retained in the well. Controls for non-telomeric DNA should be included to ensure any effects are due to shelterin binding to the telomeric sequences in the replication model.

Many thanks for this suggestion. We have repeated the EMSA with a shorter tract of telomeric repeats, which now runs fully into the gel (new Fig EV3B). This new analysis also includes competition experiments with unlabelled telomeric or non-telomeric DNA (new figure EV3C), showing that shelterin is indeed binding to DNA in a sequence-specific manner as expected.

Fig. 4. The conclusion that fork stalling and the induction of gaps are separate events is counterintuitive. Could fork uncoupling due to stalling lead to gaps on the lagging strand that are then bound by POT1?

At low shelterin concentrations, forks do not stall but replication products are still sensitive to P1 nuclease (figure 4E), which we interpret as showing persistent stalling is not required for gap formation per se. It is possible that gaps are formed differently on stalled and non-stalled molecules (e.g. stalling promotes gap formation in the way suggested) - the main point we wanted to make here was that irreversible stalling is not required for the inhibitory

effect of shelterin on the lagging strand. We have changed the wording of this section to better reflect this focus.

7. Have the authors considered whether BLM, in addition to RTEL1, might be able to displace shelterin? Previous reports indicate that BLM can displace RAD51 from DNA (PMID: 18003860). Therefore, it may be worth testing other helicases that prevent telomere fragility (i.e. BLM, FANCJ, WRN), based on the biochemical data presented that shelterin can stall forks.

Many thanks for the suggestion. We have now purified helicase active FANCJ and WRN in addition to BLM (new figure EV7F) and examined their ability to overcome the shelterin stall as requested. New figure EV7G shows that none are able to prevent the stall.

Minor comment

1. The authors have included much detail regarding protein purification. But it would be useful to include which subunits of Pol delta (3 or 4 subunits) were included. Was the shelterin complex purified from human or insect cells?

We have now clarified this in the methods

Referee #3:

Summary:

Major Comments:

1. Telomere replication studies have been waiting for a long time for a reconstituted system that allows scientists to carefully tease out the various mechanisms in place for the replisome to navigate the repetitive DNA landscape. Thus, we really appreciate and recognize the power of this work's approach and insights.

Many thanks for the positive input.

There are several interesting observations, some consistent with cellular findings and some are not. That's expected with a cutting-edge study like this, but our enthusiasm is much dimmed when the tracked signal (stalled band) is small and relatively weaker than the unperturbed signal (full length products). Most, if not all, of the authors' analysis and conclusions are based on a faint signal.

The stall signal is weak on face value, but this does not indicate a low penetrance effect: as is standard in the replication field, we use the incorporation of radiolabelled nucleotide (α-^{32}P-labelled dCTP) to follow DNA synthesis. This means that smaller products (e.g. from stalled leading strands) will contain less radiolabel per molecule. Thus, comparing the intensity of full-length and stalled bands directly does not reflect the fraction of forks that are stalled – product length must be taken into account. To overcome this, we included a normalised quantification of the percentage of forks that are stalled in figure 3a of our first submission (now figure 3B), revealing that around 60 % are arrested with 45 nM shelterin.

The new quantifications throughout the revised manuscript are in good agreement with this figure.

Our concern is further compounded with the current qualitative treatment and not using a quantitative statistical analysis of the stalled products (p-value across comparison, quantification of key bands, etc.). The authors should show statistical analysis (p-values should be provided) derived from quantification across three separate biochemical replicates for all experiments.

We agree that we did not go far enough in quantifying the data in our first submission and thank the reviewer for raising this point. We have now included quantifications and P-values for our data as requested, except in figure 2, where the distribution of defective lagging strand products on telomeric DNA was difficult to quantify accurately – for these data we have instead shown lane scans in triplicate (see Appendix I).

2. It is unclear if the FL signal in the gels were from unperturbed replisome products or from both leading and lagging strand synthesis. It will be helpful if the authors can offer more information to how the experimental signals were interpretated.

Certainly. In replication assays lacking FEN1 and LIG1, the full-length signal results from leading strand synthesis across the length of the template, as indicated by reactions lacking Pol α/primase ±CMG helicase such as in new figure 3B. When Pol α/primase is also included in these reactions, unligated Okazaki fragments run as small products that are variable in length but measure 200-400 nt such as in figure 2B, lane 1. This is a well-established profile of products from in vitro replication reactions in various reconstituted systems (e.g. PMID: 35585232, PMID: 24997598, PMID: 25739503, PMID: 27989442). In the presence of FEN1 and LIG1, the full-length signal comes from leading strands that have reached the end of the template and lagging strands that have been completely ligated together, as shown by the nickase experiments in Figs. EV2E and F.

3. The authors have used a 1.6 kb size telomeric repeat tract as a telomeric template model and most interpretations for stalled products are perceived to stem from the telomeric insert. Have the authors tested moving the relative position of the telomeric insert and see a corresponding change in the size of the stalled band?

As requested, we have now moved the position of the telomeric insert (which is 360 bp for the shelterin-stall experiments). We do indeed observe a corresponding change in the size of the stalled band (new Figure EV4C), consistent with our interpretation that it stems from shelterin-bound telomeric DNA.

4. Have the authors tested a larger telomeric insert that would better recapitulate the cellular telomere length? Perhaps, a longer telomeric tract will result in more stalled products over full-length products and lead to easier quantification versus a faint band quantification.

Indeed, we would be very keen to examine a longer telomeric insert. Unfortunately, we have found large tracts of telomeric DNA extremely challenging to work with and highly

unstable in bacteria, even those that are HR defective and grown at 30˚C. We have therefore been unable to address this point.

5. Do the authors think that the replisome was stalled at the onset of arriving at the telomeric region or the stalling was evenly distributed within the telomeric repeats? We think a clarification of the stalling distribution would be an important information for the field to understand how various mitigating or non-mitigating factors work.

Many thanks for this suggestion. We agree that this is an important point and have now analysed our shelterin-stalled products on UREA-PAGE sequencing gels, which reveal that leading strand synthesis stalls within the first 30-50 bp of telomeric DNA (new figure 3C), suggesting the replisome is arrested towards the beginning of the telomeric insert.

6. The manuscript writing needs improvements. Often, the rationale and motivation for an experiment condition were not provided before the data is described. This makes it tedious to read and especially for a reader to appreciate the result interpretation. For example, in page 9, last paragraph, the authors asked if CST can "overcome" (maybe alleviate or suppress is a better action verb?) the inhibitory effect of POT1.

Thanks for the input and the rewording suggestion. We have been through the manuscript in detail and extensively rewritten large sections to try and provide a clearer rationale and motivation for experiments.

Then the authors described they used a reduced RPA concentration. Our question is why do they need to reduce the RPA concentration for this test? Also, what happens if they kept RPA at the same concentration as the rest of the paper?

Our apologies - this experiment was not properly explained. In new figure EV7B, we show that while CST prevents the inhibitory effect of POT1 at the lower RPA concentration of 30 nM, this is not the case at the ‘standard’ RPA concentration of 100 nM. We think that CST may be unable to effectively compete with high RPA concentrations for binding to ssDNA in our reactions, necessitating the reduction. This may be different if we were to include the recently reported phospho-enhancement of the CST-TPP1/POT1 interaction (PMID: 38838667) into our reactions, which is an important future aim.

We think having someone outside the field of telomere and DNA replication to read will be extremely helpful in highlighting missing context or background information.

Thank you for the suggestion. As mentioned above, we have now been through the manuscript in detail and provided more context or background information where possible with input from colleagues. We hope that the revised version of the manuscript is easier to understand.

7. Manuscript organization: Fig 1B is skipped in the text and comes after Fig 1C is described. Also, the description of the telomeric DNA fork construct came late in page 4, 2nd paragraph. This description should be introduced earlier in that result section.

Many thanks for the suggestion. The figures have been reorganised in the revised manuscript, which we think has solved this problem.

8. Figure 1d/manuscript organization: Page 4, paragraph 2 states that a pulse chase assay was used. The figure caption for figure 1 states that a chase was used. Nowhere does it state what the pulse or the chase was in a concise way for the reader. This should be made immediately clear when introducing the experiment/figure. It would be helpful if this experiment (and others) were laid out in such a way that the reader would not need to go searching all the way to the last sentence of page 31 in the methods for essential information.

Thanks for the suggestion. In the legend for figure 1D, we have now noted that the chase constituted an excess of unlabelled dNTPs, which we hope will make it easier to understand.

Additionally, have the authors quantified the bulk replication rate after the pulse?

We have measured the corresponding bulk replication rates and now report these in figure EV1C. Consistent with the analysis of the fastest forks, there is no significant difference in the bulk replication rate on the telomeric and control templates, in line with our conclusion that the telomeric repeats in either orientation are efficiently replicated as the leading strand.

9. Fig. S2a lane 2 showed the products made by the addition of Pola-primase only. However, the DNA markers in the gel do not provide the appropriate range to assess the size range of primers made by Pola-primase. Based on the available marker bands, it seems the enzyme is making primers of several hundred nucleotides long. Are these just RNA-DNA primers made by Pola-primase or extended primers by other added polymerase? RNA-DNA primers made by Pola-primase are typically ~30 nt in size which would contradicts the authors interpretation of their gel signal. Also, We are not convinced that there is a significant change in the size of the OF bands (smear) after DNA polymerase delta addition. This point here also emphasizes on the need to carefully quantify these perceived changes described by the authors.

Several previous studies have shown that purified Pol α/primase can in fact generate DNA products kilobases in length over the timescales and protein concentrations used here (see for example Figure 6a in PMID: 29944888). Indeed, in the absence of other polymerases, budding yeast Pol α/primase can synthesise OFs hundreds of nucleotides long behind CMG and even leading strands kilobases in length, consistent with our interpretation (see Fig. 2 of PMID: 25871847). It is possible that Pol ε contributes to some extent but a previous study has shown that this is unlikely to be the case in the presence of Pol δ, which is dominant over Pol ε on a reconstituted lagging strand system with budding yeast proteins (PMID: 24997598).

For OF lengthening by Pol δ, we consistently see an increase in OF length in the presence of Pol δ but the magnitude of this increase can vary somewhat. The lengthening in this experiment was indeed more subtle than we routinely observe - we have now repeated the experiment and replaced it in figure EV2A.

10. A major concern is the QC for recombinant shelterin complexes used in this work. The authors assigned the identity of protein bands in a SDS-PAGE in fig 3a. The largest band is assigned as TRF2 but past studies including similar analysis showed POT1 migrates slower than TRF2 and TPP1. Here, the authors assigned POT1 and TPP1 as similar migration size. We understand that depending on the tags and gel buffer system used, the migration rate of a protein can be different. Thus we ask if the authors have verified their band assignment using western blot? If yes, we think they should be provided.

The slower migration of TRF2 and the similar migration pattern of POT1 and TPP1 are likely due to the fact we used the longer isoforms of TRF2 and TPP1 here. We have now noted the fact that the longer isoforms were used in the methods and have confirmed that both the long and short isoforms of TPP1 support the inhibitory effect of POT1 (see reviewer figure below). Please note the loss of expected bands with different shelterin subcomplexes (now figure EV5A) provides evidence for the band assignments we have made. We have also included an immunoblot analysis of two independently purified shelterin preparations in figure EV3A, confirming consistency of subunits across preparations.

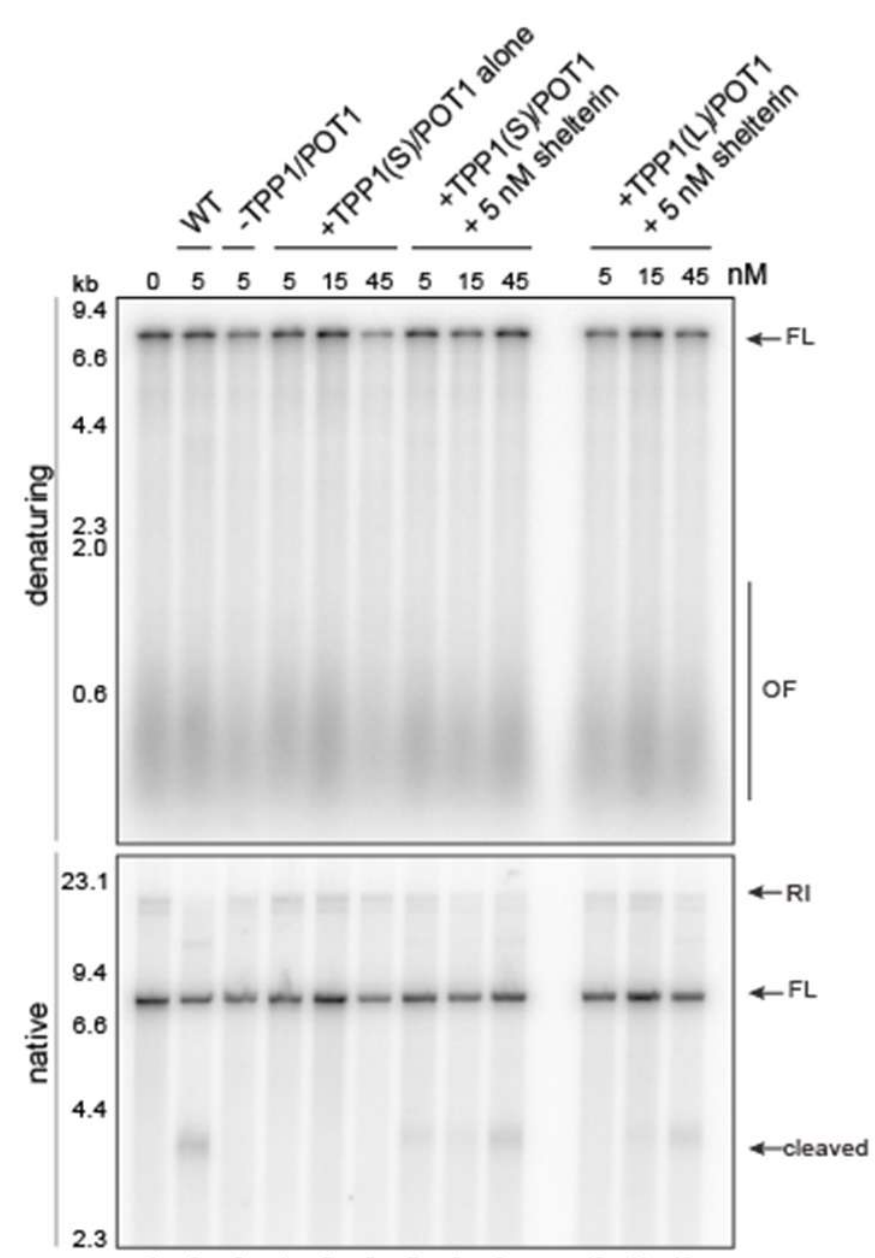

Figure: Replication reactions performed for 15 min with a template containing 60 TTAGGG repeats and either 5 nM wt shelterin, 5 nM shelterin -TPP1/POT1, or the indicated concentrations of TPP1(short isoform)/POT1 alone or preincubated with shelterin -TPP1/POT1. TPP1(long isoform)/POT1 preincubated with shelterin -TPP1/POT1 is shown for comparison. 0.24 U/μl P1 nuclease was added 5 min before quenching reactions. Products were analysed by native and denaturing agarose electrophoresis. Equivalent levels of P1 cleavage in lanes 9 and 12 reveal the short and long TPP1 isoforms are equally able to support the inhibitory effect of POT1 on the lagging strand.

11. Figure 3: As mentioned before, the faint stall bands should be quantified with replicates. This is important evidence for a reader to be convinced of the authors' conclusions. For

example, in Fig 3B, the authors claim that "forks arrested on the telomere within the first five minutes remained there for at least a further 55 minutes, suggesting the replisome alone is ineffective at bypassing shelterin once a stall has taken place". The authors should quantify the stall band and provide an explanation for the increase in signal of the full-length band.

We have performed this analysis as requested (new Figure EV4A). The stall band is not significantly different across the time course, supporting our conclusion that the replisome cannot efficiently bypass shelterin in the absence of additional factors. The increase in the full length is also observed in the absence of shelterin revealing it is not related to shelterin bypass. While the source of this increase is currently unclear, our working model is that it is caused by replication intermediates resolving into full length products over time.

A second example is Figure 3C, which the authors use to claim that "Shelterin was equally effective at stalling the replisome when either C- or G-rich telomeric DNA was the leading strand template." Again, the authors do not quantify the stall or full-length bands used to make this conclusion. Have the authors tested this conclusion with an additional data point between 25 and 45 nM shelterin?

We have repeated this experiment with an intermediate shelterin concentration as requested (new Figure EV4E) and quantified the result, confirming our conclusion that there is no difference in the ability to stall the replisome on telomeric DNA in one orientation or the other.

12. It was not clear which shelterin complex was used for the EMSA shown in fig S3A. We assumed it is the largest 6 subunit shelterin complex. If yes, have the authors tested the complex stoichiometry?

Yes, we used the 6-subunit complex here and have now noted this in the figure legend. We have also analysed this complex by mass photometry (new figure EV3D), revealing a peak at 700 kDa as well as several smaller peaks likely to derive from subcomplexes. This analysis is consistent with a previous mass photometry examination of purified shelterin (PMID: 35881804 – please note the peak at 780 kDa in this analysis includes MBP- and GFP tags on several subunits). While the presence of the subcomplexes makes it challenging to unambiguously assign a stoichiometry to the complex, we have quantified the intensity of different subunit bands in three separate shelterin preparations, following SDS-PAGE conditions where we are able to separate all subunits except TPP1 and POT1 (new figure EV3A). We observe an excess of TRF2 to TIN2 when shelterin is purified via a strep-tag on TIN2, consistent with previous studies (PMID: 35881804 and PMID: 29057866). We also observe less TPP1/POT1 relative to the other shelterin subunits.

a 360 bp telomeric DNA is a poor substrate to use for checking if their purified shelterin can bind specifically to telomeric DNA. The larger than necessary substrate complicates binding affinity quantification and likely also resulted in a seemingly well-shift situation. Achieving a gel-shift condition is important for proper binding analysis. Additionally, the authors should provide data to confirm that shelterin does not bind their control DNA.

We have repeated the EMSA with a short telomeric tract which is now well resolved by agarose electrophoresis (new Figure EV3B). We have also performed a competition experiment to show that shelterin is binding specifically to telomeric DNA (see new figure EV3C). Thanks for the suggestions.

13. Fig 3e: Did the authors test if adding only TRF1 or TRF2 or TIN2-TRF1-TRF2 leads to the same stalling effect, as predicted by their interpretation?

We have now tested TRF1 and TRF2 and TIN2-TRF1-TRF2 for their ability to stall the replisome (see new figure EV5D). They are indeed sufficient, consistent with our interpretation that the stall is induced by their interaction with telomeric DNA. Our working model is that the slightly lower stalling efficiency of TRF1 and TRF2 alone (figure EV5D) may reflect their reduced size compared to the full complex.

14. Fig 4a: The authors reference figure 4a on page 7-8 as if it contains data with and without shelterin. However, only data with shelterin is provided, while the data without shelterin is not presented in figures 4 or S4. This is confusing for the reader. This figure should be improved so that the quantitation is clearly visually shown in a single, unified figure, without the need for such a complicated textual explanation in the figure reference.

We observe very few forks without shelterin, hence why we did not include this condition in original Fig. 4a. Taking into account the suggestion from the reviewer, we have unified the figure and tried to simplify the description (which we agree was cumbersome). Thanks for the input and suggestion.

15. Methods: The organization of the methods section could use some clarification. In some cases, additional break points into new paragraphs will be enough to make clear which reaction is being described. In other cases, additional clarification is needed. For example, page 31 lines 5 and 6, ranges are provided for the concentration of RPA and RFC in the in vitro reactions, but it is not made clear enough in the manuscript why these are ranges or what concentration was used in each experiment.

Apologies. The ranges were there as placeholders while we finalised the manuscript and remained in error. They have now been changed to the actual values used. We have also reformatted parts of the methods, which we hope has made them easier to understand.

Minor Comments:
1. Figure S1A should be included in main figure 1, as it provides essential context for the work.

We have now included this in figure 1 as requested.

2. Page 5, first paragraph: OF synthesis. We may have missed the initial definition for this acronym. If not, please provide the full name definition if this is used for the first time in the text.

Apologies, the initial definition was omitted in error when the manuscript was prepared but has now been corrected. Thanks for pointing this out.

3. Figure 3A should be split into three separate panels.

This has been done.

4. Figure 3E: What does TRF1/2 myb domain deletion mean? Both TRF1 and TRF2 deletion?

Indeed – the myb domains of both TRF1 and TRF2 have been deleted.

5. Nicking/gapping site annotation: The gaps used in the figures to denote gaps (2A) or nicks (4C) in the DNA are rather small, it might be helpful to make the overall picture larger or to make these sites more obvious in some other way (more colors, widened gaps, thicker line width?).

Thanks for the suggestion. This has been done

Note: This manuscript is co-reviewed with a member of a referee's laboratory as part of the mentoring process. The primary referee has independently evaluated the manuscript and agrees with the report filed.

Thanks to both the primary referee and the trainee for their comments.

Dr. Max E Douglas
The Institute of Cancer Research
Cell and Molecular Biology
237 Fulham Road
London SW3 6JB
United Kingdom

26th May 2026

Re: EMBOJ-2025-122482R
Inhibition of lagging strand replication by G-rich telomeric DNA and the shelterin subunit POT1

Dear Max,

Thank you for submitting your revised manuscript on telomeric replication stress for our consideration. Two of the original referees have now assessed it again and were fully satisfied with the revisions. We shall therefore be happy to accept the manuscript for publication, following incorporation of a few remaining editorial points as follows:

- Please adjust the order of the manuscript sections, and also make sure to use the correct section headers:
Single title page with complete author information, Abstract, Introduction, Results, Discussion, Methods, Data Availability, Acknowledgements, Disclosure and Competing Interests Statement, References, Main Figure Legends, Tables, Expanded Figure Legends.

- As we are switching from a free-text author contribution statement towards a more formal statement based on Contributor Role Taxonomy (CRediT) terms, please remove the present Author Contribution section and instead specify each author's contribution(s) directly in the Author Information page of our submission system during upload of the final manuscript. See https://casrai.org/credit/ for more information.

- Please adjust the format of the reference list and of the in-text citations according to EMBO Journal format (alphabetical order, author name et al + year, first up to 10 authors should be listed, followed by 'et al'), also check that all citations are complete with complete volume and page numbers (DOI info only in case of advance publications that do not yet have volume/page numbers).

- Please make sure that all figures (and figure panels) are called out in sequential order, and that each panel is called out at least once (every.g. Fig. 3D seems not the referenced at all now). If necessary, start by referencing a whole figure, e.g. Fig 9A-D, first.

- For collate the two Appendix files into a single Appendix PDF, which should be prefaced with a title page stating "Appendix for [manuscript title]", and include page numbers for the items listed in the ToC. The first item should be renamed into Appendix Figure S1, the second Appendix Text S1, and all references in the text updated accordingly.

- Please provide suggestions for a short 'blurb' text prefacing and summing up the conceptual aspect of the study in two sentences (max. 250 characters), followed by 3-5 one-sentence 'bullet points' with brief factual statements of key results of the paper; they will form the basis of an editor-written 'Synopsis' accompanying the online version of the article. You may also provide a dedicated synopsis image, with the modest dimensions of (exactly) 550 pixels wide and between 300 pixels high, but I think we could also simply re-display current Figure 6 for this purpose.

- Finally, during routine pre-acceptance checks, our data editors have raised the following queries regarding figures, data, and legends; I would appreciate if you briefly answered to them in the cover letter of your final submission, and made the requested text modifications with changes/additions highlighted via the "Track changes" option, to facilitate our final checking":
1. Please provide the exact p values in the legends of 3B; 4D,E; 5A,B,C,D; EV-2C; EV-3C; EV-5C,D; EV-7A,C.
2. Although 'n' is provided, please describe the nature of entity for 'n' in the legends of figures 1E; 3B; 4D,E; 5A,B,C,D; EV-1C; EV-2C; EV-3A,B,C; EV-4A,C,D,E; EV-5B,C,D; EV-7A,C,G.
3. Please define the scale bar for figure EV-6C.

I am returning the manuscript to you for a final round of minor revision, solely to allow you to make these modifications and upload the revised files. Once we will have received them, we should be ready to swiftly proceed with formal acceptance and production of the manuscript.

With kind regards,

Hartmut

Hartmut Vodermaier, PhD
Senior Editor, The EMBO Journal
h.vodermaier@embojournal.org

*** PLEASE NOTE: All revised manuscript are subject to initial checks for completeness and adherence to our formatting guidelines. Revisions may be returned to the authors and delayed in their editorial re-evaluation if they fail to comply to the following requirements. As a first step please read our guidelines for revised submissions:
https://link.springer.com/journal/44318/submission-guidelines#cms-Revised-submissions

1) Every manuscript requires a Data Availability section (even if only stating that no deposited datasets are included). Primary datasets or computer code produced in the current study have to be deposited in appropriate public repositories prior to resubmission, and reviewer access details provided in case that public access is not yet allowed.

2) Each figure legend must specify
- size of the scale bars that are mandatory for all micrograph panels
- the statistical test used to generate error bars and P-values
- the type error bars (e.g., S.E.M., S.D.)
- the number (n) and nature (biological or technical replicate) of independent experiments underlying each data point
- Figures may not include error bars for experiments with n<3; scatter plots showing individual data points should be used instead.

3) Revised manuscript text (including main tables, and figure legends for main and EV figures) has to be submitted as editable text file (e.g., .docx format). We encourage highlighting of changes (e.g., via text color) for the referees' reference.

4) Each main and each Expanded View (EV) figure should be uploaded as individual production-quality files (preferably in .eps, .tif, .jpg formats). For suggestions on figure preparation/layout, please refer to our Figure Preparation Guidelines:
https://media.springernature.com/original/springer-cms/rest/v1/content/27825798/data/v1

5) Point-by-point response letters should include the original referee comments in full together with your detailed responses to them (and to specific editor requests if applicable), and also be uploaded as editable (e.g., .docx) text files.

6) Please complete our Author Checklist, and make sure that information entered into the checklist is also reflected in the manuscript; the checklist will be available to readers as part of the Review Process File.

7) All authors listed as (co-)corresponding need to deposit, in their respective author profiles in our submission system, a unique ORCiD identifier linked to their name. Please see our Guide to Authors for detailed instructions.

8) Please note that supplementary information at EMBO Press has been superseded by the 'Expanded View' for inclusion of additional figures, tables, movies or datasets; with up to five EV Figures being typeset and directly accessible in the HTML version of the article.

9) To facilitate reproducibilty and cross-laboratory adoption of methodologies, please structure the Materials & Methods section as outlined in our guide to authors, including a completed Reagents and Tools Table.

10) Digital image enhancement is acceptable practice, as long as it accurately represents the original data and conforms to community standards. If a figure has been subjected to significant electronic manipulation, this must be clearly noted in the figure legend and/or the 'Materials and Methods' section. The editors reserve the right to request original versions of figures and the original images that were used to assemble the figure. Finally, we generally encourage uploading of numerical as well as gel/blot image source data.

At EMBO Press we ask authors to provide source data for the main manuscript figures. You will receive a separate email with instructions for providing source data with your revised manuscript, including how to upload and organize the files.

In the interest of ensuring the conceptual advance provided by the work, we recommend submitting a revision within 3 months (24th Aug 2026). Please discuss the revision progress ahead of this time with the editor if you require more time to complete the revisions. Use the link below to submit your revision:

Link Not Available

Referee #1:

The authors have addressed all the points I raised previously. I do not have any further comments for improvement.

Referee #2:

The authors have addressed all my prior concerns. I commend the authors on an outstanding study that represents an important advance in the field.

EMBO Press Author Checklist

Corresponding Author Name: Max Douglas	
Journal Submitted to: The EMBO Journal	
Manuscript Number: EMBOJ-2025-122482	

USEFUL LINKS FOR COMPLETING THIS FORM
The EMBO Journal - Author Guidelines
EMBO Reports - Author Guidelines
Molecular Systems Biology - Author Guidelines
EMBO Molecular Medicine - Author Guidelines

Reporting Checklist for Life Science Articles (updated January
This checklist is adapted from Materials Design Analysis Reporting (MDAR) Checklist for Authors. MDAR establishes a minimum set of requirements in transparent reporting in the life sciences (see Statement of Task: 10.31222/osf.io/9sm4x). Please follow the journal's guidelines in preparing your
Please note that a copy of this checklist will be published alongside your article.

Abridged guidelines for figures
1. Data
The data shown in figures should satisfy the following conditions:
- the data were obtained and processed according to the field's best practice and are presented to reflect the results of the experiments in an accurate and unbiased manner.
- ideally, figure panels should include only measurements that are directly comparable to each other and obtained with the same assay.
- plots include clearly labeled error bars for independent experiments and sample sizes. Unless justified, error bars should not be shown for technical
- if n<5, the individual data points from each experiment should be plotted. Any statistical test employed should be justified.
- Source Data should be included to report the data underlying figures according to the guidelines set out in the authorship guidelines on Data

2. Captions
Each figure caption should contain the following information, for each panel where they are relevant:
- a specification of the experimental system investigated (eg cell line, species name).
- the assay(s) and method(s) used to carry out the reported observations and measurements.
- an explicit mention of the biological and chemical entity(ies) that are being measured.
- an explicit mention of the biological and chemical entity(ies) that are altered/varied/perturbed in a controlled manner.
- the exact sample size (n) for each experimental group/condition, given as a number, not a range;
- a description of the sample collection allowing the reader to understand whether the samples represent technical or biological replicates (including how many animals, litters, cultures, etc.).
- a statement of how many times the experiment shown was independently replicated in the laboratory.
- definitions of statistical methods and measures:
  - common tests, such as t-test (please specify whether paired vs. unpaired), simple $\chi 2$ tests, Wilcoxon and Mann-Whitney tests, can be unambiguously identified by name only, but more complex techniques should be described in the methods section;
  - are tests one-sided or two-sided?
  - are there adjustments for multiple comparisons?
  - exact statistical test results, e.g., P values = x but not P values < x;
  - definition of 'center values' as median or average;
  - definition of error bars as s.d. or s.e.m.

Please complete ALL of the questions below.
Select "Not Applicable" only when the requested information is not relevant for your study.

Materials

Newly Created Materials	Information included in the manuscript?	In which section is the information available? (Reagents and Tools Table, Materials and Methods, Figures, Data Availability Section)
New materials and reagents need to be available; do any restrictions apply?	Not Applicable	

Antibodies	Information included in the manuscript?	In which section is the information available? (Reagents and Tools Table, Materials and Methods, Figures, Data Availability Section)
For **antibodies** provide the following information:<br>- Commercial antibodies: RRID (if possible) or supplier name, catalogue number and or/clone number<br>- Non-commercial: RRID or citation	Yes	In the structured methods

DNA and RNA sequences	Information included in the manuscript?	In which section is the information available? (Reagents and Tools Table, Materials and Methods, Figures, Data Availability Section)
**Short novel DNA or RNA including primers, probes**: provide the sequences.	Yes	in the structured methods

Cell materials	Information included in the manuscript?	In which section is the information available? (Reagents and Tools Table, Materials and Methods, Figures, Data Availability Section)
**Cell lines:** Provide species information, strain. Provide accession number in repository **OR** supplier name, catalog number, clone number, and/**OR** RRID.	Not Applicable	
**Primary cultures:** Provide species, strain, sex of origin, genetic modification status.	Not Applicable	
Report if the cell lines were recently **authenticated** (e.g., by STR profiling) and tested for mycoplasma contamination.	Not Applicable	

Experimental animals	Information included in the manuscript?	In which section is the information available? (Reagents and Tools Table, Materials and Methods, Figures, Data Availability Section)
**Laboratory animals or Model organisms:** Provide species, strain, sex, age, genetic modification status. Provide accession number in repository **OR** supplier name, catalog number, clone number, **OR** RRID.	Not Applicable	
**Animal observed in or captured from the field:** Provide species, sex, and age where possible.	Not Applicable	
Please detail **housing and husbandry conditions**.	Not Applicable	

Plants and microbes	Information included in the manuscript?	In which section is the information available? (Reagents and Tools Table, Materials and Methods, Figures, Data Availability Section)
**Plants:** provide species and strain, ecotype and cultivar where relevant, unique accession number if available, and source (including location for collected wild specimens).	Not Applicable	
**Microbes:** provide species and strain, unique accession number if available, and source.	Not Applicable	

Human research participants	Information included in the manuscript?	In which section is the information available? (Reagents and Tools Table, Materials and Methods, Figures, Data Availability Section)
If collected and within the bounds of privacy constraints report on age, sex and gender or ethnicity for all study participants.	Not Applicable	

Core facilities	Information included in the manuscript?	In which section is the information available? (Reagents and Tools Table, Materials and Methods, Figures, Data Availability Section)
If your work benefited from core facilities, was their service mentioned in the acknowledgments section?	Not Applicable	

Design

Study protocol	Information included in the manuscript?	In which section is the information available? (Reagents and Tools Table, Materials and Methods, Figures, Data Availability Section)
If study protocol has been **pre-registered, provide DOI in the manuscript**. For clinical trials, provide the trial registration number **OR** cite DOI.	Not Applicable	
Report the **clinical trial registration number** (at ClinicalTrials.gov or equivalent), where applicable.	Not Applicable	

Laboratory protocol	Information included in the manuscript?	In which section is the information available? (Reagents and Tools Table, Materials and Methods, Figures, Data Availability Section)
Provide DOI OR other citation details if **external detailed step-by-step protocols** are available.	Not Applicable	

Experimental study design and statistics	Information included in the manuscript?	In which section is the information available? (Reagents and Tools Table, Materials and Methods, Figures, Data Availability Section)
Include a statement about **sample size** estimate even if no statistical methods were used.	Yes	sample size is noted in the legend for each figure
Were any steps taken to minimize the effects of subjective bias when allocating animals/samples to treatment (e.g. **randomization procedure**)? If yes, have they been described?	Yes	blinding of EM samples for analysis is noted in the methods
Include a statement about **blinding** even if no blinding was done.	Yes	noted in the methods
Describe **inclusion/exclusion criteria** if samples or animals were excluded from the analysis. Were the criteria pre-established?<br><br>If sample or data points were omitted from analysis, report if this was due to attrition or intentional exclusion and provide justification.	Not Applicable	
For every figure, are **statistical tests** justified as appropriate? Do the data meet the assumptions of the tests (e.g., normal distribution)? Describe any methods used to assess it. Is there an estimate of variation within each group of data? **Is** the variance similar between the groups that are being statistically compared?	Yes	information on statistical tests and P-values are noted in the legend to each figure

Sample definition and in-laboratory replication	Information included in the manuscript?	In which section is the information available? (Reagents and Tools Table, Materials and Methods, Figures, Data Availability Section)
In the figure legends: state number of times the experiment was **replicated** in laboratory.	Yes	in the figure legends
In the figure legends: define whether data describe **technical or biological replicates**.	Not Applicable	

Ethics

Ethics	Information included in the manuscript?	In which section is the information available? (Reagents and Tools Table, Materials and Methods, Figures, Data Availability Section)
Studies involving **human participants**: State details of **authority granting ethics approval** (IRB or equivalent committee(s), provide reference number for approval.	Not Applicable	
Studies involving **human participants**: Include a statement confirming that **informed consent** was obtained from all subjects and that the experiments conformed to the principles set out in the WMA Declaration of Helsinki and the Department of Health and Human Services Belmont Report.	Not Applicable	
Studies involving **human participants:** For publication of **patient photos**, include a statement confirming that consent to publish was obtained.	Not Applicable	
Studies involving experimental **animals**: State details of **authority granting ethics approval** (IRB or equivalent committee(s), provide reference number for approval. Include a statement of compliance with ethical regulations.	Not Applicable	
Studies involving **specimen and field samples:** State if relevant **permits** obtained, provide details of authority approving study; if none were required, explain why.	Not Applicable	

Dual Use Research of Concern (DURC)	Information included in the manuscript?	In which section is the information available? (Reagents and Tools Table, Materials and Methods, Figures, Data Availability Section)
Could your study fall under dual use research restrictions? Please check biosecurity documents and list of **select agents and toxins** (CDC): https://www.selectagents.gov/sat/list.htm	Not Applicable	
If you used a select agent, is the security level of the lab appropriate and reported in the manuscript?	Not Applicable	
If a study is subject to dual use research of concern regulations, is the name of the **authority granting approval and reference number** for the regulatory approval provided in the manuscript?	Not Applicable	

Reporting

The MDAR framework recommends adoption of discipline-specific guidelines, established and endorsed through community initiatives. Journals have their own policy about requiring specific guidelines and recommendations to complement MDAR.

Adherence to community standards	Information included in the manuscript?	In which section is the information available? (Reagents and Tools Table, Materials and Methods, Figures, Data Availability Section)
State if relevant guidelines or checklists (e.g., **ICMJE, MIBBI, ARRIVE, PRISMA**) have been followed or provided.	Not Applicable	
For **tumor marker prognostic studies,** we recommend that you follow the **REMARK** reporting guidelines (see link list at top right). See author guidelines, under 'Reporting Guidelines'. Please confirm you have followed these guidelines.	Not Applicable	
For **phase II and III randomized controlled trials**, please refer to the **CONSORT** flow diagram (see link list at top right) and submit the CONSORT checklist (see link list at top right) with your submission. See author guidelines, under 'Reporting Guidelines'. Please confirm you have submitted this list.	Not Applicable	

Data Availability

Data availability	Information included in the manuscript?	In which section is the information available? (Reagents and Tools Table, Materials and Methods, Figures, Data Availability Section)
Have **primary datasets** been deposited according to the journal's guidelines (see 'Data Deposition' section) and the respective accession numbers provided in the Data Availability Section?	Not Applicable	
Were **human clinical and genomic datasets** deposited in a public access-controlled repository in accordance to ethical obligations to the patients and to the applicable consent agreement?	Not Applicable	
Are **computational models** that are central and integral to a study available without restrictions in a machine-readable form? Were the relevant accession numbers or links provided?	Not Applicable	
If publicly available data were reused, provide the respective **data citations in the reference list**.	Not Applicable	
