# Supplementary material for: Inhibition of lagging strand replication by G-rich telomeric DNA and the shelterin subunit POT1
Source: EMBO J. 2026 Jul 9;45(16):5793–826. doi: 10.1038/s44318-026-00844-7 (PMC13483900; doi:10.1038/s44318-026-00844-7)
Supplement: Supplementary file 3 [file 44318_2026_844_MOESM8_ESM.pdf]

Expanded View Figures

Figure EV1. Supplementary data for Fig. 1.

(**A**) Quantification of the maximal leading strand synthesis rates from denaturing alkaline agarose electrophoresis. Linear regression is fit to the average of three independent experiments and error bars show S.E.M. (**B**) Replication reactions performed with non-telomeric control template for the indicated times with proteins omitted as indicated. Products analysed by denaturing alkaline agarose electrophoresis. (**C**) Quantification of bulk fork rates from pulse chase reactions as in Fig. 1D,G. Error bars show S.E.M. $N = 3$ (three independent replication assays). Unpaired *t* test used to determine statistical significance. Corresponding *P* values: control vs telomeric C-rich Lead (0.51), control vs telomeric G-rich Lead (0.99). (**D**) Pulse chase analysis with a template containing 240 TTAGGG repeats or a non-telomeric control sequence. Chase of non-radiolabelled dNTPs was added after 50 s. Samples taken at the indicated times after initiation were analysed by native and denaturing alkaline agarose electrophoresis (**E**) As for (**D**), except comparing a template containing a non-telomeric control or 240 TTAGGG repeats oriented with G-rich telomeric DNA as the sequence for leading strand replication. (**F**) Products from replication reactions performed for 15 min with templates containing a non-telomeric control sequence or 240 TTAGGG repeats where G-rich telomeric DNA is the sequence for leading strand replication and with the indicated concentrations of phenDC3 were analysed as in (**B**). (**G**) As for (**E**), except comparing a template containing a non-telomeric control sequence or 240 TTAGGG repeats where C-rich telomeric DNA is the sequence for leading strand replication.

 © The Author(s)

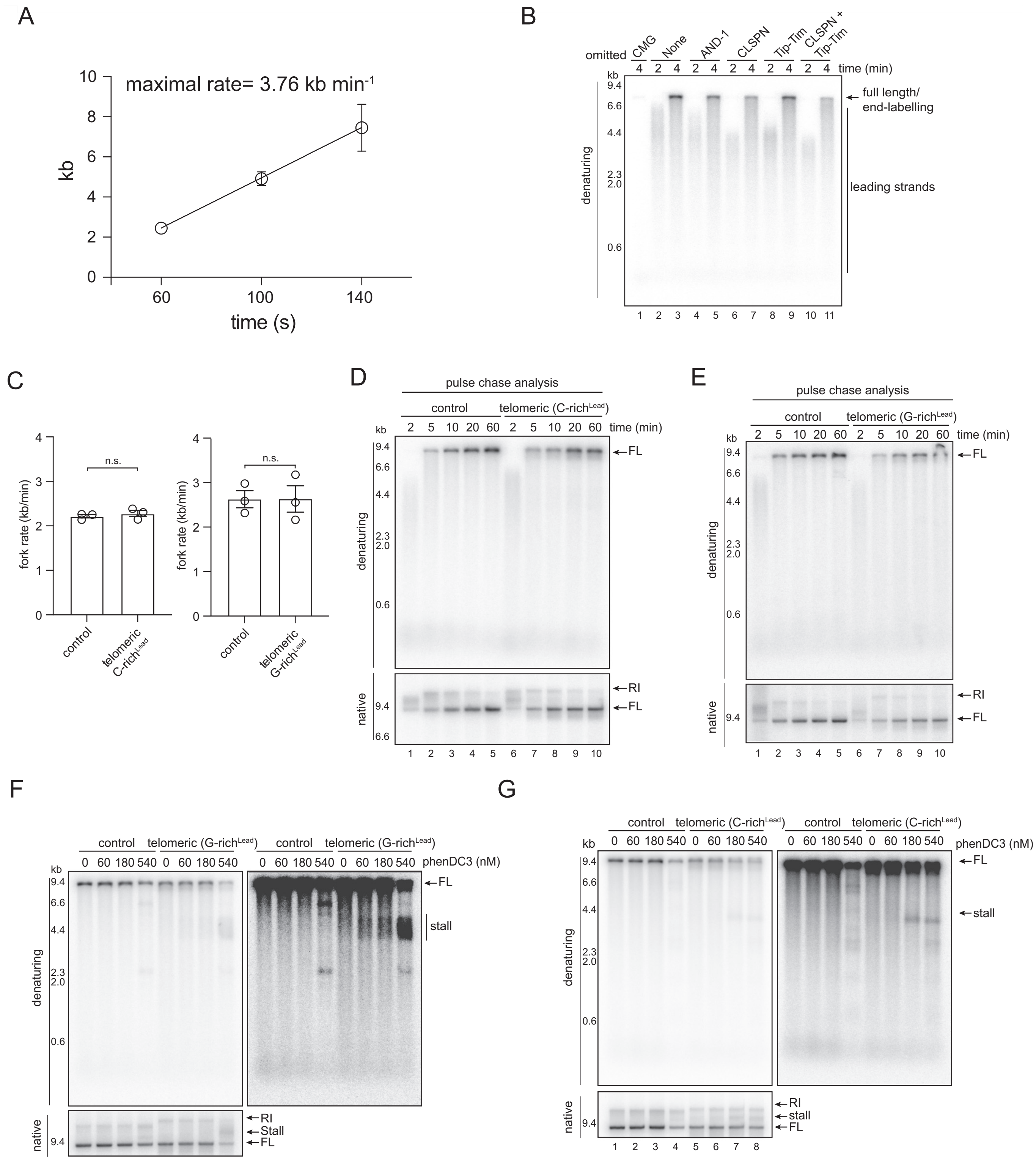
A
maximal rate= 3.76 kb min-1
kb
time (s)
B
omitted CMG None AND-1 CLSPN Tip-Tim CLSPN + Tip-Tim
time (min)
denaturing
full length/ end-labelling
leading strands
C
fork rate (kb/min)
n.s.
control
telomeric C-richLead
telomeric G-richLead
D
pulse chase analysis
control telomeric (C-richLead)
time (min)
denaturing
native
FL
RI
E
pulse chase analysis
control telomeric (G-richLead)
time (min)
F
control telomeric (G-richLead)
phenDC3 (nM)
stall
Stall
G
control telomeric (C-richLead)
phenDC3 (nM)
stall

© The Author(s)

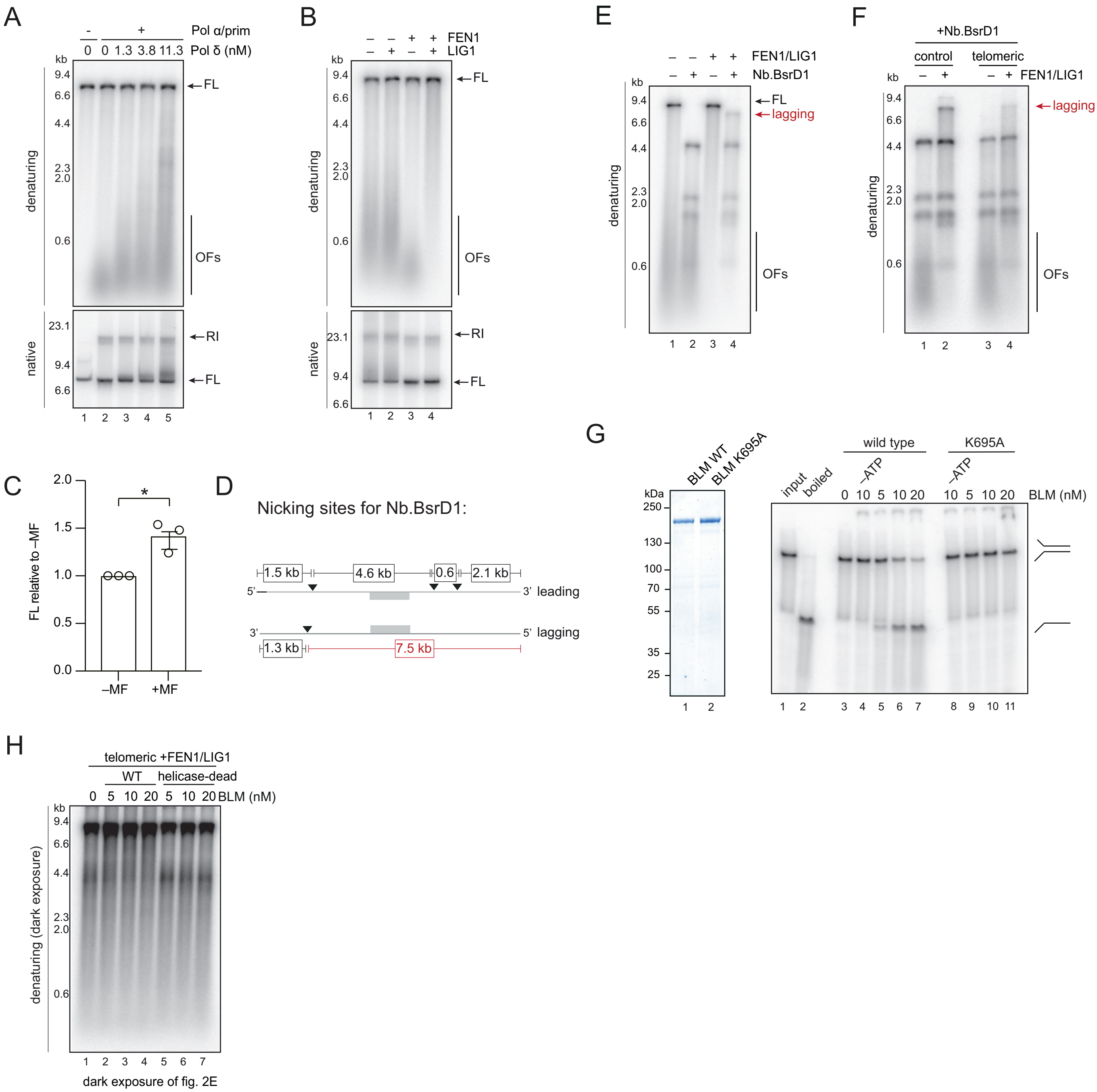

Figure EV2. Supplementary data for Fig. 2.

(A) Replication reactions performed for 20 min with a non-telomeric control template in the presence or absence of the indicated proteins. Products were analysed by native and denaturing alkaline agarose electrophoresis as indicated. **(B)** Products from replication reactions performed for 30 min with a non-telomeric control template in the presence or absence of FEN1 or LIG1 as indicated were analysed as in A. **(C)** Quantification showing full length signal in the presence of maturation factors (MF) relative to their absence. Error bars show the S.E.M. $N = 3$ (three independent replication assays). Unpaired *t* test used to determine statistical significance ($P = 0.0101$). **(D)** Schematic shows position of Nb.BsrDI sites and products expected from nicking of leading and lagging strand products. **(E)** Products from replication reactions performed for 30 min with a non-telomeric control template in the presence or absence of FEN1/LIG1 as indicated were treated with Nb.BsrDI for 2 h and analysed by denaturing alkaline agarose electrophoresis. Position of product indicating fully-matured lagging strand is shown. **(F)** Products from replication reactions performed for 30 min with templates containing 240 TTAGGG repeats or a non-telomeric control sequence in the presence or absence of FEN1/LIG1 as indicated were treated with Nb.BsrDI for 2 h and analysed by denaturing alkaline agarose electrophoresis. **(G)** 4–15% Tris-Glycine SDS-PAGE analysis of purified wild-type ('wt') and helicase-dead (K695A) BLM. Products from helicase assays performed for 10 min on 1.5 nM duplexed substrate with the indicated concentrations of BLM wt or helicase dead were analysed by native agarose electrophoresis. **(H)** Dark exposure of Fig. 2E.

 © The Author(s)

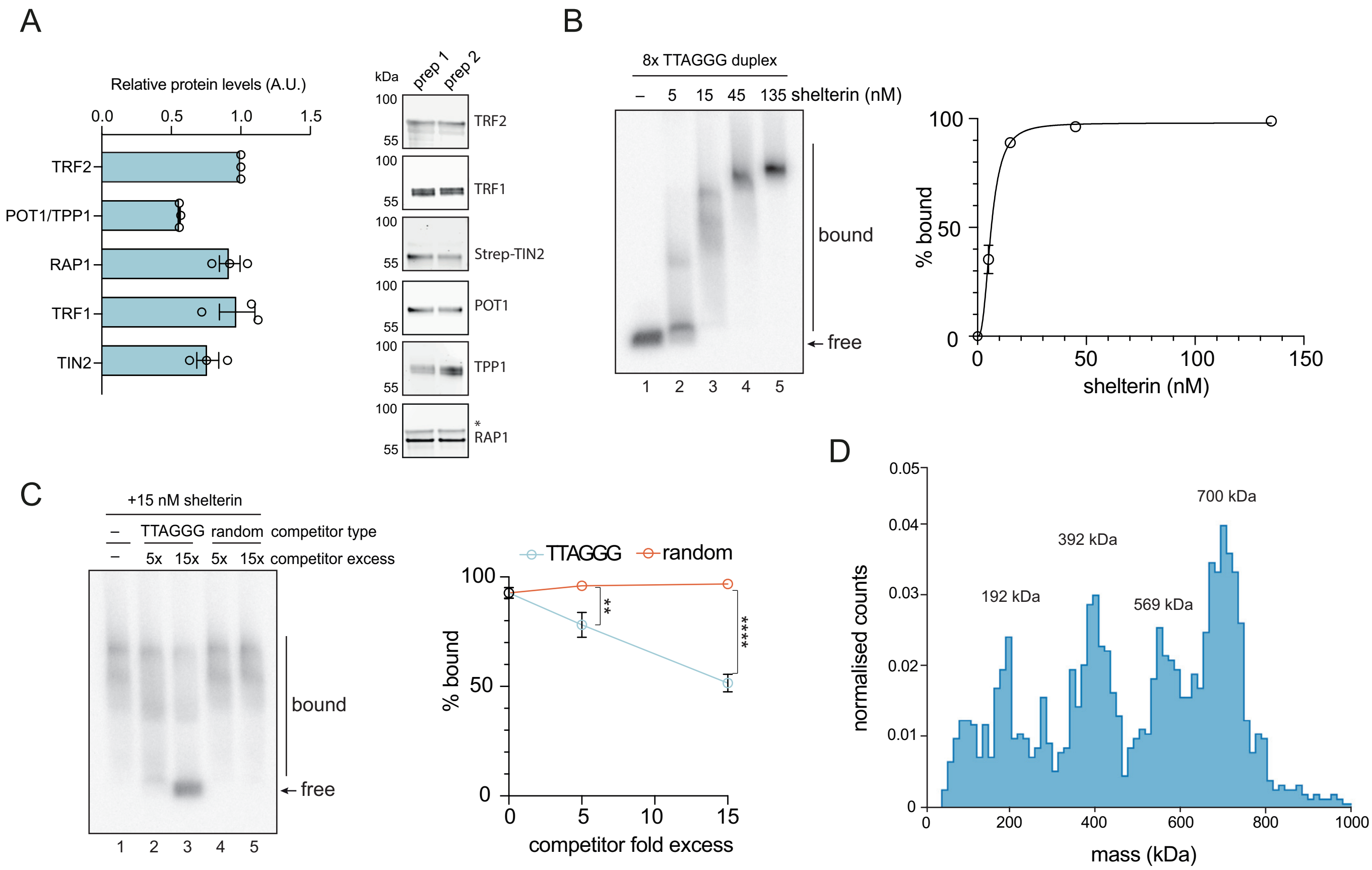

Figure EV3. Supplementary data for Fig. 3.

(**A**) Left side: quantification of the relative levels of each protein band of shelterin following SDS-PAGE as in Fig. 3A. Signal adjusted for protein molecular weight and presented relative to TRF2. Error bars show the S.E.M. $N = 3$ (three independent shelterin preparations). Right side: immunoblot of two independent shelterin preparations separated by 4–15% Tris-Glycine SDS-PAGE using the indicated antibodies. Asterisk marks a band deriving from additional detection of TRF2. (**B**) EMSA analysis of purified 6-membered shelterin binding to a 100 bp duplex containing 8x TTAGGG repeats at the indicated concentrations. Products separated by native agarose electrophoresis. Nonlinear regression (Hill equation) was used to fit the data. Kd = 6.24 nM. Error bars show the S.E.M. $N = 3$ (three independent EMSA). (**C**) EMSA analysis as in B was performed in the presence of 15 nM shelterin and a 5- or 15-times molar excess of unlabelled telomeric or control sequence. Products separated by native agarose electrophoresis. Two-way ANOVA was used to determine statistical significance. Error bars show the S.E.M. $N = 3$ (three independent EMSA). Corresponding *P* values: control vs telomeric 5× (0.0064), control vs telomeric 15× (1.19 × 106). (**D**) Mass photometry of shelterin with peak maxima indicated.

© The Author(s)

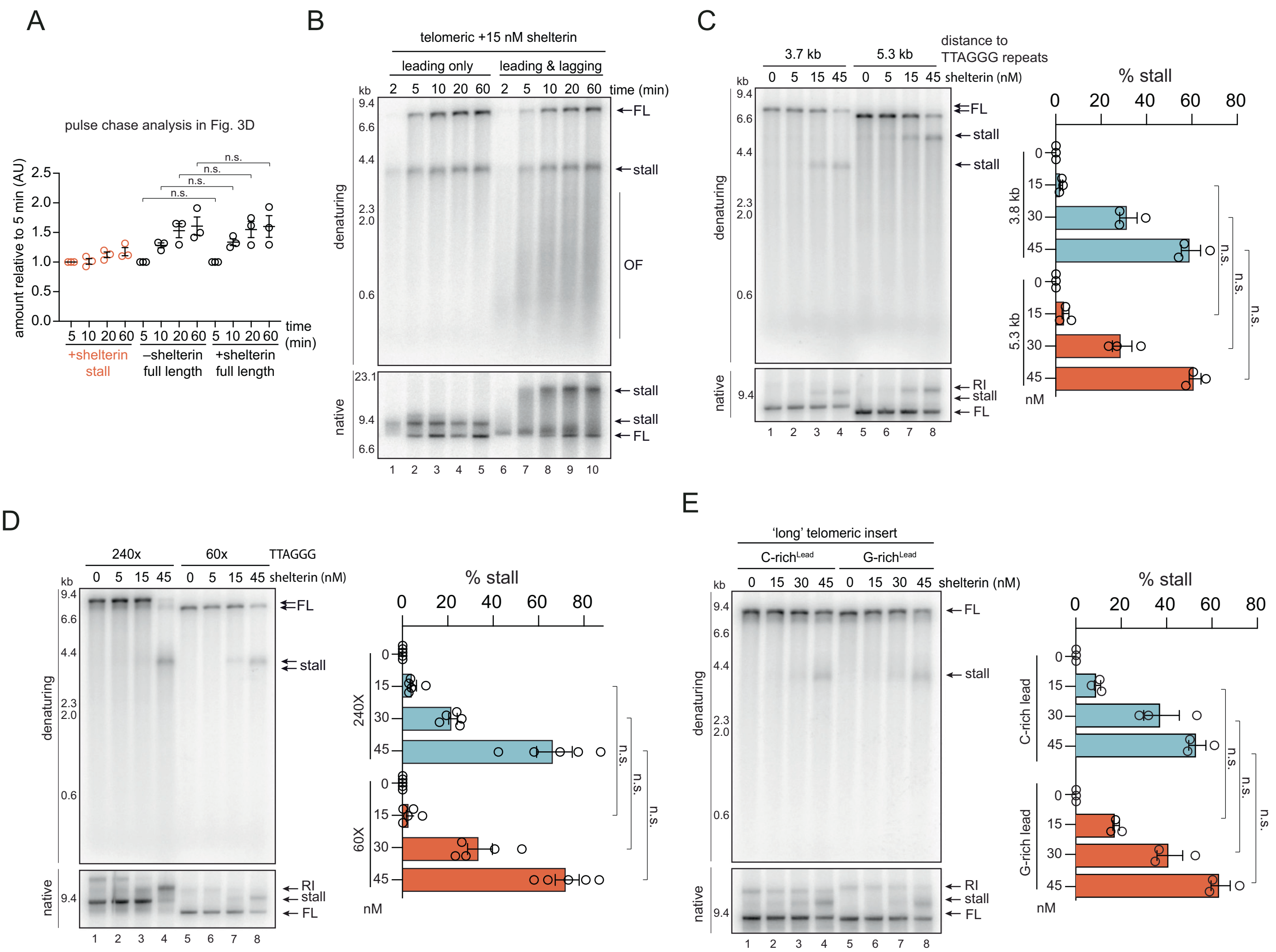

Figure EV4. Supplementary data for Fig. 3.

(**A**) Quantification of the relative intensities of telomere-stalled or full-length products over time after denaturing alkaline agarose as in Fig. 3B. Stall and FL signal was calculated relative to 5 min time point. Two-way ANOVA was used to determine statistical significance. Error bars show the S.E.M. $N = 3$ (three independent replication assays). ($P = 0.99$). (**B**) Replication reactions with leading (left) or leading and lagging strand synthesis (right) were performed with a template containing 60 TTAGGG repeats in presence of 15 nM shelterin for indicated times. Products were analysed by native and denaturing alkaline agarose electrophoresis as indicated. (**C**) Replication reactions were performed for 20 min with templates containing 60 TTAGGG repeats positioned 3.8 or 5.3 kb from the site of replication initiation with the indicated concentrations of shelterin. Products were analysed as in (**B**). Quantification of stall signal after denaturing alkaline agarose. Stall signal was calculated relative to no shelterin. Two-way ANOVA was used to determine statistical significance. Error bars show the S.E.M. $N = 3$ (three independent replication assays). Corresponding P values: 5 nM (0.9851), 15 nM (0.9324), 45 nM (0.9850). (**D**) Products from replication reactions performed for 20 min with telomeric template containing 240× or 60× repeats with the indicated concentrations of shelterin were analysed as in (**B**). Stall signal was calculated relative to no shelterin. Two-way ANOVA was used to determine statistical significance. Error bars show the S.E.M. $N = 5$ (five independent replication assays). Corresponding P values: 5 nM (0.9987), 15 nM (0.1803), 45 nM (0.9888). (**E**) Products from replication reactions performed for 20 min with templates containing 240 TTAGGG repeats oriented with C- or G-rich telomeric DNA as the leading strand template with the shelterin concentrations indicated were analysed as in B. Stall signal was calculated relative to no shelterin. Two-way ANOVA was used to determine statistical significance. Error bars show the S.E.M. $N = 3$ (three independent replication assays). Corresponding P values: 15 nM (0.5240), 30 nM (0.9501), 45 nM (0.3150).

 © The Author(s)

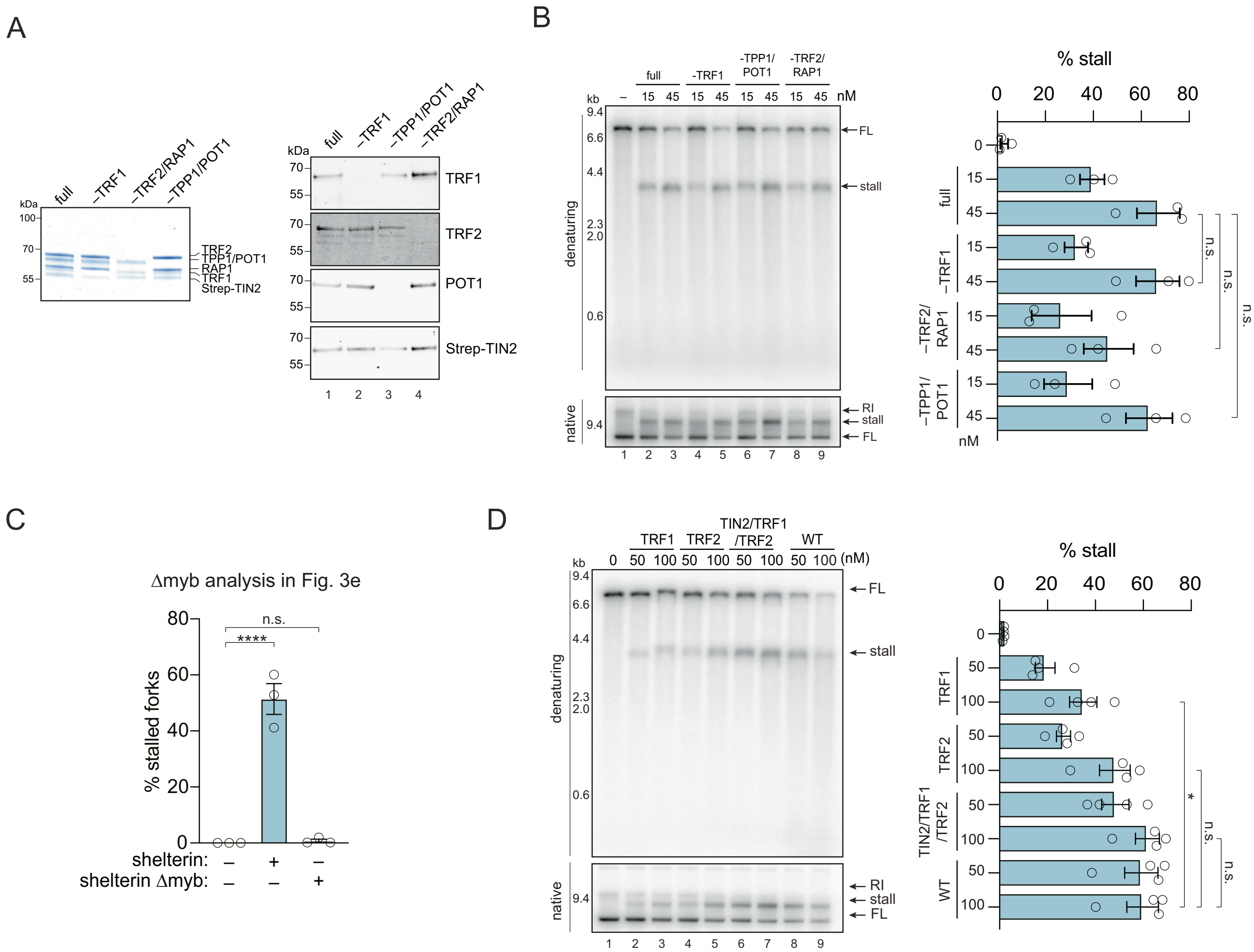

Figure EV5. Supplementary data for Fig. 3.

(**A**) Left side: 4–15% Tris-Glycine SDS-PAGE analysis of purified shelterin variants. Right side: Immunoblot of each purified shelterin variant with the antibodies indicated verifying loss of specific subunits expected. (**B**) Products from replication reactions performed for 20 min on a template containing 60 TTAGGG repeats in the presence or absence of shelterin or the shelterin variants indicated were analysed by native and denaturing alkaline agarose electrophoresis. One-way ANOVA was used to determine statistical significance. Error bars show the S.E.M. $N = 3$ (three independent replication assays). Corresponding P values: 45 nM full vs -TRF1 (0.9999), 45 nM full vs -TRF2/RAP1 (0.5519), 45 nM full vs -TPP1/POT1 (0.9997). (**C**) Quantification of Fig. 3E. Stall signal was calculated relative to no shelterin. One-way ANOVA was used to determine statistical significance. Error bars show the S.E.M. $N = 3$ (three independent replication assays). Corresponding P values: no shelterin vs full shelterin (5.07×10^5), no shelterin vs shelterin ΔMyb (0.9806). (**D**) Products from replication reactions performed for 20 min on a template containing 60 TTAGGG repeats in the presence or absence of the indicated proteins were analysed as in (**B**). One-way ANOVA was used to determine statistical significance. Error bars show the S.E.M. $N = 4$ (four independent replication assays). Corresponding P values: 100 nM WT vs TRF1 (0.0155), 100 nM WT vs TRF2 (0.5131), 100 nM WT vs TRF1/TRF2/TIN2 (0.9999).

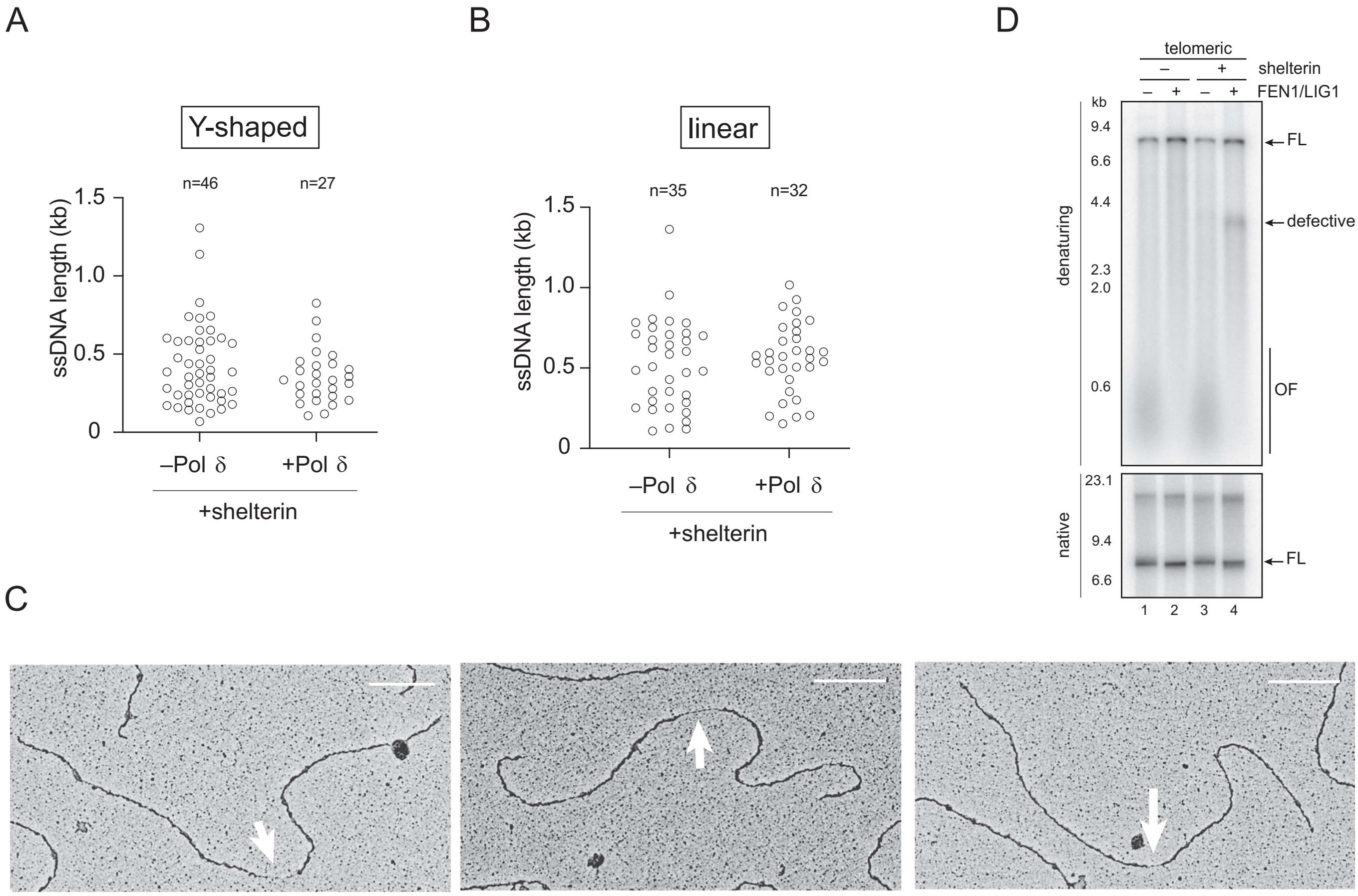

Figure EV6. Supplementary data for Fig. 4.

(**A**) Graph showing the length of single stranded gaps on Y-shaped fork structures measures by EM in the presence of shelterin and presence or absence of Pol δ as in Fig. 4B. (**B**) Graph showing the length of single stranded gaps measured on linear molecules in the presence of shelterin and presence or absence of Pol δ as shown in (**C**). (**C**) Electron micrographs showing linear DNA molecules with an internal tract of single stranded DNA at the position of telomeric DNA (white arrows) from reactions performed on a template containing 60 TTAGGG repeats in the presence of shelterin and absence of Pol δ. Scale bar represents 1 kb. (**D**) Replication reactions performed for 30 min on a template containing 60 TTAGGG repeats with shelterin and FEN1/LIG1 as indicated were analysed by native and denaturing agarose electrophoresis.

 © The Author(s)

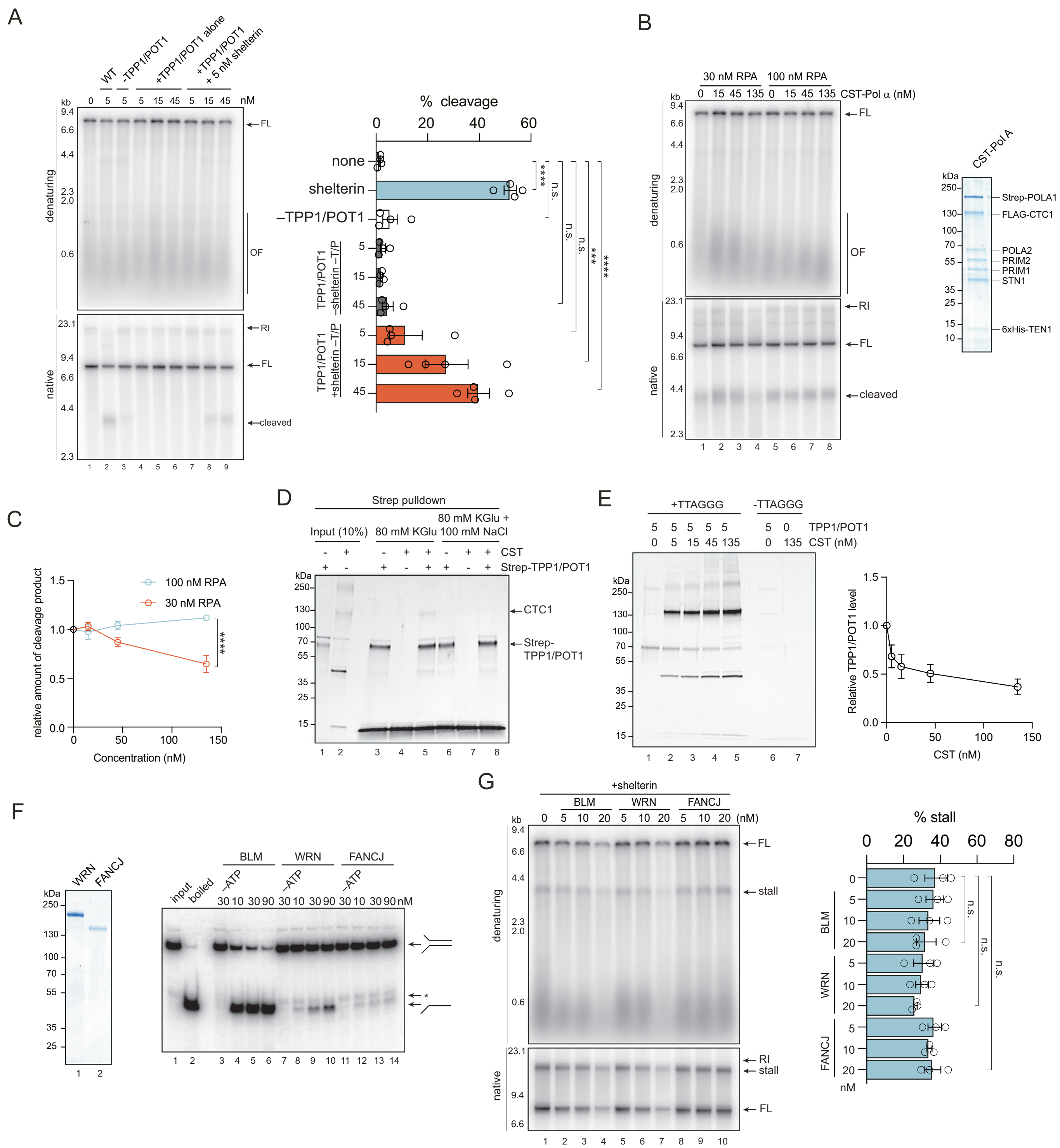
A
WT
-TPP1/POT1
+TPP1/POT1 alone
+TPP1/POT1 + 5 nM shelterin
% cleavage
none
shelterin
–TPP1/POT1
TPP1/POT1 –shelterin –T/P
TPP1/POT1 +shelterin –T/P
denaturing
native
FL
OF
RI
cleaved
B
30 nM RPA
100 nM RPA
CST-Pol α (nM)
CST-Pol A
Strep-POLA1
FLAG-CTC1
POLA2
PRIM2
PRIM1
STN1
6xHis-TEN1
C
100 nM RPA
30 nM RPA
relative amount of cleavage product
Concentration (nM)
D
Strep pulldown
Input (10%)
80 mM KGlu
80 mM KGlu + 100 mM NaCl
CST
Strep-TPP1/POT1
CTC1
Strep-TPP1/POT1
E
+TTAGGG
-TTAGGG
TPP1/POT1
CST (nM)
Relative TPP1/POT1 level
CST (nM)
F
WRN
FANCJ
input
boiled
BLM
WRN
FANCJ
–ATP
G
+shelterin
BLM
WRN
FANCJ
(nM)
FL
stall
RI
% stall
n.s.

Figure EV7. Supplementary data for Fig. 5.

(**A**) Replication reactions performed for 15 min on a template containing 60 TTAGGG repeats with either wild-type shelterin, shelterin -TPP1/POT1, TPP1/POT1 alone or TPP1/POT1 with 5 nM shelterin -TPP1/POT1 added. 0.24 U/µl P1 nuclease was added 5 min before quenching and products were analysed by native and denaturing alkaline agarose electrophoresis. One-way ANOVA was used to determine statistical significance. Error bars show the S.E.M. $N = 4$ (four independent replication assays). Corresponding P values: none vs shelterin (1.45×10^{8}), none vs -TPP1/POT1 (0.9791), none vs TPP1/POT1 alone 5 nM (0.9999), 15 nM (0.9999), 45 nM (0.9958), none vs TPP1/POT1 with shelterin -TPP1/POT1 5 nM (0.3891), 15 nM (0.0008), 45 nM (2.59×10^{6}). (**B**) Right side: 4–15% Tris-Glycine SDS-PAGE analysis of purified CST-Pol α/prim complex. Left side: Replication reactions were performed for 15 min on a template containing 60 TTAGGG repeats with 5 nM shelterin and the indicated concentrations of CST-Pol α/prim complex and either 30 or 100 nM RPA. 0.035 U/µl P1 nuclease was added and products analysed as in (**A**). (**C**) Quantification of the level of P1 cleavage product from reactions as in (**B**). Level of P1 cleavage was relative to reactions containing no CST-Pol α/prim. Two-way ANOVA was used to determine statistical significance. Error bars show the S.E.M. $N = 3$ (three independent replication assays). $P = 2.02 \times 10^{5}$. (**D**) Pulldown assay with TPP1/POT1 precipitated via a strep tag on TPP1 in the presence of CST as indicated. Bound complexes were washed in buffer containing the indicated salts. Final complexes were separated by 4–15% Tris-Glycine SDS-PAGE before analysis by silver staining. (**E**) Pulldown assay with immobilised single stranded 3× TTAGGG repeats and the indicated concentrations of TPP1/POT1 and CST. Final complexes were separated by 4–15% Tris-Glycine SDS-PAGE before analysis by silver staining. The relative level of TPP1/POT1 is shown. $N = 4$ (four independent pulldown assays). Error bars show S.E.M. (**F**) Left side: 4–15% Tris-Glycine SDS-PAGE analysis of purified wild-type WRN and FANCJ. Right side: products from a helicase assay performed for 15 min on 1.5 nM duplexed substrate with the indicated concentrations of BLM, WRN or FANCJ in the presence of 10 nM RPA were analysed by native agarose electrophoresis. Asterisk indicates a non-specific band also in the input. (**G**) Products from replication reactions performed for 20 min on 60 TTAGGG telomeric templates presence of 15 nM shelterin with the indicated concentrations of helicases were analysed as in A. One-way ANOVA was used to determine statistical significance. Error bars show the S.E.M. $N = 3$ (three independent replication assays). Corresponding P values: 0 vs 20 nM BLM (0.9536), 0 vs 20 nM WRN (0.4048), 0 vs 20 nM FANCJ (0.9999).

 © The Author(s)
